# Supplementary material for: Single-cell spatial transcriptomics reveals a dynamic control of metabolic zonation and liver regeneration by endothelial cell Wnt2 and Wnt9b
Source: Cell Rep Med. 2022 Oct 10;3(10):100754. doi: 10.1016/j.xcrm.2022.100754 (PMC9588996; doi:10.1016/j.xcrm.2022.100754)
Supplement: Document S1. Figures S1–S17 and Tables S1–S4 [file mmc1.pdf]

Cell Reports Medicine, Volume *3*

Supplemental information

Single-cell spatial transcriptomics reveals a dynamic control of metabolic zonation and liver regeneration by endothelial cell Wnt2 and Wnt9b

Shikai Hu, Silvia Liu, Yu Bian, Minakshi Poddar, Sucha Singh, Catherine Cao, Jackson McGaughey, Aaron Bell, Levi L. Blazer, Jarret J. Adams, Sachdev S. Sidhu, Stephane Angers, and Satdarshan P. Monga

Supplementary Figures and Figure Legends

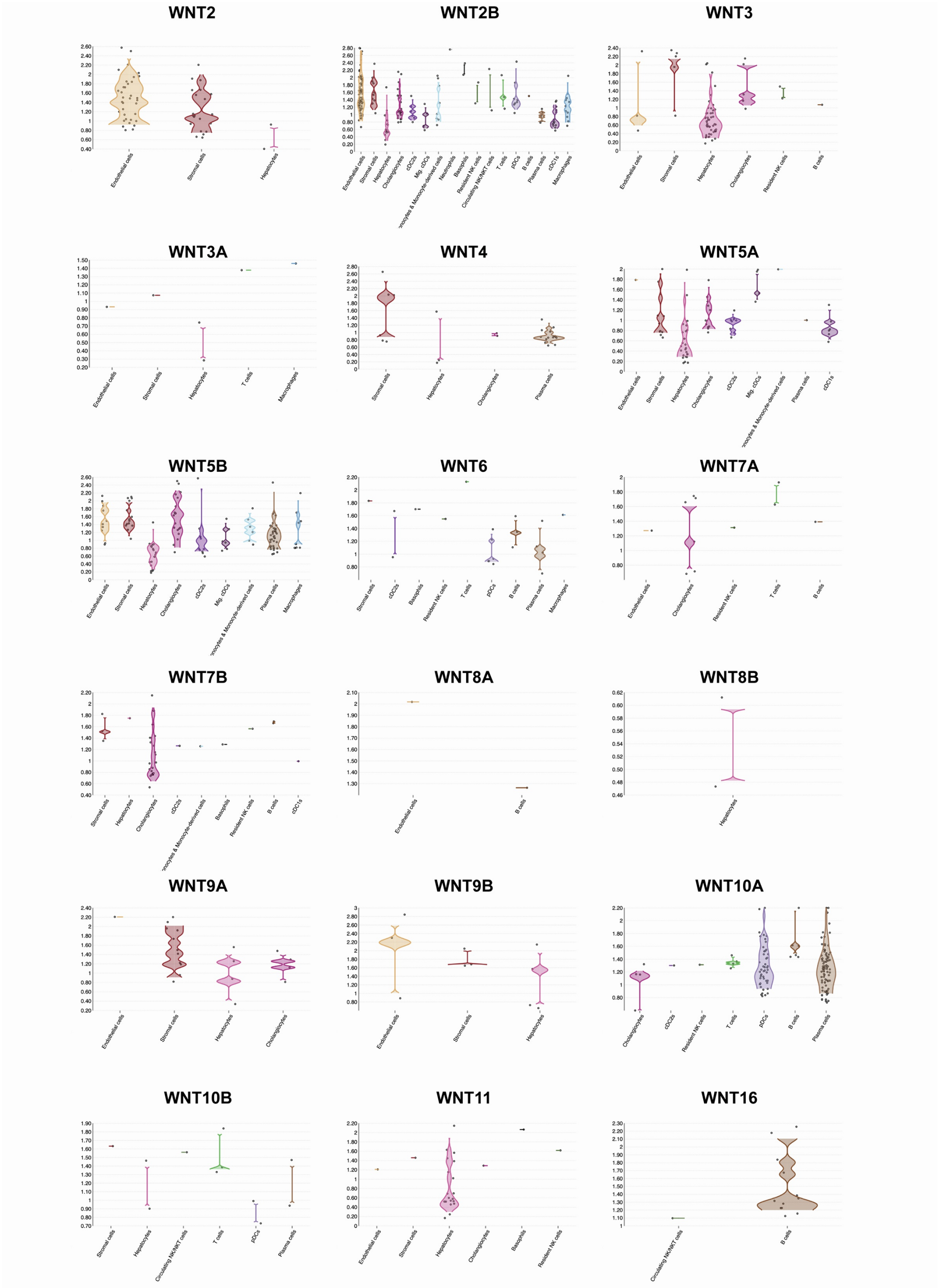

Fig. S1: Violin plots showing expression level of WNTs in normal human liver. (Related to Figure 1)

Eighteen WNTs were detected by scRNA seq. ECs were the predominant source of WNT2, WNT2B, and WNT9B.

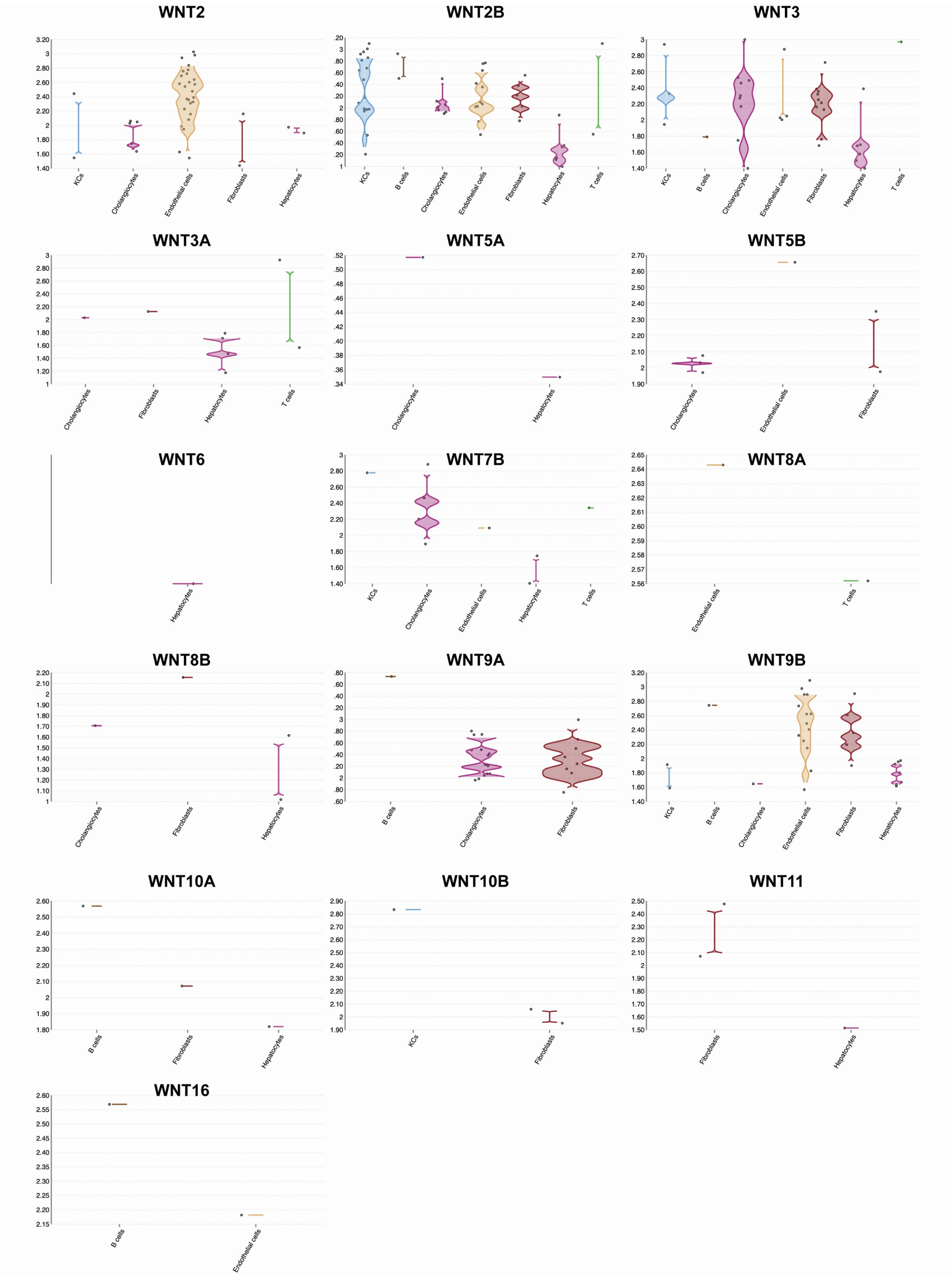

Fig. S2: Violin plots showing expression level of WNTs in monkey liver. (Related to Figure 1)

Sixteen WNTs were detected. ECs expressed high levels of WNT2 and WNT9B.

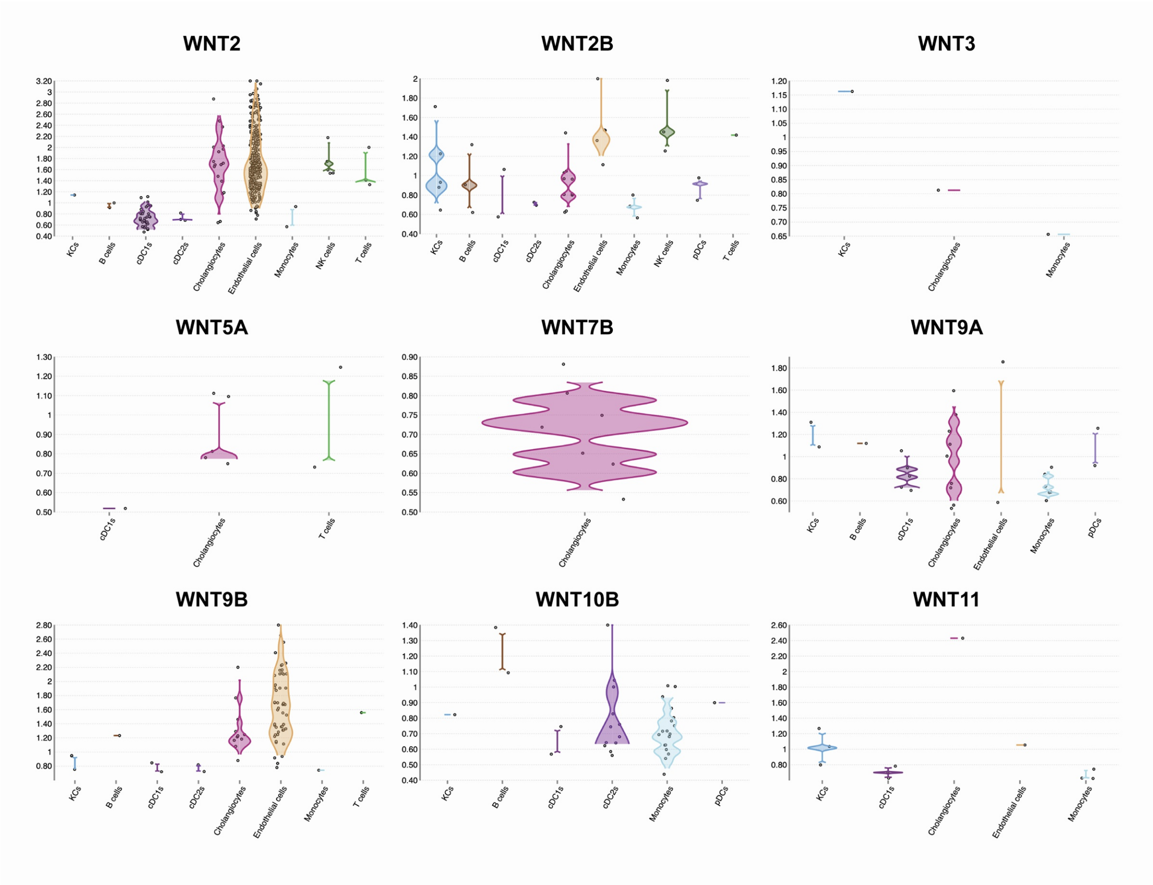

Fig. S3: Violin plots showing expression level of WNTs in pig liver. (Related to Figure 1)

Nine WNTs were detected. ECs expressed high levels of WNT2 and WNT9B.

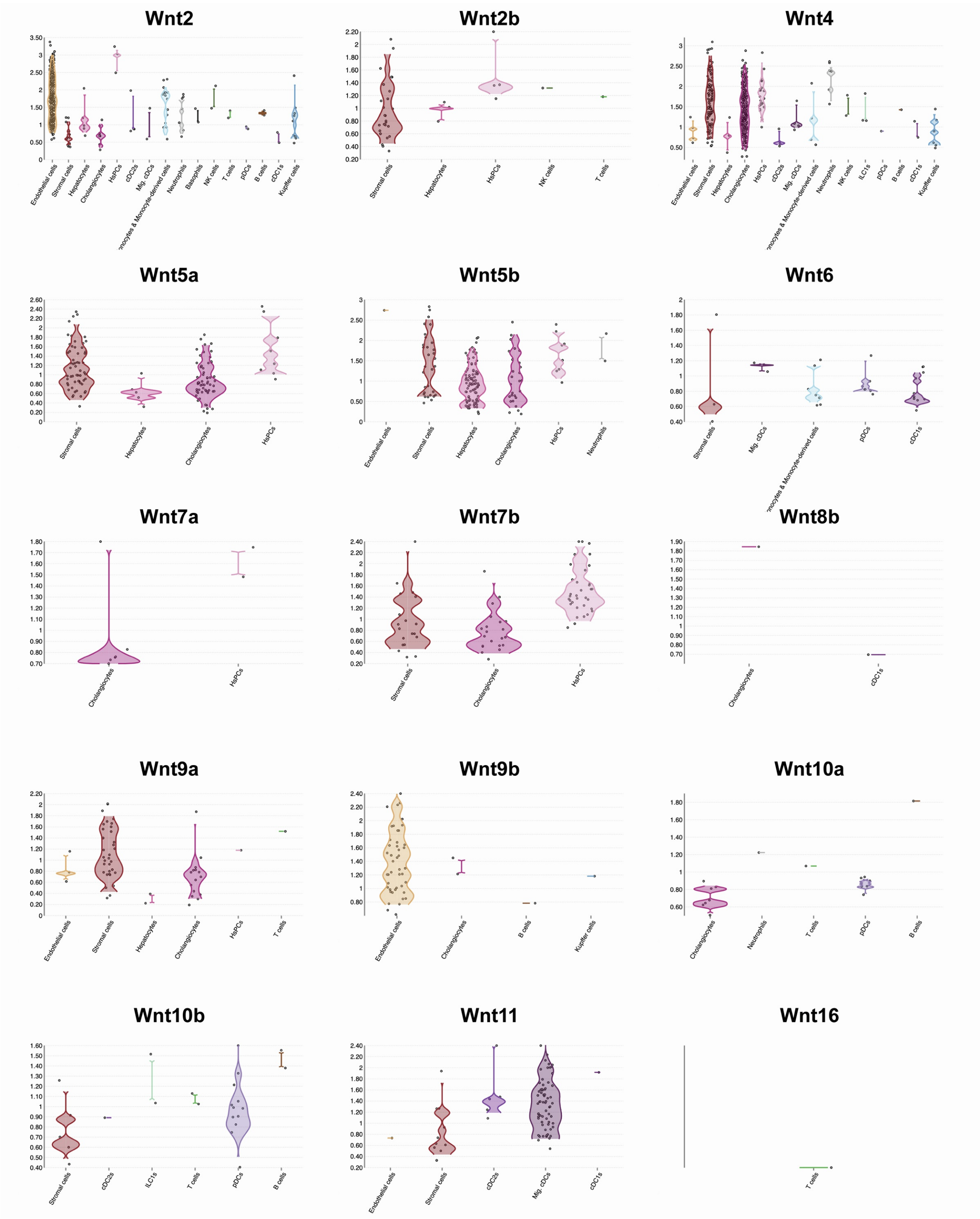

Fig. S4: Violin plots showing expression level of Wnts in C57BL/6 mice liver.
(Related to Figure 1)
Fifteen Wnts were detected. ECs expressed high levels of Wnt2 and Wnt9b.

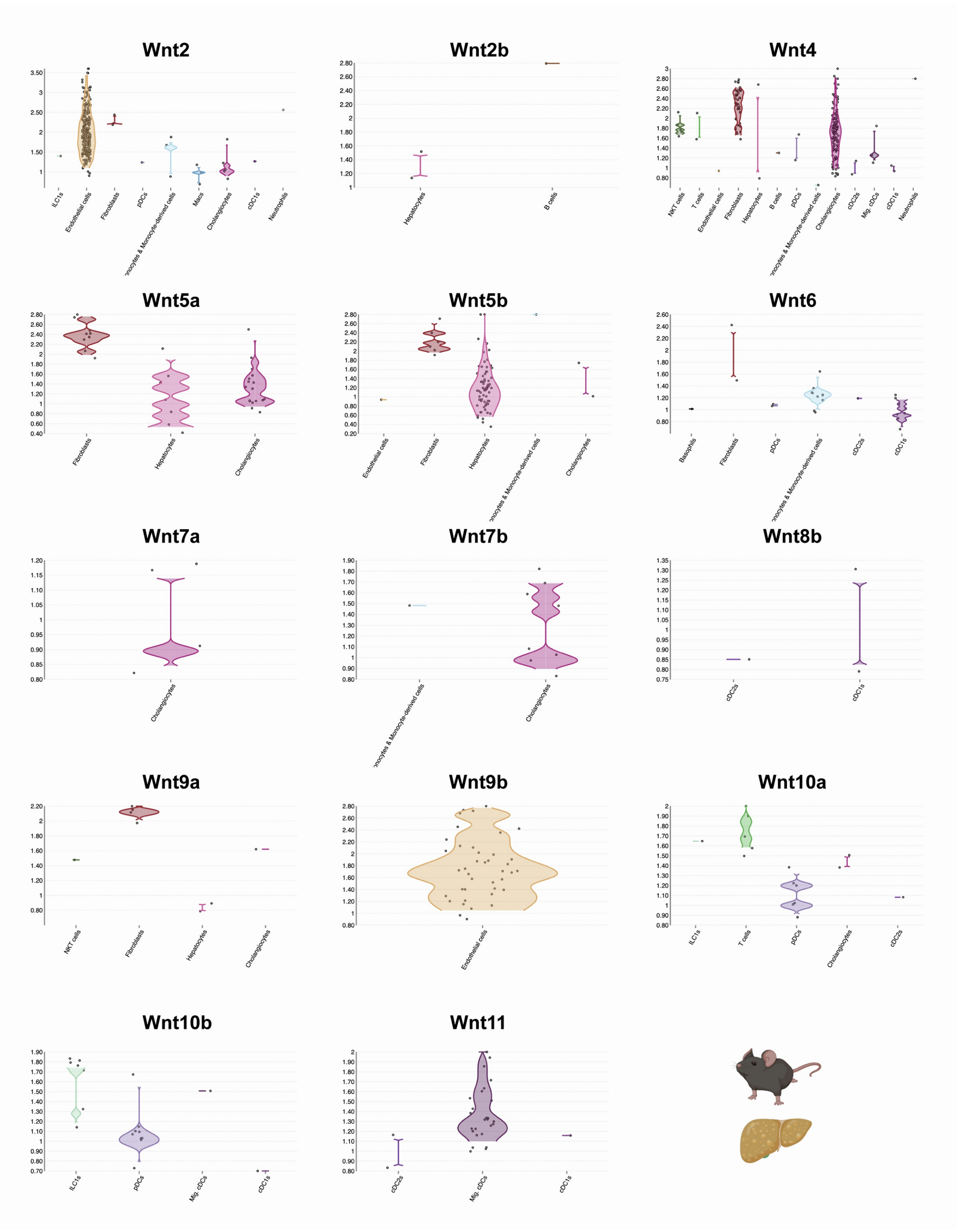

Fig. S5: Violin plots showing expression level of Wnts in NAFLD mice liver.
(Related to Figure 1)

Fourteen Wnts were detected. Endothelial expression of Wnt2 and Wnt9b was maintained in NAFLD mice. (Cartoons were created with BioRender.com)

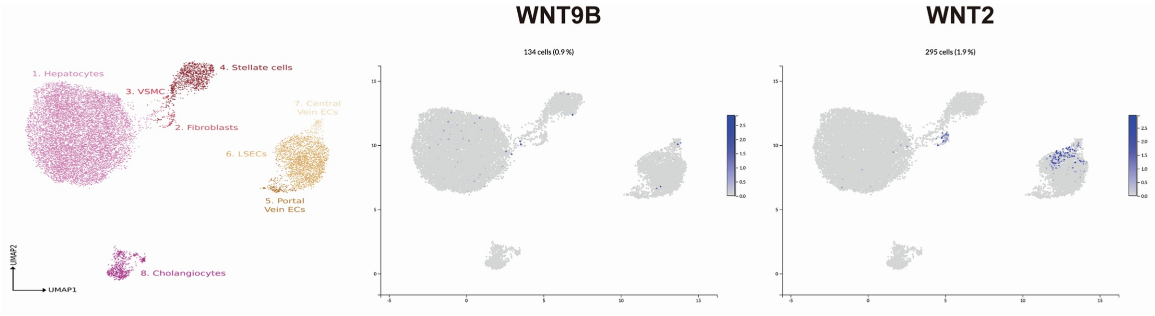

Fig. S6: Feature plots showing expression and distribution of WNTs in human liver. (Related to Figure 1)

WNT2 was pericentrally zonated in human ECs.

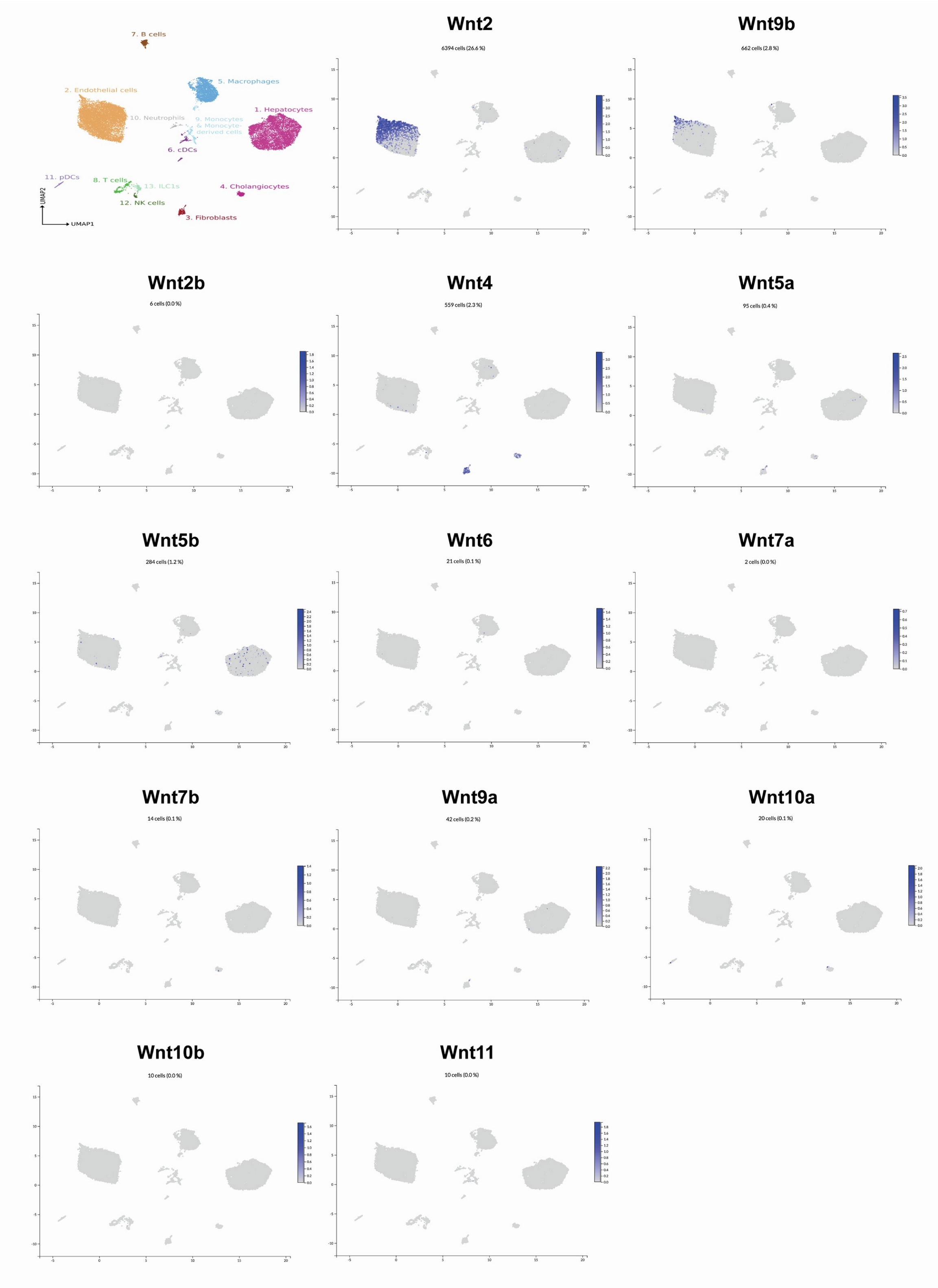

Fig. S7: Feature plots showing expression and distribution of Wnts in C57BL/6 mice liver using single nuclei RNA sequencing. (Related to Figure 1)
Among thirteen detected Wnts, only Wnt2 and Wnt9b were pericentrally zonated by snRNA seq.

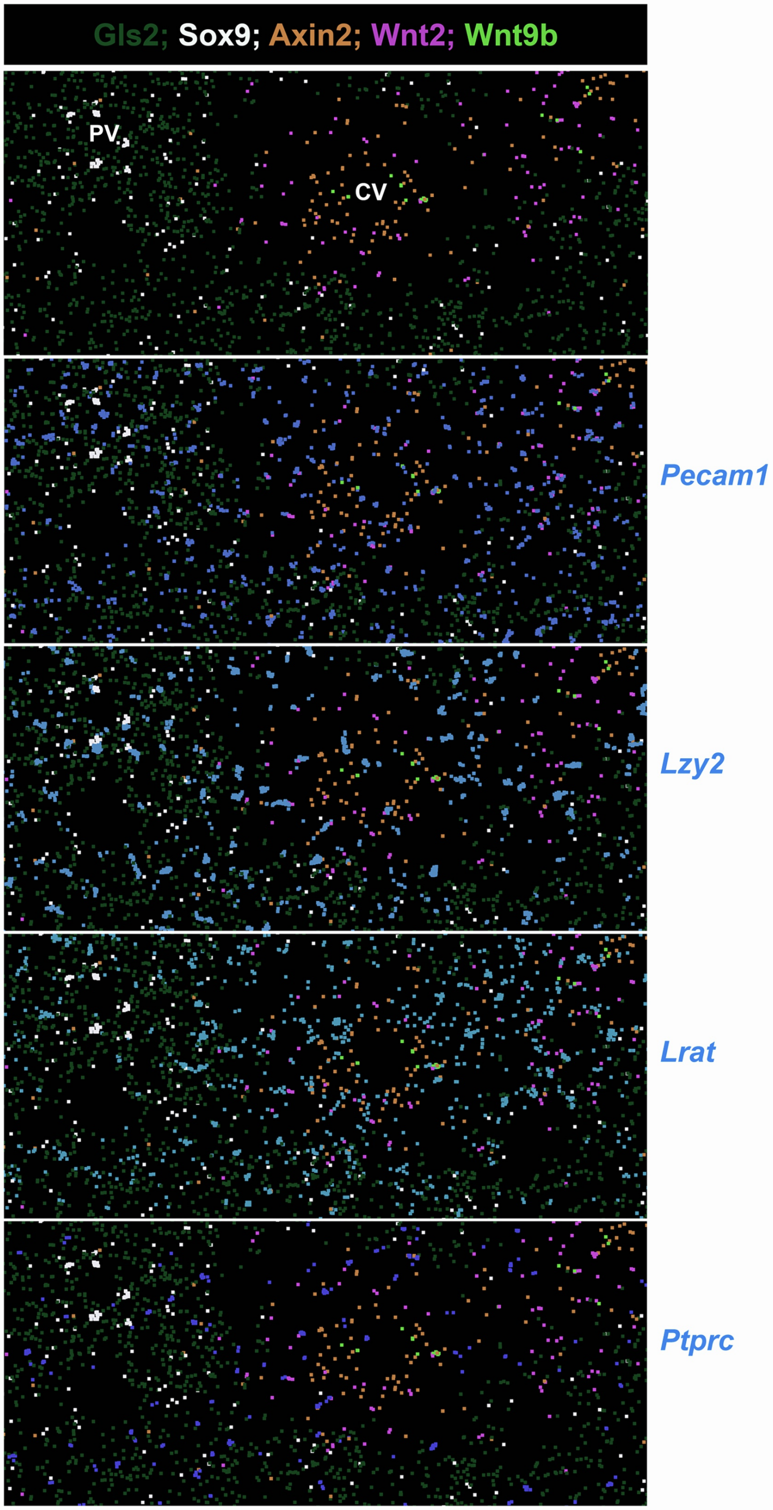

Fig. S8: Molecular Cartography of indicated genes. (Related to Figure 1)
Colocalization of *Wnt2* and *Wnt9b* with cell-specific markers including *Pecam1* for ECs, *Lyz2* for macrophages, *Lrat* for hepatic stellate cells, and *Ptprc* for immune cells. *Wnt2* and *Wnt9b* predominantly colocalized with *Pecam1*, while some overlap was also evident with *Lyz2*, *Lrat*, and *Ptprc*. (CV: Central vein; PV: portal vein)

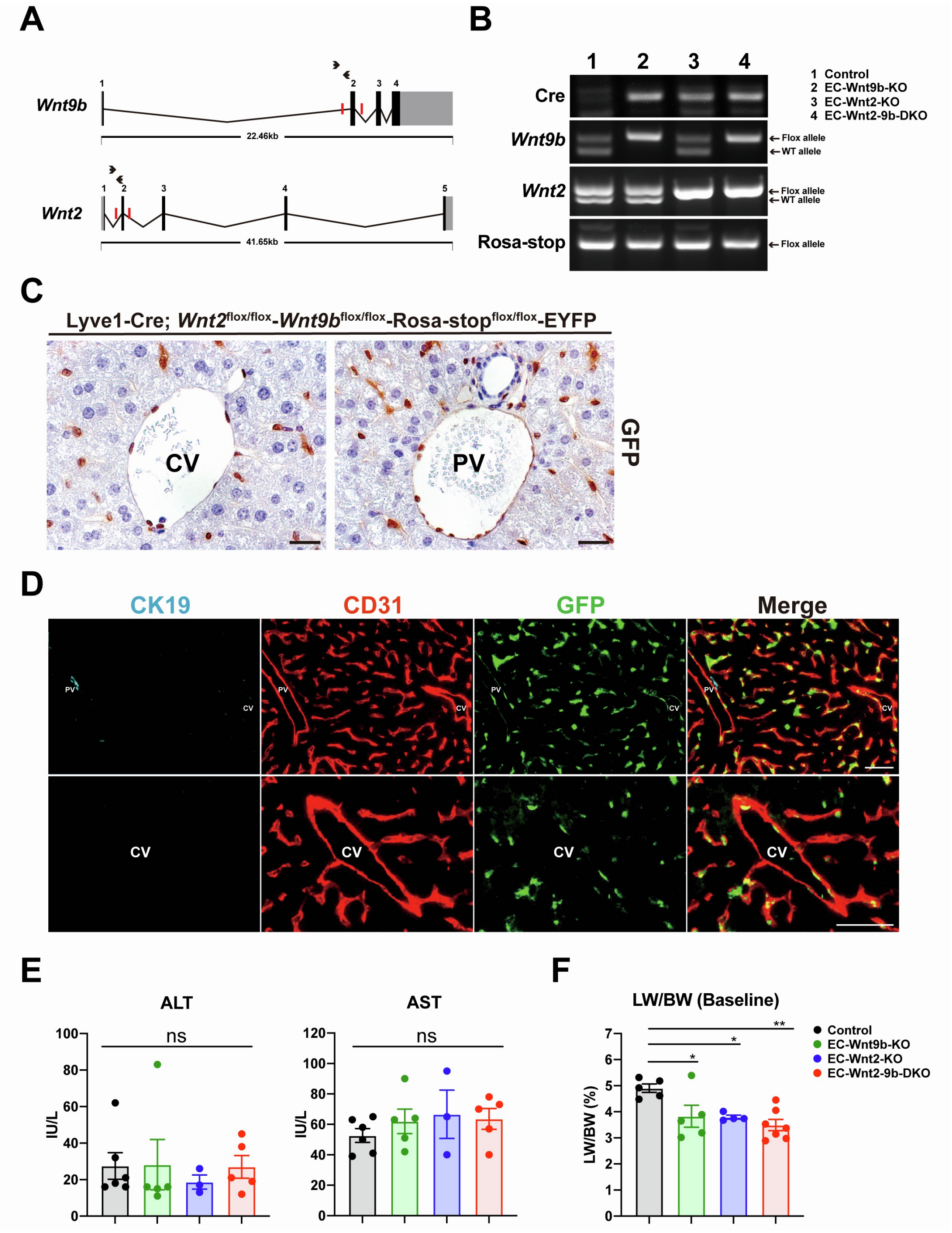

Fig. S9: Characterization of mice models. (Related to Figure 2)

(A) Scheme depicting loxP site (Red lines) in mice *Wnt9b* and *Wnt2* gene. (Black boxes: exons; grey boxes: 5'-UTR and 3'-UTR non-coding regions; arrow heads: PCR primers for genotyping)

(B) Gel electrophoresis showing generation of mice as indicated in the figure.

(C, D) IHC and immunofluorescence of EC-Wnt2-9b-DKO mice showing hepatic sinusoidal ECs and central venous ECs were positive for GFP. (CV: central vein; PV: portal vein. Scale bars for IHC: 20 μm; Scale bars for IF: 100 μm)

(E) No baseline liver injury was observed by serum levels of ALT and AST. (n = 6, 5, 3, 5 mice)

(F) LW/BW (+/-SEM) was lower in EC-Wnt2-KO and EC-Wnt9b-KO mice, and even lower in EC-Wnt2-9b-DKO mice. (ns = not significant, *P < 0.05, **P < 0.01. n = 5, 5, 4, 7 mice)

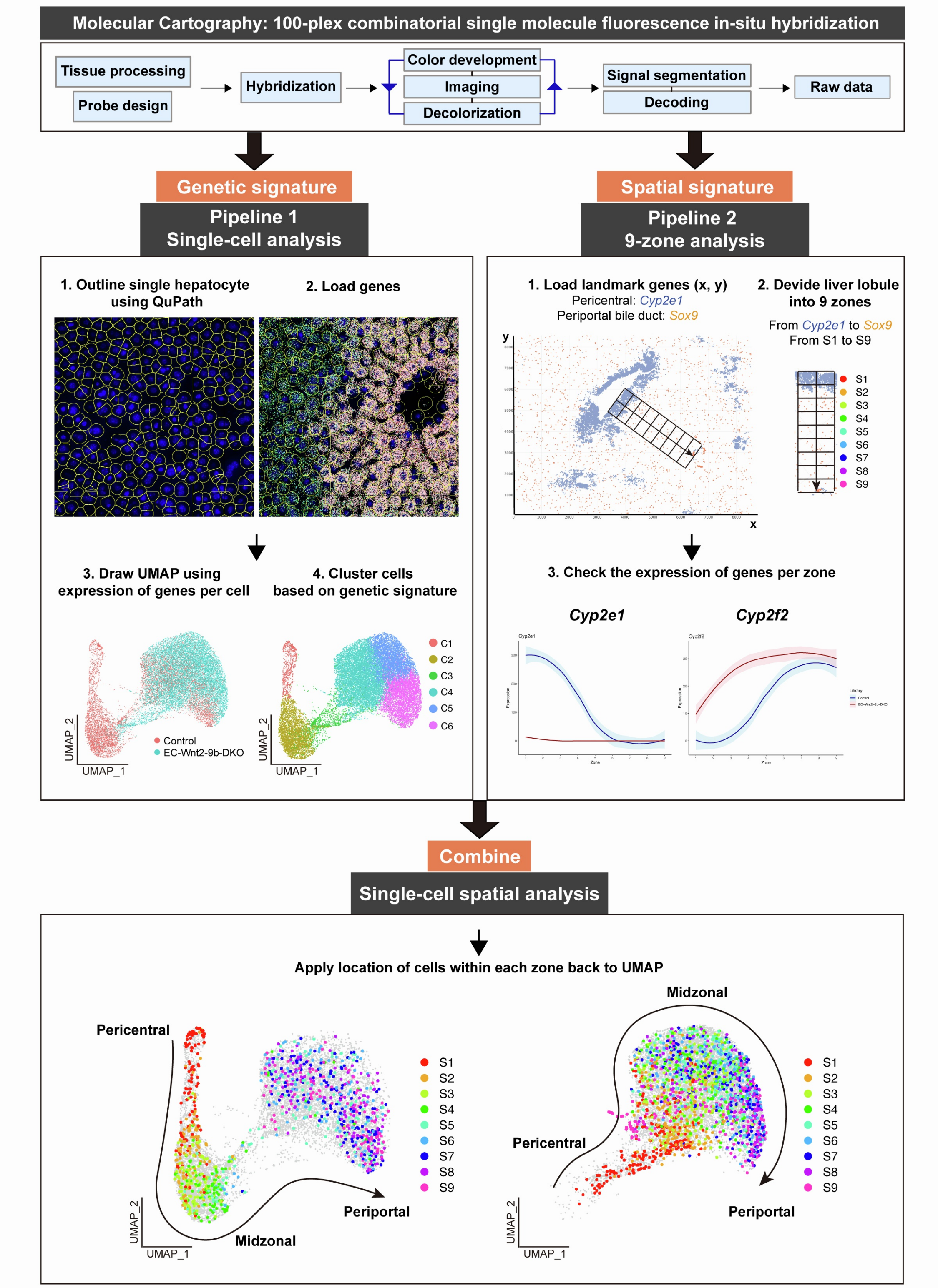

Fig. S10: Workflow of single-cell transcriptomics analysis of Molecular Cartography data of EC-Wnt2-9b-DKO mice. (Related to Figure 3 and STAR Methods)

See STAR Methods for details.

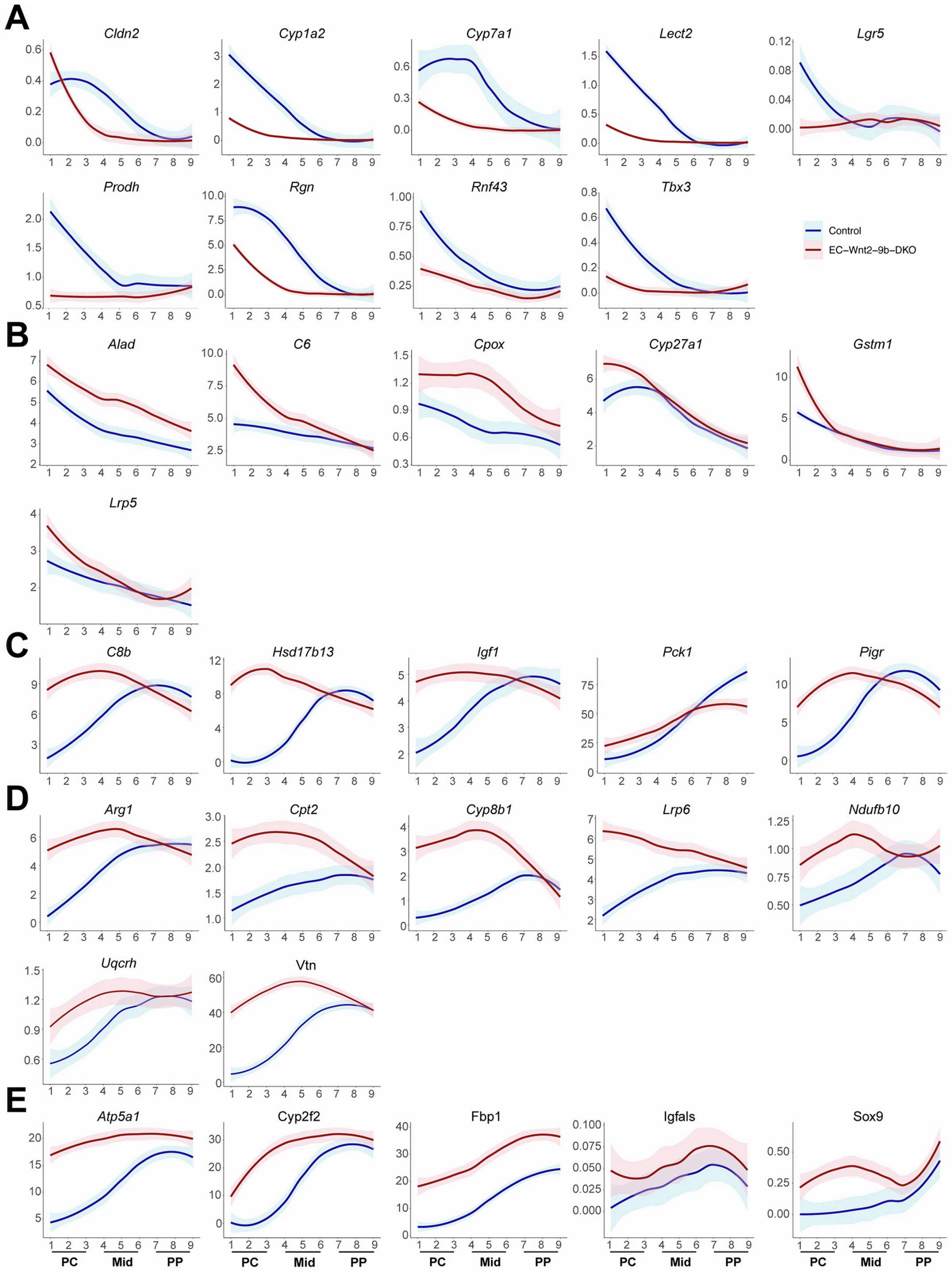

Fig. S11: Line plots showing changes of zonated genes between control and EC-Wnt2-9b-DKO mice. (Related to Figure 3)

(A) Pericentral genes that decreased in EC-Wnt2-9b-DKO mice.
(B) Pericentral genes that increased in EC-Wnt2-9b-DKO mice.
(C) Periportal genes that had decreased zone 1 gene expression in EC-Wnt2-9b-DKO mice.
(D) Periportal genes that had similar or unchanged zone 1 gene expression in EC-Wnt2-9b-DKO mice.
(E) Periportal genes that had increased zone 1 gene expression in EC-Wnt2-9b-DKO mice. The hue around line plots represents SEM.

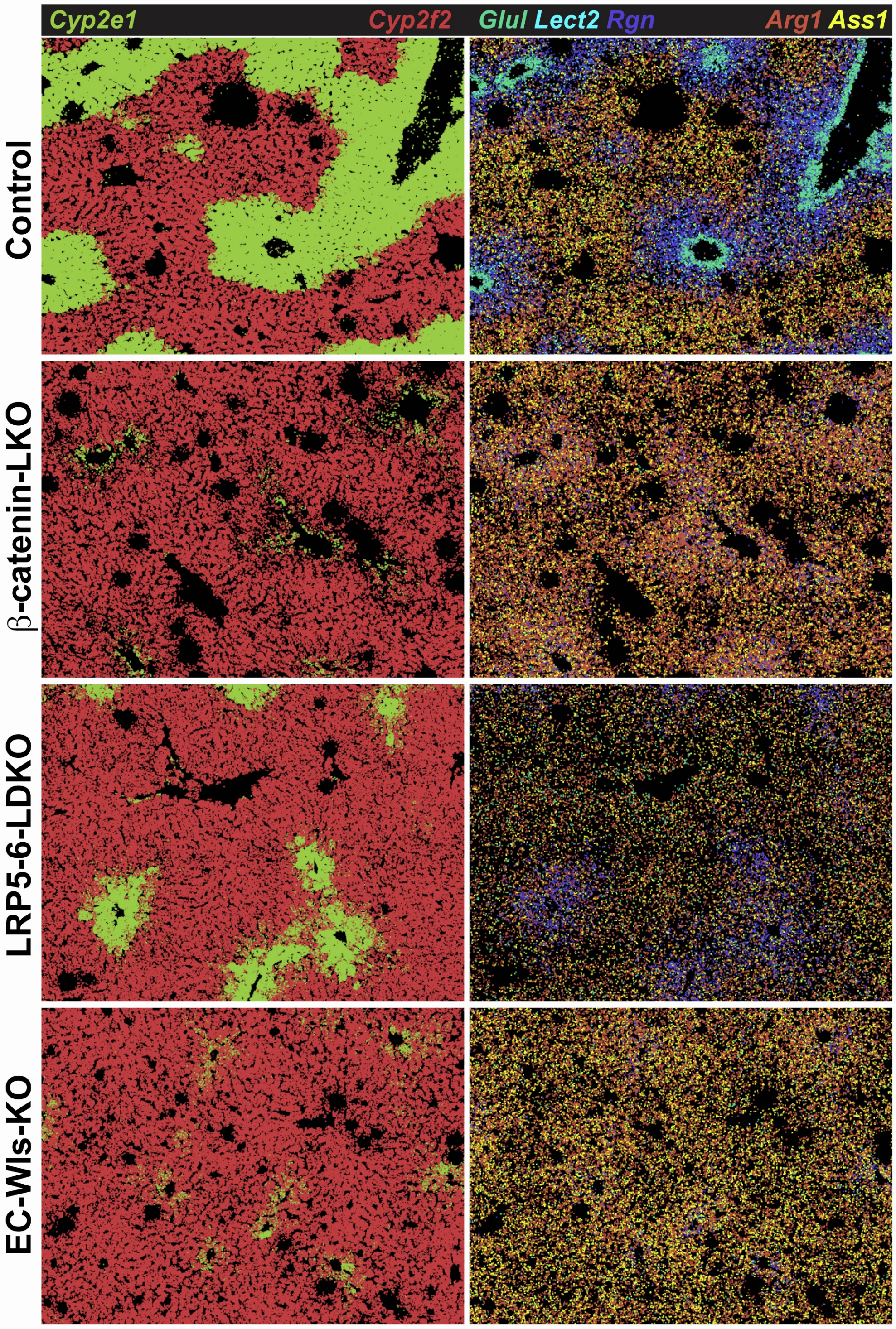

Fig. S12: Molecular Cartography images showing periportalization of liver lobule in various animal models of disrupted Wnt-β-catenin signaling (Related to Figure 3).

Representative images from Molecular Cartography analysis of selected genes in the livers from control, hepatocyte-specific β-catenin KO (β-catenin-LKO), hepatocyte-specific LRP5-6 DKO (LRP5-6-LDKO) and endothelial cell Wntless KO (EC-Wls-KO) showing gain of periportal genes in pericentral hepatocytes at the expense of pericentral β-catenin target genes.

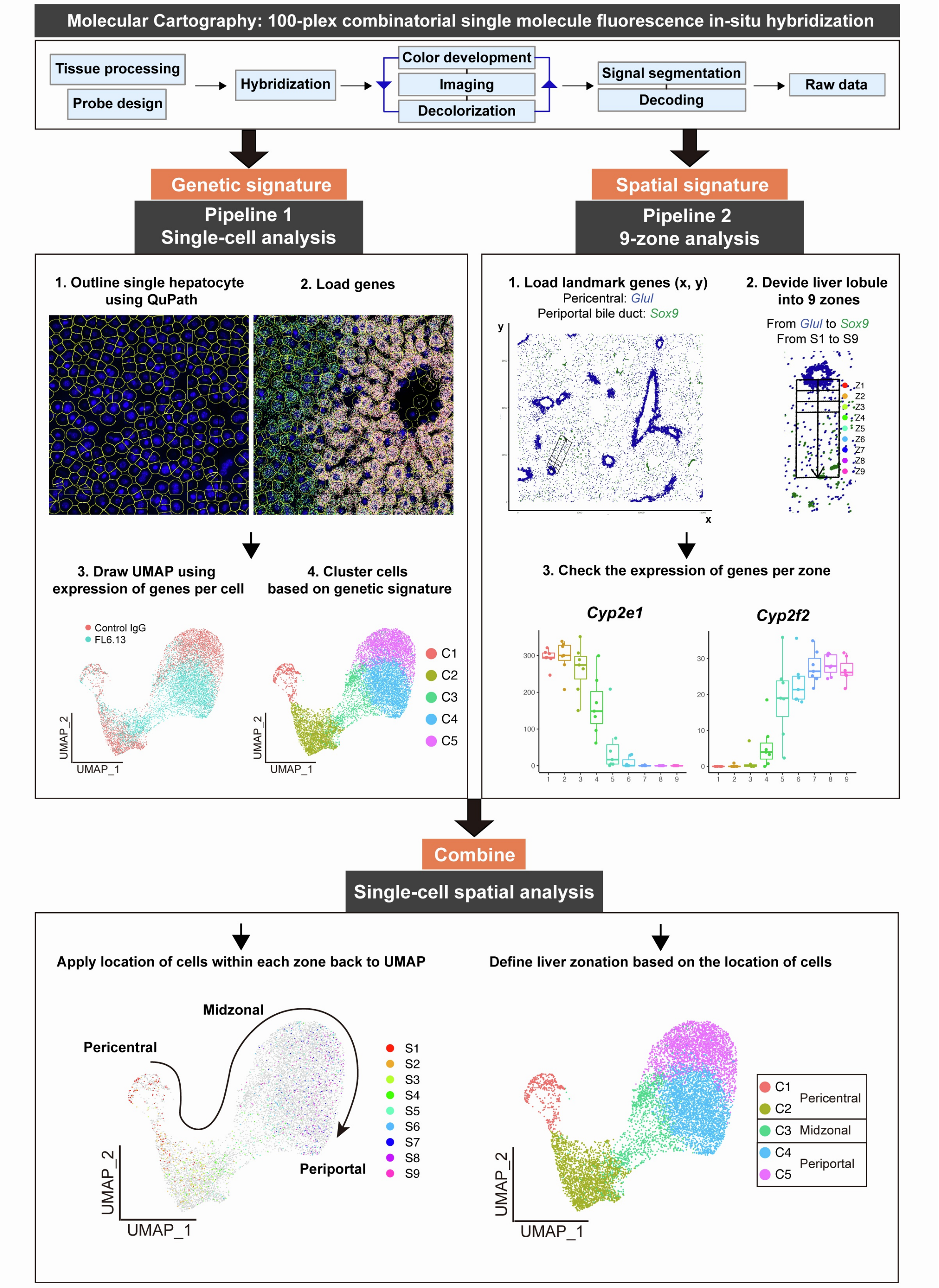

Fig. S13: Workflow of single-cell transcriptomics analysis of Molecular Cartography data of FL6.13-treated mice. (Related to Figure 6 and STAR Methods)

See STAR Methods for details.

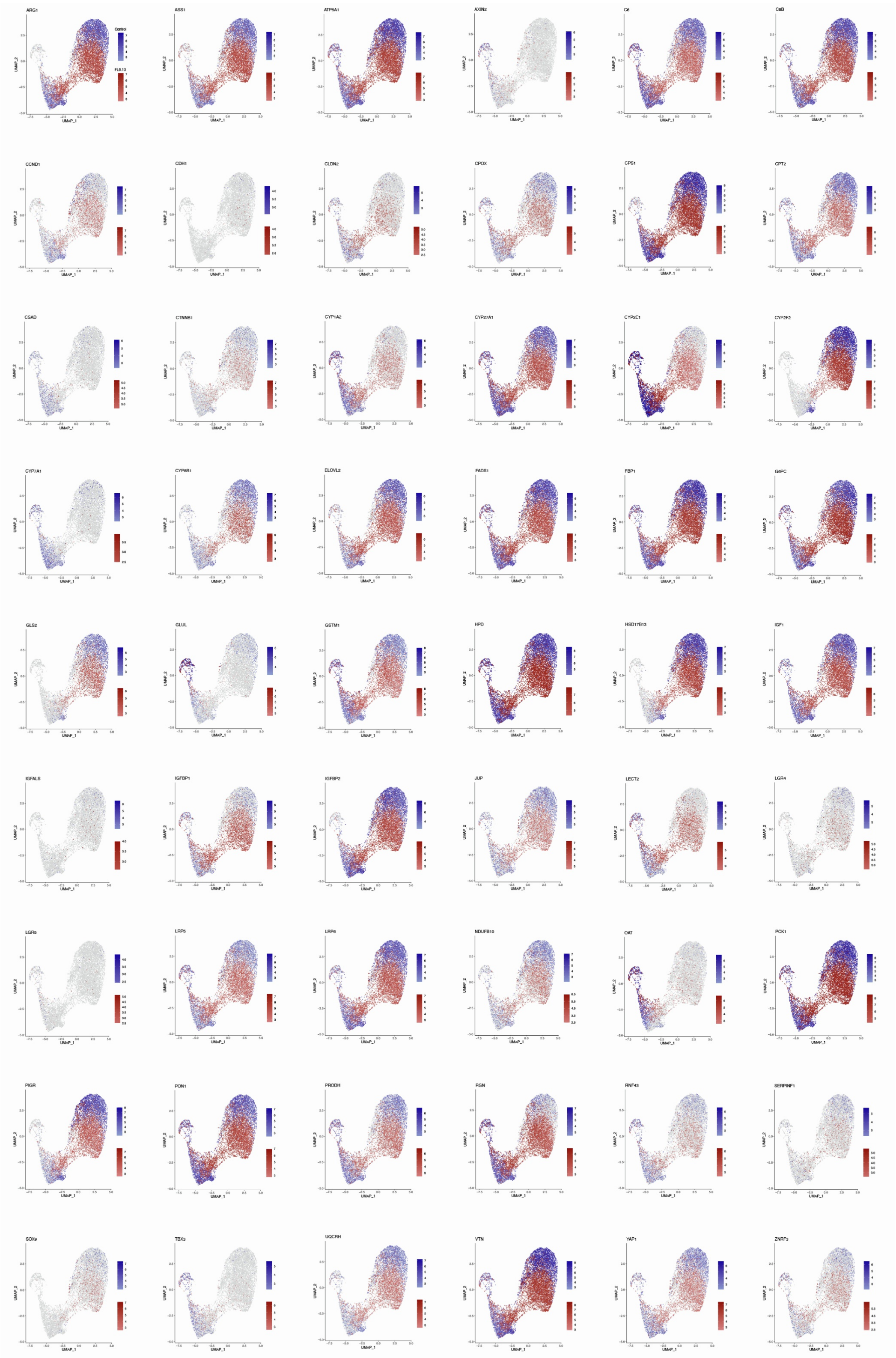

Fig. S14: Feature plots showing expression of genes in control and FL6.13-treated animal. (Related to Figure 6)
Blue: Control IgG; Red: FL6.13 treatment

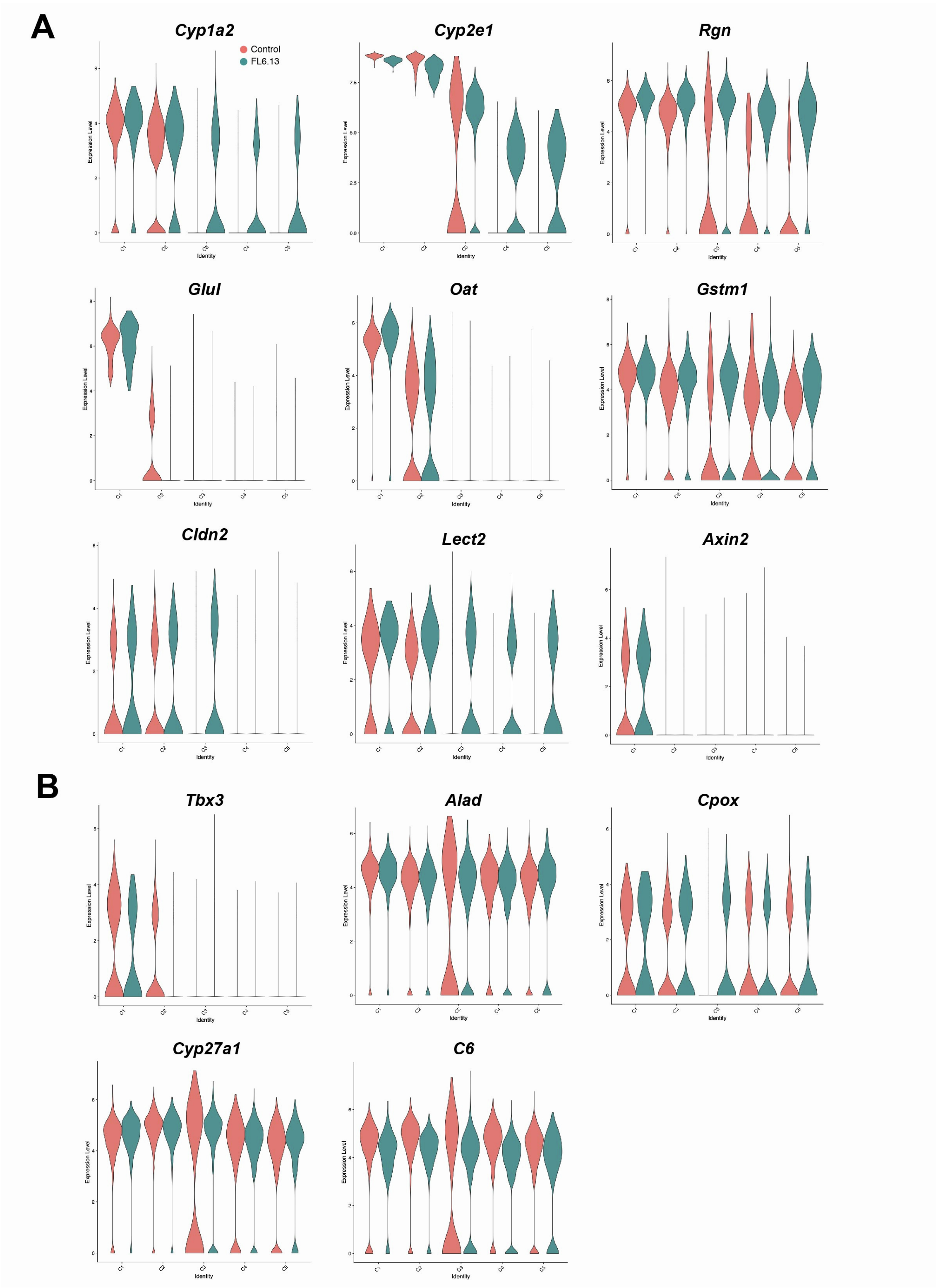

Fig. S15: Expression level of pericentral zonated genes in control and FL6.13-treated liver. (Related to Figure 6)

(A) Violin plots showing expanded expression of Wnt target genes after FL6.13 treatment.

(B) Violin plots showing genes that were not increased after FL6.13 treatment.

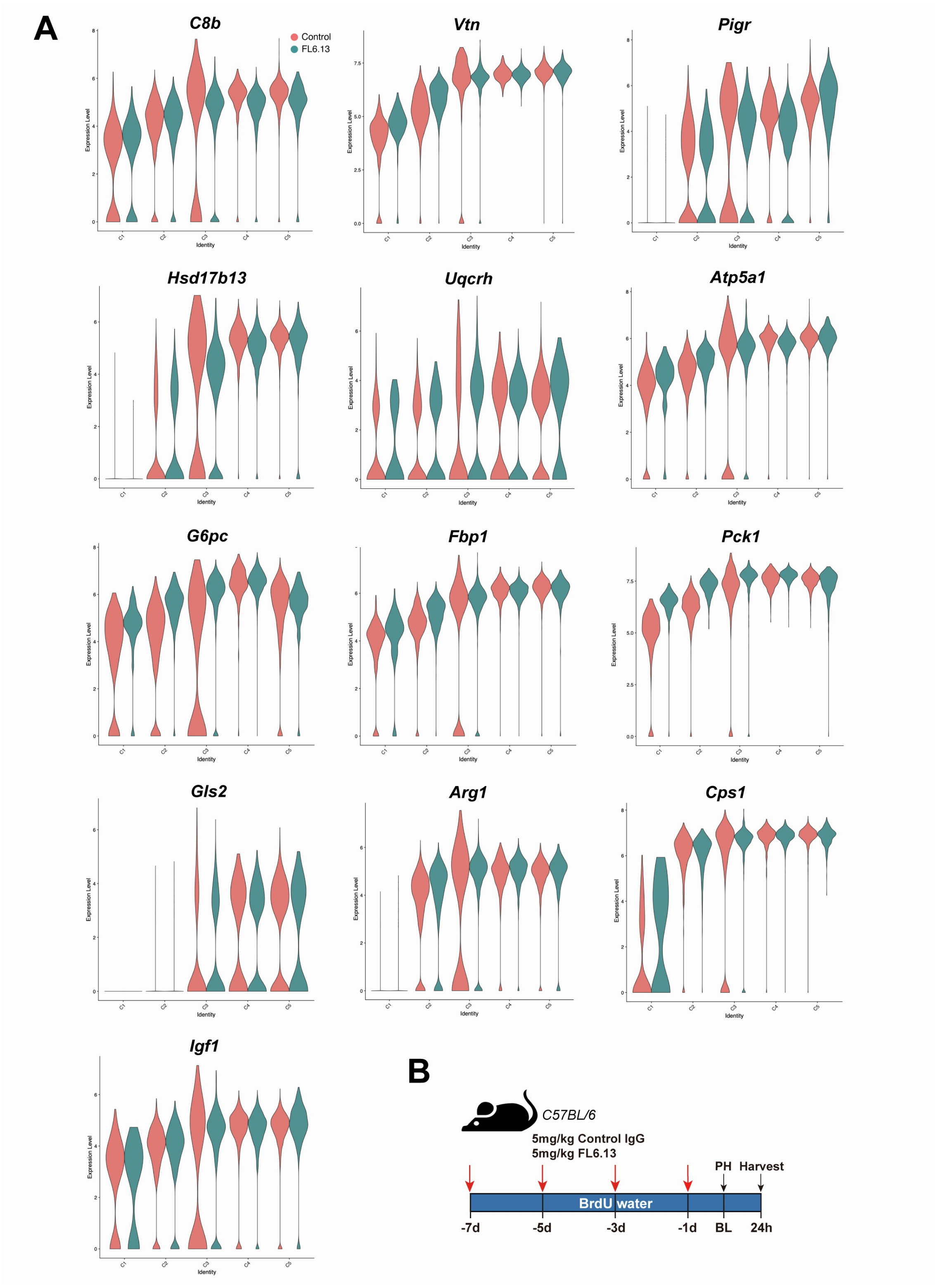

Fig. S16: Expression level of periportal zonated genes in control and FL6.13-treated liver. (Related to Figure 6)

(A) Violin plots showing that expression of periportal genes was not decreased after FL6.13 treatment.

(B) Study design of control IgG and FL6.13 treatment for regeneration study.

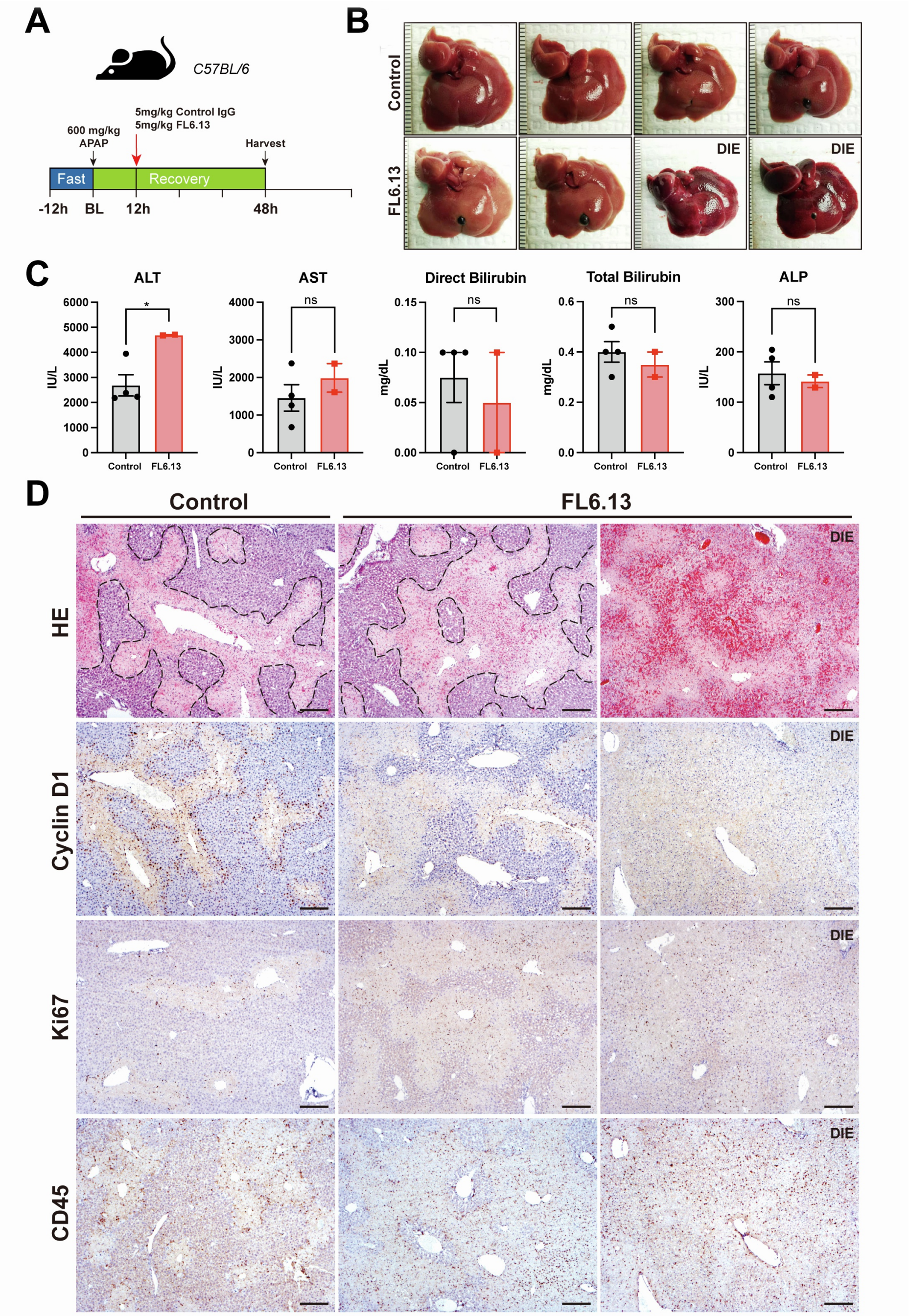

Fig. S17: Early treatment with FL6.13 worsens liver injury after acetaminophen overdose. (Related to Figure 7)

(A) Study design showing dosing schedule of pan-FZD agonist FL6.13 or isotype control IgG administration. Single dose of 5 mg/kg control IgG or FL6.13 was i.p. administrated to mice at 12 hours post 600 mg/kg i.p. APAP injection. Mice were sacrificed at 48 hours for analysis.

(B) Gross images of livers showing necrosis and congestion in control IgG and FL6.13-treated animals at 48 hours.

(C) Serum levels of ALT, AST, direct bilirubin, total bilirubin, and ALP at 48 hours showing increased liver injury when FL6.13 was given at 12 hours. (The bars represent means ± SEM. ns = not significant, *$P < 0.05$. n = 4, 2 mice)

(D) Representative IHC showing necrotic areas by HE, cell proliferation by Cyclin D1 and Ki67, and immune cell infiltration by CD45.

Supplementary Tables

Table S1: Sequence of genotyping primers (related to STAR Methods)

**Primer name**	**Sequence (5’-3’)**
Lyve1-Cre-F_wt	TGCCACCTGAAGTCTCTCCT
Lyve1-Cre-F_mutant	GAGGATGGGGACTGAAACTG
Lyve1-Cre-R	TGAGCCACAGAAGGGTTAGG
ROSA-EYFP-F_wt	GGAGCGGGAGAAATGGATATG
ROSA-EYFP-F_mutant	AAGACCGCGAAGAGTTTGTC
ROSA-EYFP-R	AAAGTCGCTCTGAGTTGTTAT
Wnt9b-F	GCAGAATCTGGAGAACTTGGC
Wnt9b-R	GTGAGAAGGAAGATGGTGAGC
Wnt2-F	CCCAGCAGGTGCTAAGAGG
Wnt2-R	CAATGGCACGCATCACATCT

Table S2: Size of PCR products (related to STAR Methods)

**Genotype**	**WT (bp)**	**Mutant (bp)**
Lyve1-Cre	425	210
Rosa-EYFP	600	324
Wnt9b	219	300
Wnt2	540	592

Table S3: Probe list for Molecular Cartography^{TM} (related to STAR Methods)

Item	Cat. No	Species	Design Target ID	Gene Name
1	P0D1P	Mus musculus	ENSMUSG00000006932	Ctnnb1
2	P0DCL	Mus musculus	ENSMUSG00000015957	Wnt11
3	P0ECZ	Mus musculus	ENSMUSG00000002588	Pon1
4	P1DC0	Mus musculus	ENSMUSG00000029671	Wnt16
5	P2D1R	Mus musculus	ENSMUSG00000024913	Lrp5
6	P2DC1	Mus musculus	ENSMUST00000054294	Fzd1
7	P2Y49	Mus musculus	ENSMUSG00000000303	Cdh1
8	P3DC2	Mus musculus	ENSMUSG00000022297	Fzd6
9	P3EC1	Mus musculus	ENSMUST00000102840	Ass1
10	P3F4T	Mus musculus	ENSMUSG00000004730	Adgre1
11	P4DC3	Mus musculus	ENSMUST00000114246	Fzd7
12	P4EC2	Mus musculus	ENSMUST00000020161	Arg1
13	P5DC4	Mus musculus	ENSMUST00000041080	Fzd8
14	P5EC3	Mus musculus	ENSMUST00000045602	Ndufb10
15	P638C	Mus musculus	ENSMUST00000092163	Lyz2
16	P6787	Mus musculus	ENSMUST00000019469	G6pc
17	P6C1X	Mus musculus	ENSMUSG00000020053	Igf1
18	P6DC5	Mus musculus	ENSMUST00000032322	Lrp6
19	P7DC6	Mus musculus	ENSMUSG00000050199	Lgr4
20	P7EC5	Mus musculus	ENSMUST00000092888	Fbp1
21	P8DC7	Mus musculus	ENSMUSG00000034177	Rnf43
22	P8EC6	Mus musculus	ENSMUST00000050714	Igfals
23	P948E	Mus musculus	ENSMUST00000003100	Cyp2f2
24	P9DC8	Mus musculus	ENSMUSG00000041961	Znrf3
25	P9M7Y	Mus musculus	ENSMUST00000092623	Rspo3
26	PAN7Y	Mus musculus	ENSMUST00000044776	Gls2
27	PCN7Z	Mus musculus	ENSMUST00000029017	Pck1
28	PDECA	Mus musculus	ENSMUSG00000021364	Elovl2
29	PEECC	Mus musculus	ENSMUSG00000010663	Fads1
30	PEV7V	Mus musculus	ENSMUST00000029905	Cyp7a1
31	PFE89	Mus musculus	ENSMUST00000117102	Fzd10
32	PFG43	Mus musculus	ENSMUSG00000026395	Ptprc
33	PFVCL	Mus musculus	ENSMUST00000057893	Fzd2
34	PGC15	Mus musculus	ENSMUSG00000022382	Wnt7b
35	PGECE	Mus musculus	ENSMUSG00000029656	C8b
36	PGVC0	Mus musculus	ENSMUSG00000001552	Jup
37	PHE8C	Mus musculus	ENSMUST00000131309	Fzd3
38	PHVC1	Mus musculus	ENSMUSG00000024182	Axin1
39	PJDCH	Mus musculus	ENSMUST00000024954	Epas1
40	PJE8D	Mus musculus	ENSMUST00000058755	Fzd4
41	PJM75	Mus musculus	ENSMUST00000010941	Wnt2
42	PJR71	Mus musculus	ENSMUST00000093962	Ccnd1

43	PJVC2	Mus musculus	ENSMUSG00000005871	Apc
44	PKCCK	Mus musculus	ENSMUST00000023734	Wnt1
45	PKDCJ	Mus musculus	ENSMUSG00000015522	Arnt
46	PKE8E	Mus musculus	ENSMUSG00000045005	Fzd5
47	PKM76	Mus musculus	ENSMUST00000018630	Wnt9b
48	PKT6L	Mus musculus	ENSMUST00000000579	Sox9
49	PKVC3	Mus musculus	ENSMUSG00000031169	Porcn
50	PMA5F	Mus musculus	ENSMUSG00000028393	Alad
51	PMCCM	Mus musculus	ENSMUST00000029429	Wnt2b
52	PMDCK	Mus musculus	ENSMUST00000060077	Cpox
53	PMECJ	Mus musculus	ENSMUST00000017488	Vtn
54	PMP1L	Mus musculus	ENSMUSG00000039323	Igfbp2
55	PMVC4	Mus musculus	ENSMUSG00000028173	Wls
56	PNCCN	Mus musculus	ENSMUST00000000127	Wnt3
57	PNDCM	Mus musculus	ENSMUSG00000022181	C6
58	PNVC5	Mus musculus	ENSMUSG00000018569	Cldn7
59	PPCCP	Mus musculus	ENSMUST00000010044	Wnt3a
60	PPDCN	Mus musculus	ENSMUST00000054889	Cldn2
61	PPHCH	Mus musculus	ENSMUSG00000034528	Hsd17b13
62	PPVC6	Mus musculus	ENSMUSG00000021539	Lect2
63	PQCCQ	Mus musculus	ENSMUST00000045747	Wnt4
64	PQE8J	Mus musculus	ENSMUST00000062572	Fzd9
65	PQVC7	Mus musculus	ENSMUSG00000003526	Prodh
66	PR47V	Mus musculus	ENSMUSG00000053110	Yap1
67	PR56S	Mus musculus	ENSMUSG00000018604	Tbx3
68	PR81H	Mus musculus	ENSMUSG00000020717	Pecam1
69	PRCCR	Mus musculus	ENSMUSG00000021994	Wnt5a
70	PRVC8	Mus musculus	ENSMUST00000034860	Cyp1a2
71	PS68V	Mus musculus	ENSMUST00000027144	Cps1
72	PSCCS	Mus musculus	ENSMUST00000006716	Wnt6
73	PSDCR	Mus musculus	ENSMUST00000031398	Hpd
74	PSVC9	Mus musculus	ENSMUST00000023832	Rgn
75	PT86R	Mus musculus	ENSMUSG00000000753	Serpinf1
76	PTCCT	Mus musculus	ENSMUST00000032180	Wnt7a
77	PTDCS	Mus musculus	ENSMUST00000084500	Oat
78	PTF9N	Mus musculus	ENSMUST00000030687	Rspo1
79	PTVCA	Mus musculus	ENSMUST00000078676	Uqcrh
80	PV85R	Mus musculus	ENSMUSG00000025428	Atp5a1
81	PVCCV	Mus musculus	ENSMUST00000012426	Wnt8a
82	PVF4H	Mus musculus	ENSMUSG00000025479	Cyp2e1
83	PWCCW	Mus musculus	ENSMUST00000041163	Wnt8b
84	PWDCV	Mus musculus	ENSMUSG00000019838	Slc16a10
85	PWF4J	Mus musculus	ENSMUSG00000020140	Lgr5
86	PWVCD	Mus musculus	ENSMUSG00000028607	Cpt2

87	PXCCX	Mus musculus	ENSMUSG00000000126	Wnt9a
88	PXDCW	Mus musculus	ENSMUST00000062474	Cyp8b1
89	PXF4K	Mus musculus	ENSMUSG00000000142	Axin2
90	PXV8A	Mus musculus	ENSMUSG00000023044	Csad
91	PXVCE	Mus musculus	ENSMUST00000027675	Pigr
92	PXWCD	Mus musculus	ENSMUSG00000058135	Gstm1
93	PYCCY	Mus musculus	ENSMUST00000006718	Wnt10a
94	PYDCX	Mus musculus	ENSMUST00000027356	Cyp27a1
95	PYWCE	Mus musculus	ENSMUST00000020704	Igfbp1
96	PZ78L	Mus musculus	ENSMUST00000029632	Lrat
97	PZ81R	Mus musculus	ENSMUSG00000026473	Glul
98	PZ85W	Mus musculus	ENSMUSG00000021109	Hif1a
99	PZCAY	Mus musculus	ENSMUSG00000030170	Wnt5b
100	PZCCZ	Mus musculus	ENSMUSG00000022996	Wnt10b

Table S4: Sequence of qPCR primers (related to STAR Methods)

Gene	Forward	Reverse
Rn18s	GTAACCCGTTGAACCCCATT	CCATCCAATCGGTAGTAGCG
Glul	CTCGCTCTCCTGACCTGTTC	TTCAAGTGGGAACTTGCTGA
Cyp2e1	AATGGACCTACCTGGAAGGAC	CCTCTGGATCCGGCTCTCATT
Axin2	TGACTCTCCTTCCAGATCCCA	TGCCCACACTAGGCTGACA
Lect2	CCCACAACAATCCTCATTTCA	GTTAGCCCATGGTCCTGCTA
Oat	CCGACCAGTTATGATGGCTTTGG	CTCCACCATGAAGGCAGCAACA
Rgn	GTATGGGAGGAAGCGTCACAGT	CAATGGTGGCAACATAGCCTCC
Cldn2	GCAAACAGGCTCCGAAGATACT	GAGATGATGCCCAAGTACAGAG
Gstm1	ATACTGGGATACTGGAACGTCC	AGTCAGGGTTGTAACAGAGCAT
Lgr5	CCTACTCGAAGACTTACCCAGT	GCATTGGGGTGAATGATAGCA
Tbx3	ACTCGGGGTCGGAACTGAA	GGAGGGGGCGATTTTGTTTTT
G6pc	CAGTGGTCGGAGACTGGTTC	TATAGGCACGGAGCTGTTGC
Pck1	TGTCTTCACTGAGGTGCCAG	CTGGATGAAGTTTGATGCCC
Arg1	ACAAGACAGGGCTCCTTTCAG	TGAGTTCCGAAGCAAGCCAA
Cps	AGGATGTCAAGGTGTTTGGC	GCTTAACTAGCAGGCGGATG
Ass1	ACACCTCCTGCATCCTCGT	GCTCACATCCTCAATGAACACCT
Atp5a1	TGGTGAAGAGACTGACGGATGC	TCAAAGCGTGCTTGCCGTTGTC
Igf1	TCATGTCGTCTTCACACCTCTTCT	CCACACACGAACTGAAGAGCAT
C8b	ACTGTCAACGGGAGATGGAGCA	GTTGGTGTCCAGGATGTAGTGG
Cyp8b1	AGTACACATGGACCCCGACATC	GGGTGCCATCCGGGTTGAG
Hpd	CGCTCCATTGTGGTGACCAACT	TCCGTCTTGAGAGCGATGTGCT
Ccnd1	TTTCTTTCCAGAGTCATCAAGTGT	TGACTCCAGAAGGGCTTCAA
