# Supplementary material for: Single-cell spatial transcriptomics reveals a dynamic control of metabolic zonation and liver regeneration by endothelial cell Wnt2 and Wnt9b
Source: Cell Rep Med. 2022 Oct 10;3(10):100754. doi: 10.1016/j.xcrm.2022.100754 (PMC9588996; doi:10.1016/j.xcrm.2022.100754)
Supplement: Document S2. Article plus supplemental information [file mmc2.pdf]

Cell Reports
Medicine

Article

Single-cell spatial transcriptomics reveals a dynamic control of metabolic zonation and liver regeneration by endothelial cell Wnt2 and Wnt9b

Graphical abstract

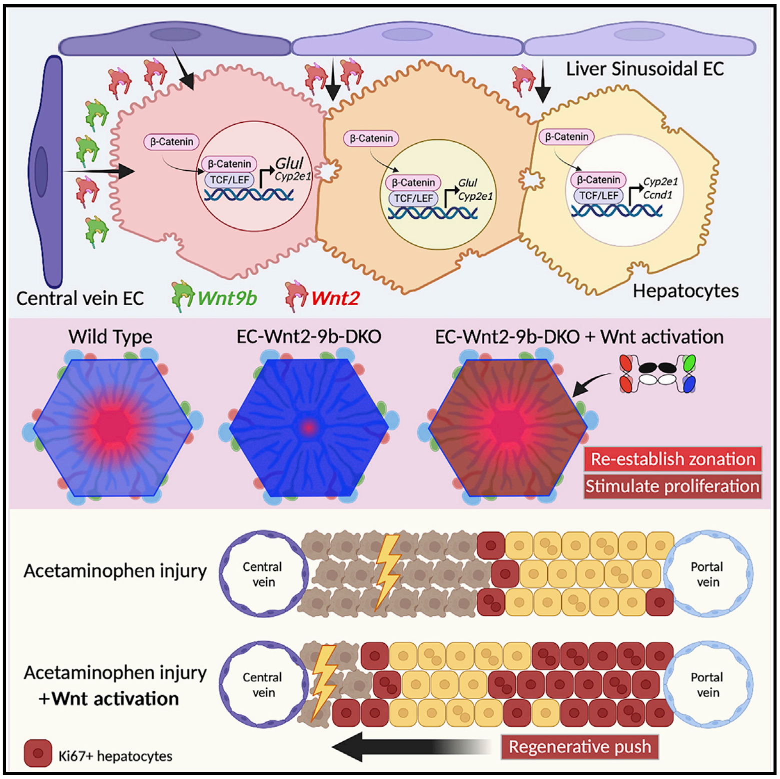

Highlights

- Conserved expression of Wnt2 and Wnt9b in liver endothelial cells is identified
- Wnt2 and Wnt9b deletion from endothelial cells affects gene expression in zone 3
- Endothelial Wnt2 and Wnt9b deletion affects liver regeneration after hepatectomy
- Late treatment with Wnt agonist rescues regeneration after acetaminophen overdose

Authors

Shikai Hu, Silvia Liu, Yu Bian, ...,
Sachdev S. Sidhu, Stephane Angers,
Satdarshan P. Monga

Correspondence

smonga@pitt.edu

In brief

Hu et al. show conserved expression of Wnt2 and Wnt9b in liver endothelial cells whose deletion led to abrogation of expression of zone 3 genes, appearance of zone 1 genes in this area, and blunted liver regeneration. Wnt agonist stimulated regeneration in acute liver injury model.

Hu et al., 2022, Cell Reports Medicine *3*, 100754
October 18, 2022 © 2022 The Author(s).
https://doi.org/10.1016/j.xcrm.2022.100754

CellPress

Cell Reports Medicine

CellPress
OPEN ACCESS

Article

Single-cell spatial transcriptomics reveals a dynamic control of metabolic zonation and liver regeneration by endothelial cell Wnt2 and Wnt9b

Shikai Hu,[1,2] Silvia Liu,[2,5] Yu Bian,[2] Minakshi Poddar,[2] Sucha Singh,[2] Catherine Cao,[2] Jackson McGaughey,[2] Aaron Bell,[2,5] Levi L. Blazer,[3] Jarret J. Adams,[3] Sachdev S. Sidhu,[3] Stephane Angers,[3,4] and Satdarshan P. Monga[2,5,6,7,*]

[1]School of Medicine, Tsinghua University, Beijing, China
[2]Division of Experimental Pathology, Department of Pathology, University of Pittsburgh School of Medicine, Pittsburgh, PA, USA
[3]Donnelly Centre, University of Toronto, Toronto, ON, Canada
[4]Leslie Dan Faculty of Pharmacy, University of Toronto, Toronto, ON, Canada
[5]Pittsburgh Liver Research Center, University of Pittsburgh Medical Center and University of Pittsburgh School of Medicine, Pittsburgh, PA, USA
[6]Division of Gastroenterology, Hepatology and Nutrition, Department of Medicine, University of Pittsburgh School of Medicine, Pittsburgh, PA, USA
[7]Lead contact
*Correspondence: smonga@pitt.edu
https://doi.org/10.1016/j.xcrm.2022.100754

SUMMARY

The conclusive identity of Wnts regulating liver zonation (LZ) and regeneration (LR) remains unclear despite an undisputed role of β-catenin. Using single-cell analysis, we identified a conserved Wnt2 and Wnt9b expression in endothelial cells (ECs) in zone 3. EC-elimination of Wnt2 and Wnt9b led to both loss of β-catenin targets in zone 3, and re-appearance of zone 1 genes in zone 3, unraveling dynamicity in the LZ process. Impaired LR observed in the knockouts phenocopied models of defective hepatic Wnt signaling. Administration of a tetravalent antibody to activate Wnt signaling rescued LZ and LR in the knockouts and induced zone 3 gene expression and LR in controls. Administration of the agonist also promoted LR in acetaminophen overdose acute liver failure (ALF) fulfilling an unmet clinical need. Overall, we report an unequivocal role of EC-Wnt2 and Wnt9b in LZ and LR and show the role of Wnt activators as regenerative therapy for ALF.

INTRODUCTION

The Wnt-β-catenin signaling pathway plays fundamental roles in tissue development, homeostasis, repair, regeneration, and tumorigenesis.[1,2] β-Catenin transcriptional activity is controlled by Wnt proteins. Once secreted with the help of the cargo protein Wntless (Wls), Wnt proteins bind to cell surface receptor Frizzled (FZD) and co-receptors low-density lipoprotein receptor-related protein 5/6 (LRP5/6). This leads to inactivation of the destruction complex composed of APC-GSK3β-Axin and allows β-catenin nuclear translocation and target gene expression.[2]

The Wnt-β-catenin signaling pathway and the RSPO-LGR4/5-ZNRF3/RNF43 axis are well-known regulators of pericentral gene expression and in turn of metabolic liver zonation (LZ) and also contribute to liver regeneration (LR) after partial hepatectomy.[3–6] LZ is a function of the hepatocytes exhibiting differential gene expression based on their location along the portal-central axis of a hepatic lobule.[7] Such heterogeneity allows hepatocytes located within each zone to perform specific metabolic, synthetic, and xenobiotic functions, improving efficiency through division of labor. LZ also allows compartmentalization of certain injuries such that the remaining cells could proliferate to restore liver mass and function.[8] LR after partial hepatectomy (PH) is also a distinctive feature of an adult liver, which is evolutionarily conserved, and allows the remnant liver to grow after surgical resection.[9,10] Mice with liver-specific knockout of β-catenin (β-catenin-LKO) or liver-specific double-knockout of LRP5 and LRP6 (LRP5-6-LDKO) have disrupted pericentral LZ as seen by loss of pericentral expression of glutamine synthetase, CYP2E1, and CYP1A2; and delayed LR due to impaired cyclin D1 expression.[11–14] Hepatic endothelial cells (ECs) are the main source of Wnt ligands in an adult murine liver.[15–17] Like β-catenin-LKO and LRP5-6-LDKO, mice with EC Wls deletion (EC-Wls-KO) lack pericentral LZ and show delayed LR. While 19 Wnt ligands are expressed in various cell types of the liver, their distinct pathophysiological roles *in vivo* are less well understood.[18] In murine livers, *Wnt2* and *Wnt9b* are present at the central venous ECs and hepatic sinusoidal ECs near the central vein.[19] PH induces a 45-fold upregulation in *Wnt2* and 18-fold upregulation *Wnt9b* expression in ECs at 12 h.[17] Upregulation of *Wnt2* and *Wnt9b* is also apparent in other hepatic regeneration settings.[20,21] While these correlative studies suggest roles of these two Wnts in the liver, conclusive proof requires direct genetic evidence.

While mechanisms of LR have been studied for several decades, there are no targeted therapies available to stimulate

© 2022 The Author(s).
This is an open access article under the CC BY license (http://creativecommons.org/licenses/by/4.0/).

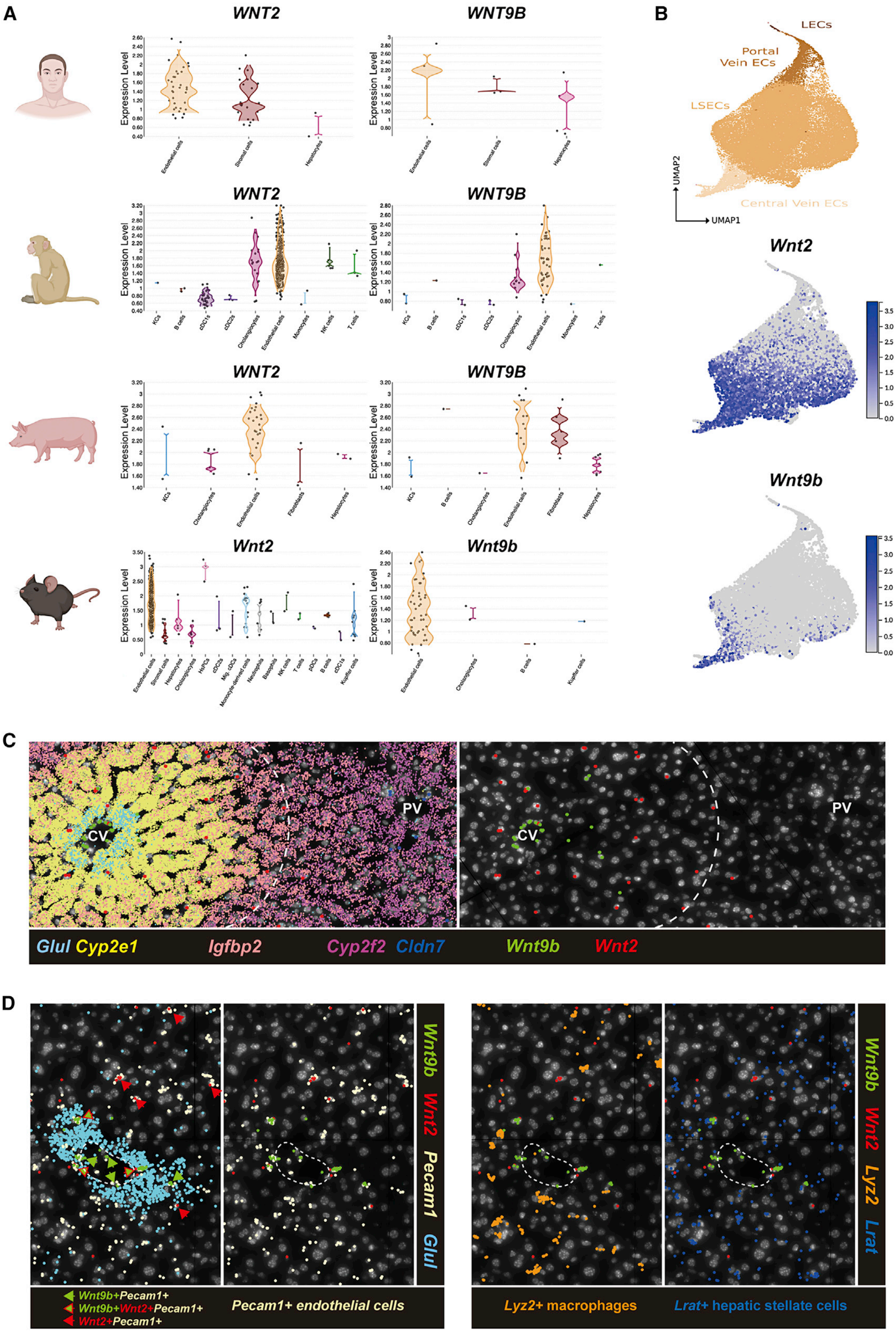

(legend on next page)

LR in acute or chronic liver injuries. Since the Wnt-β-catenin pathway contributes to both LZ and LR, it is a promising candidate pathway for drug development for regenerative medicine.[5,22,23] Recently F^{P+P}-$L6^{1+3}$ (abbreviated FL6.13), a water-soluble tailored tetravalent antibody consisting of two pan-FZD paratopes and two anti-LRP6 paratopes was developed through rational design.[24] FL6.13 promotes FZD-LRP6 clustering to stabilize receptor conformations compatible with robust β-catenin activation at nanomolar concentrations in both murine and human cell lines, organoids, and *in vivo*.

Here, we analyzed liver cell-specific expression of Wnt gene transcripts across different species using an existing single-cell RNA (scRNA) sequencing database that allowed us to identify Wnt2 and Wnt9b in ECs.[25] We generated Wnt2 and Wnt9b double floxed mice to breed to lymphatic vessel endothelial hyaluronan receptor 1 (Lyve1)-Cre mice and generated EC-Wnt2-KO, EC-Wnt9b-KO, and EC-Wnt2-9b-DKO mice. The EC-Wnt2-9b-DKO mice exhibited perturbed metabolic LZ as revealed using 100-gene single-cell spatial transcriptomics by Molecular Cartography, which was rescued by FL6.13. The EC-Wnt2-9b-DKO mice showed delayed LR after PH, which was rescued by FL6.13. FL6.13 also induced β-catenin-dependent pericentral gene expression and cell proliferation program, and successfully promoted LR in murine model of surgical resection and rescued delayed acetaminophen (APAP) overdose-induced liver injury, thus fulfilling a major unmet clinical need.

RESULTS

Endothelial cell expression of *Wnt2* and *Wnt9b* is evolutionarily conserved

Since the Wnt-β-catenin signaling pathway is important for liver function, we queried expression of various Wnt genes in different liver cells through a searchable scRNA sequencing database from cells within livers of various species.[25] In the human dataset, 18 out of 19 WNTs were detected in various hepatic cell types (Figure S1). ECs were the predominant source of *WNT2*, *WNT2B*, and *WNT9B*, although the signal of *WNT9B* was weak (Figures 1A and S1). In monkey, *WNT2*, *WNT2B*, *WNT3*, *WNT9A*, and *WNT9B* were highly expressed among 16 detected WNTs (Figures 1A and S2). ECs expressed high levels of *WNT2* and *WNT9B*, while *WNT2B* was mainly expressed by Kupffer cells. In the pig, 9 WNTs were detected (Figures 1A and S3). ECs in porcine liver also expressed very high levels of *WNT2* and *WNT9B*. *WNT2B* was expressed at a very low level. In mice, 15 Wnts were detected, of which *Wnt2* and *Wnt9b* were highest in ECs (Figures 1A and S4). Even in mice with NAFLD, *Wnt2* and *Wnt9b* were still predominantly expressed in ECs, suggesting preservation of their expression even in pathological states (Figure S5). Altogether, as could be seen from analysis in multiple species (Figure 1A), ECs are uniformly the predominant source of *Wnt2* and *Wnt9b* and only EC expression of *Wnt2* and *Wnt9b* appears to be evolutionarily conserved.

ECs are a heterogeneous population across the liver lobule.[26,27] By using Uniform Manifold Approximation and Projection (UMAP) for dimension reduction, we observed pericentrally zonated expression of *Wnt2* and *Wnt9b* in humans (Figure S6) and mice (Figure 1B). Zonal expression of *Wnt2* and *Wnt9b* was also confirmed in another murine single-nuclei RNA sequencing database (Figure S7). Consistently, *Wnt9b* was expressed mainly by central venous ECs, while *Wnt2* was expressed more broadly by both central venous ECs and sinusoidal ECs (Figures 1B and S6).

To further validate these observations, we applied Molecular Cartography (Resolve Biosciences), which allows 100-plex spatial mRNA analysis on wild-type C57BL/6 mouse liver. Genes encoding for various components of the Wnt pathway, and genes that are known to be zonated, were spatially resolved by Molecular Cartography (Table S3). We identified central-portal zonation of hepatocytes using location of zonated genes (pericentral: *Glul*, *Cyp2e1*; midzonal: *Igfbp2*; periportal: *Cyp2f2*) and also identified cholangiocytes through *Cldn7* expression (Figure 1C). Of the 19 Wnts, only *Wnt2* and *Wnt9b* were pericentrally zonated (Figure 1C). To confirm the cellular source of *Wnt2* and *Wnt9b*, we mapped gene expression of cell-specific markers, such as *Pecam1* for ECs, *Lyz2* for macrophages, and *Lrat* for hepatic stellate cells (HSCs). *Wnt2* and *Wnt9b* predominantly colocalized with *Pecam1*, while some overlap was evident with *Lyz2* and *Lrat*, confirming ECs to be the major *Wnt2*- and *Wnt9b*-expressing cells in zone 3 of the murine liver (Figures 1D and S8).

Altogether, these data suggest spatially confined expression of *Wnt2* and *Wnt9b* in ECs, which, along with *Rspo3* from ECs and HSCs, might be instructing pericentral Wnt-β-catenin activity to contribute to metabolic LZ.[28,29]

Hepatic EC deletion of *Wnt2* and *Wnt9b* in mice

Wnt2$^{flox/flox}$ mice with *loxP* sites flanking exon 2 of the murine *Wnt2* gene were generated using CRISPR-Cas9 as discussed in STAR Methods (Figure S9A). *Wnt9b*$^{flox/flox}$ mice and Lyve1-Cre mice were obtained from Jackson laboratories.[17,30] Rosa-stop$^{flox/flox}$-EYFP mice were bred to these strains to fate-trace the expression and activity of Cre-recombinase transgene. After strategic breeding (discussed in STAR Methods), we successfully generated EC-Wnt2-9b-DKO mice that were identified by the simultaneous presence of Cre, floxed *Wnt2*, floxed *Wnt9b*, and Rosa-stop alleles in the genomic DNA PCR (Figure S9B).

Figure 1. Endothelial cell expression of *Wnt2* and *Wnt9b* is evolutionarily conserved

(A) Violin plots showing expression levels of *WNT2* and *WNT9B* in human, monkey (male Cynomolgus macaques), and pig (female piglets) livers, and *Wnt2* and *Wnt9b* in mice (C57BL/6) livers among different hepatic cell types, with highest expression evident in endothelial cells across species. Cartoons were created with BioRender.com.

(B) Feature plots showing expression of *Wnt2* and *Wnt9b* among all hepatic ECs in mice. While *Wnt9b* is almost exclusively expressed in central vein endothelial cells, *Wnt2* is expressed more widely in both central vein endothelial cells as well as in liver sinusoidal endothelial cells (LSECs) toward zones 2 and 3.

(C) Molecular Cartography showing expression in hepatocytes of *Glul and Cyp2e1* (zone 3), *Igfbp2* (zone 2), *Cyp2f2* (zone 1), and *Cldn7* (cholangiocytes), along with *Wnt2* and *Wnt9b*, which are pericentrally zonated. CV, central vein; PV, portal vein.

(D) Molecular Cartography of *Wnt2* and *Wnt9b* along with markers of specific cell types showing *Pecam1*+ ECs, but not *Lyz2*+ macrophages or *Lrat*+ hepatic stellate cells are the main source of *Wnt2* and *Wnt9b*.

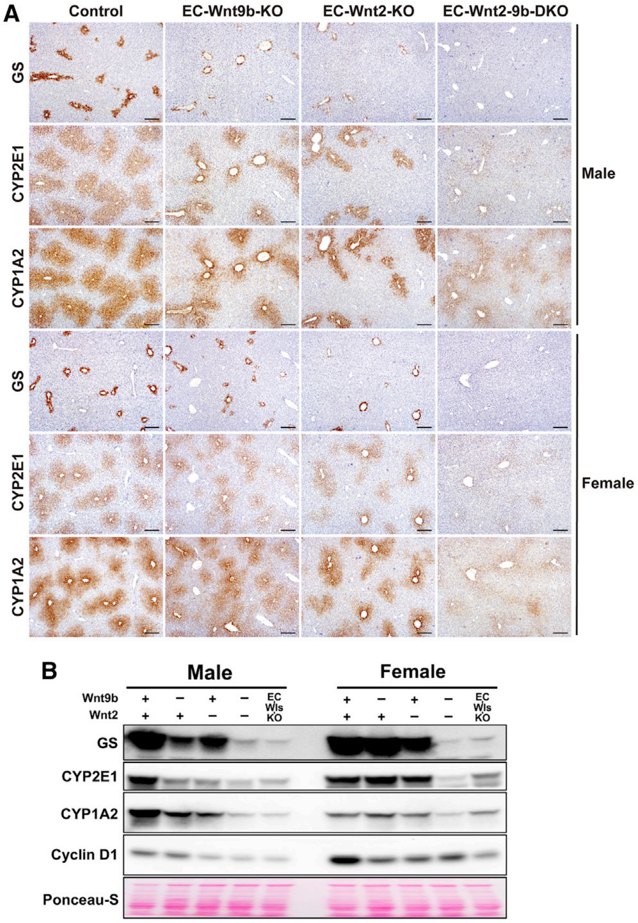

Figure 2. EC-derived Wnt2 and Wnt9b control expression of key β-catenin target genes in the baseline liver

(A) Immunohistochemistry showing zonation of representative pericentral enzyme GS, CYP2E1, and CYP1A2 in representative male and female mice from age- and sex-matched littermate controls (Control), EC-Wnt9b-KO, EC-Wnt2-KO, and EC-Wnt2-9b-DKO. Scale bars, 200 μm.

(B) Representative western blot (WB) from whole liver lysate of various genotypes showing total levels of β-catenin target genes to be modestly decreased in single EC knockouts of *Wnt2* and *Wnt9b*, but more profoundly decreased in double KOs (DKOs).

While *Lyve1* is mainly expressed by midzonal hepatic sinusoidal ECs in an adult murine liver, Lyve1-Cre recombines floxed alleles in both hepatic sinusoidal ECs and vascular ECs, likely due to expression of *Lyve1* sometime during development in all ECs.[16,17] To reconfirm, GFP expression in livers from EC-Wnt2-9b-DKO was seen in both hepatic sinusoidal and central venous ECs by immunohistochemistry (IHC) and immunofluorescence (Figures S9C and S9D).

To examine the impact of *Wnt2*, *Wnt9b*, or combined deletion from hepatic ECs, we assessed serum of mice from each genotype for transaminases and examined hepatic histology. Liver transaminases and histology from all genotypes were insignificantly different from the controls (Figure S9E and not shown). Liver weight to body weight ratio (LW/BW) was around 22% and significantly lower in the EC-Wnt2-KO and EC-Wnt9b-KO mice when compared with the controls and was even lower in EC-Wnt2-9b-DKO mice, similar to previously reported hepatic genetic KO of various components of the Wnt-β-catenin pathway (Figure S9F).[13,14,17]

EC-derived Wnt2 and Wnt9b control expression of key β-catenin target genes in baseline liver

Since loss of β-catenin or LRP5-6 from hepatocytes or Wls from ECs resulted in loss of Wnt-β-catenin target genes in pericentral zone, we next examined their expression in EC-Wnt2-KO, EC-Wnt9b-KO, and EC-Wnt2-9b-DKO mice. In males, by IHC and by western blot, single deletion of *Wnt2* or *Wnt9b* led to partial but consistent loss of pericentral expression of GS, CYP2E1, and CYP1A2 (Figures 2A and 2B). Minimal difference in their expression was evident in females (Figures 2A and 2B). Interestingly, both male and female EC-Wnt2-9b-DKO mice exhibited complete loss of pericentral GS expression and had minimal residual CYP2E1 and CYP1A2 (Figure 2A). Lack of CYP2E1 and CYP1A2 led to absence of APAP metabolism to NAPQI and hence EC-Wnt2-9b-DKO mice showed normal serum transaminases compared with controls, which showed 20,000 U/L at 24 h after 450 mg/kg of APAP treatment (not shown). In fact, EC-Wnt2-9b-DKO phenocopied β-catenin-LKO, LRP5-6-DLKO, and EC-Wls-KO mice, proving that, among the 19 Wnts, Wnt2 and Wnt9b are sufficient for maintaining baseline expression of key pericentral hepatocyte targets of the Wnt-β-catenin pathway and their dual deletion cannot be compensated by other mechanisms (Figure 2B).

Cyclin D1 is a β-catenin target gene that is mainly expressed in hepatocytes in the midzone of a quiescent liver. Like in various models of Wnt pathway disruption in the liver, cyclin D1 was decreased in both single and double KO mice in both males and females, but more profoundly in males and in DKOs (Figure 2B).[13,14,17]

Single-cell spatial transcriptomic profiling reveals a critical role of EC Wnt2 and Wnt9b in dynamic control of metabolic zonation

To evaluate zonation changes in-depth, we applied Molecular Cartography to study the single-cell spatial expression of 100 genes (Table S3). Two pipelines were used for the analysis of the control and EC-Wnt2-9b-DKO mice (Figure S10). First, to obtain the genetic signature at single-cell level, we used QuPath to outline single hepatocytes and obtained the expression of genes per cell. Comparable numbers of cells were obtained from control and DKO livers (Figure 3A). Next, using expression of 16 known zonated genes, a UMAP was generated, which identified six distinct hepatocyte populations (Figure S10). Clusters 1 and 2 represented hepatocytes with expression of pericentral genes, while clusters 4, 5, and 6 represented cells expressing periportal genes (Figure 3A). Intriguingly we observed a dramatic enrichment of cells in cluster 4, 5, and 6 at the expense of cells exhibiting pericentral genes in EC-Wnt2-9b-DKO, indicating an overall shift of gene expression from pericentral to periportal in these livers (Figure 3A).

Next, to obtain the spatial signature, we divided the liver lobule evenly into nine segments (pericentral to periportal: S1 to S9) using landmark genes (Figure S10). Gene expression density (gene

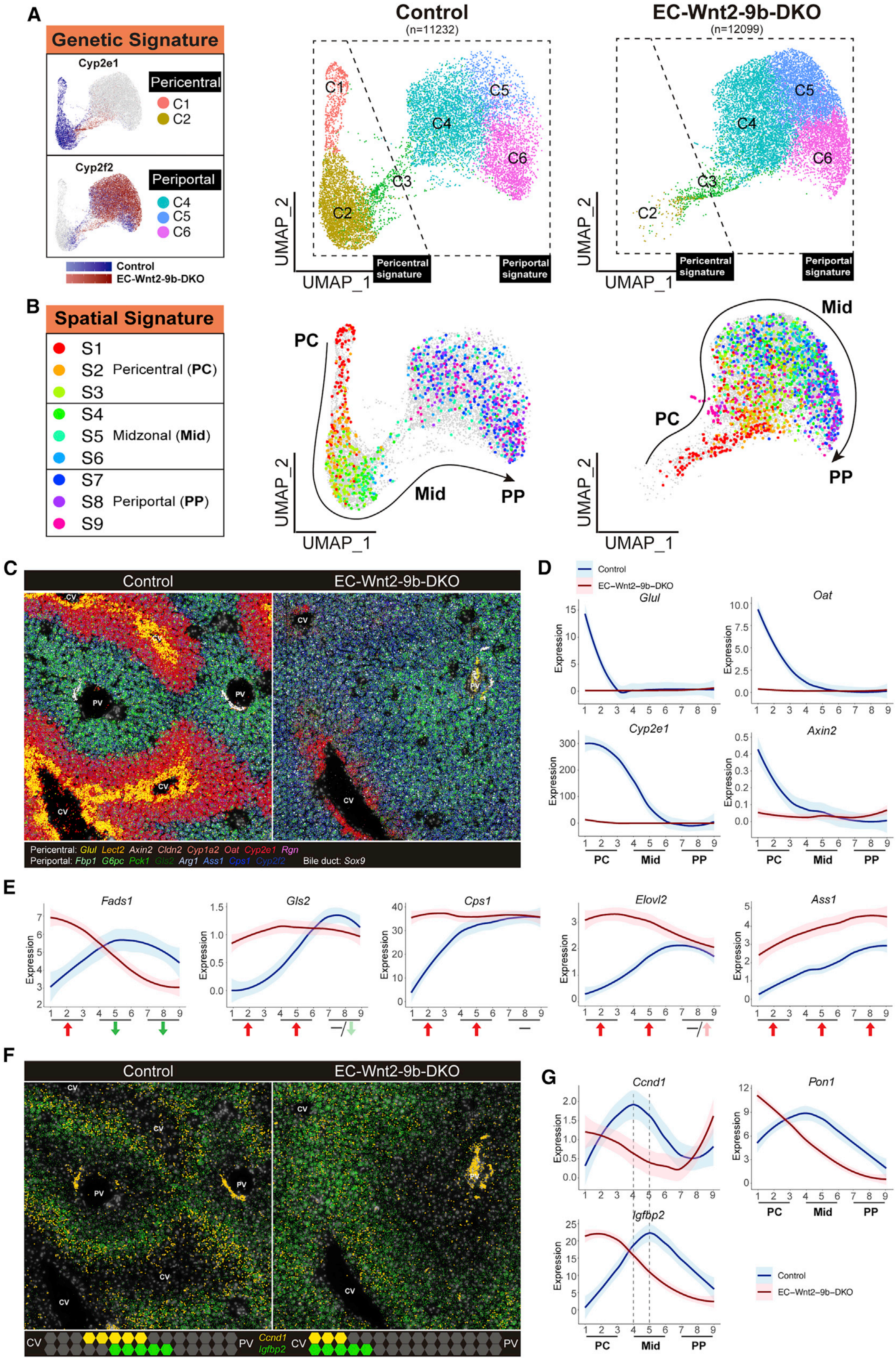

(legend on next page)

counts per area) were quantified within each zone and averaged across the defined pericentral-to-periportal regions thus allowing us to compare gene expression across the lobule using line plots (Figure S10). To combine the genetic signature and spatial signature, we then identified hepatocytes located within the nine segments based on their position (x and y axis) on the slides and applied this information back to the UMAP to track their localization (Figure S10). As expected, in controls, pericentral cells were seen mainly in clusters 1 and 2 and periportal cells in clusters 4, 5, and 6. In EC-Wnt2-9b-DKO mice, most pericentral and all midzonal and periportal cells were in clusters 4, 5, and 6, exhibiting periportal genetic signature (Figure 3B).

We next visualized molecular cartography expression of specific pericentral Wnt targets (*Glul*, *Lect2*, *Axin2*, *Cldn2*, *Cyp1a2*, *Oat*, *Cyp2e1*, and *Rgn*) and of periportal genes (*Fbp1*, *G6pc*, *Pck1*, *Gls2*, *Arg1*, *Ass1*, *Cps1*, and *Cyp2f2*) (Figure 3C). Most pericentral Wnt-β-catenin target genes in hepatocytes were dramatically downregulated or absent in EC-Wnt2-9b-DKO livers, including *Glul*, *Oat*, *Cyp2e1*, *Axin2*, *Cldn2*, *Cyp1a2*, *Cyp7a1*, *Lect2*, *Lgr5*, *Prodh*, *Rgn*, *Rnf43*, and *Tbx3* (Figures 3D and S11A), supporting these β-catenin-TCF4 targets to be under direct control of paracrine Wnt2 and Wnt9b ligands from the neighboring ECs. Intriguingly, not all pericentral genes were affected and paradoxically some genes, such as *Alad*, *C6*, *Cpox*, *Cyp27a1*, *Gstm1*, and *Lrp5*, were upregulated, suggesting their expression to be controlled by other pericentral regulators or may be a consequence of compensation to the Wnt-β-catenin pathway disruption (Figure S11B). Interestingly, we observed *de novo* ectopic expression of periportal genes in zone 3 either with concomitant decrease in their periportal expression (*Fads1*, *C8b*, *Hsd17b13*, *Igf1*, *Pck1*, and *Pigr*) (Figures 3E and S11C), without any change in their baseline periportal expression (*Gls2*, *Cps1*, *Elovl2*, *Arg1*, *Cpt2*, *Cyp8b1*, *Lrp6*, *Ndufb10*, *Uqcrh*, and *Vtn*) (Figures 3E and S11D), or with concurrent increase in their periportal expression (*Ass1*, *Atp5a1*, *Cyp2f2*, *Fbp1*, *Igfals*, and *Sox9*) (Figures 3E and S11E).

To assess if such dynamism in the process of LZ was more global and not limited to one model, we queried expression of selected zonally expressed genes by Molecular Cartography in β-catenin-LKO, LRP5-6-LDKO, and EC-Wls-KO livers. All models showed similar alterations with gain of periportal gene expression in zone 3 hepatocytes along with alterations in their native zone 1 expression (Figure S12).

Zone 2 hepatocyte proliferation has been shown to be at least in part driven by the IGFBP2-mTOR-CCND1 axis.[31] Indeed, *Igfbp2* and *Ccnd1* were both expressed in midzone in the control liver, with highest expression of *Ccnd1* in segment 4 and highest expression of *Igfbp2* in segment 5 (Figure 3G). In EC-Wnt2-9b-DKO mice, *Ccnd1* level was overall decreased (average fold change = 0.84, $p = 1.00654 \times 10^{-55}$), while *Igfbp2* was marginally increased (average fold change = 1.09, p = 1.72255E-10). Importantly, there was decrease of *Ccnd1* in midzone but an increase in pericentral hepatocytes along with an increase in *Igfbp2* (Figures 3F and 3G). The loss of *Ccnd1* from midzone in DKOs suggests its expression is also normally controlled by EC *Wnt2* and *Wnt9b* and from midzonal *Igfbp2*, and the latter may become the dominant regulator in the absence of Wnts. High expression of *Ccnd1* evident in periportal cells is a technical artifact due to inadvertent inclusion of *Ccnd1*-positive biliary cells during cell outlining by the QuPath. Midzonal expression of *Pon1* showed a similar shift (Figure 3G).

Collectively, we observe "periportalization" of the liver lobule in all Wnt-β-catenin pathway disrupted livers. Furthermore, the spatiotemporal changes in the expression of multiple zonated genes in hepatocytes in all zones upon elimination of *Wnt2* and *Wnt9b* that is basally expressed only in zone 3 ECs, or β-catenin signaling that is active only in zone 3 hepatocytes, underscores the overall dynamic nature of LZ. Thus, LZ appears to be a net impact of both active transcription and repression of genes in the same cells.

EC-derived Wnt2 and Wnt9b contribute to normal LR after PH

The Wnt-β-catenin pathway has also been shown to play an important role in regulating hepatocyte proliferation after PH. Loss of β-catenin or LRP5-6 from hepatocytes or Wls from

Figure 3. Single-cell spatial transcriptomics profiling by Molecular Cartography reveals dynamic zonation changes in EC-Wnt2-9b-DKO mice

(A) UMAP plots of single-cell transcriptomic analysis of Molecular Cartography data showing genetic clusters of hepatocytes from control and EC-Wnt2-9b-DKO livers based on expression of 16 zonated genes. Seurat clustering identifies C1-C6 clusters with C1 and C2 clusters representing hepatocytes expressing pericentral (zone 3) genes, C3 representing hepatocytes expressing midzonal genes, and C4-C6 representing hepatocytes expressing periportal genes. An enrichment of C4-C6 hepatocytes is observed in the EC-Wnt2-9b-DKO cluster at the expense of C1 and C2 clusters.

(B) UMAP plots using spatial location of cells in Molecular Cartography analysis based on nine arbitrary but equivalent segments manually drawn between portal vein and central vein shows various clusters recognized in (A) to represent zonal location validating the accuracy of this technique in addressing single-cell spatial transcriptomics.

(C) Molecular Cartography comparing spatial gene expression of *Glul*, *Lect2*, *Axin2*, *Cldn2*, *Cyp1a2*, *Oat*, *Cyp2e1*, and *Rgn* (zone 3), and *Fbp1*, *G6pc*, *Pck1*, *Gls2*, *Arg1*, *Ass1*, *Cps1*, and *Cyp2f2* (zone 1), and *Sox9* (mostly in cholangiocytes) in control and EC-Wnt2-9b-DKO liver showing loss of zone 3 genes and overall "periportalization" of the DKO liver. CV, central vein; PV, portal vein.

(D) Line plots showing impaired pericentral expression of Wnt target genes (shown here are *Glul*, *Oat*, *Cyp2e1*, and *Axin2)* in DKOs compared with control. PC, pericentral; Mid, midzonal; PP, periportal.

(E) Line plots showing dynamic changes in the expression of periportal genes (shown here are *Fads1*, *Gls2*, *Cps1*, *Elovl2*, and *Ass1)* in DKOs compared with control. Red arrow, upregulation; green arrow, downregulation; negative, no change. Color stands for the extent of up- or downregulation.

(F) Molecular Cartography depicting altered spatial expression of *Ccnd1* and *Igfbp2* in EC-Wnt2-9b-DKO versus control liver. While the expression of both genes is midzonal with some overlap, both genes are located pericentrally in DKOs with *Ccnd1* being more restricted to hepatocytes next to the central vein. CV, central vein; PV, portal vein.

(G) Line plots of three representative midzonal genes (*Ccnd1*, *Pon1*, and *Igfbp2)* showing altered location to pericentral hepatocytes in DKOs compared with control. The hue around line plots represents SEM.

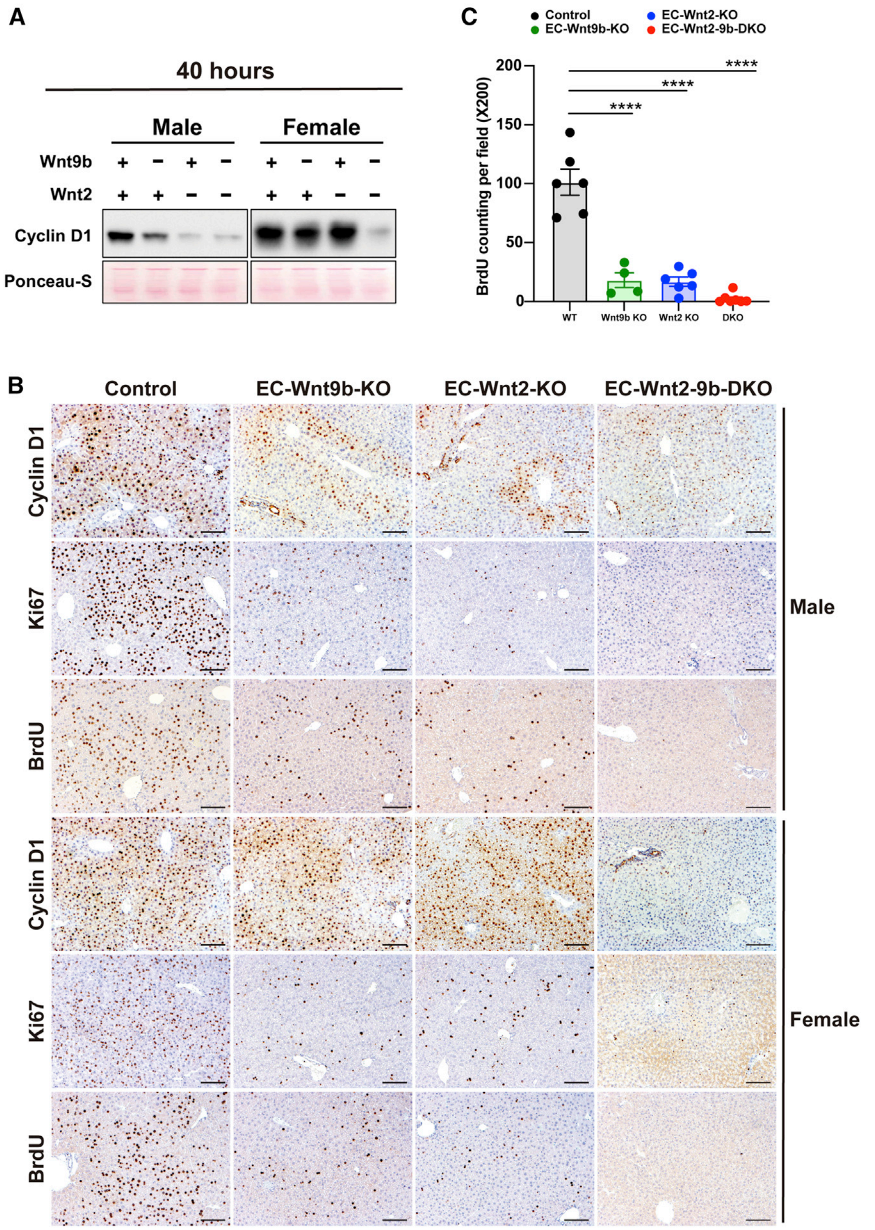

Figure 4. Hepatic EC-derived Wnt2 and Wnt9b contribute to normal liver regeneration after partial hepatectomy

(A) A representative WB showing notably lower cyclin D1 level at 40 h post PH, especially in males in single KO of endothelial cell Wnt2 and Wnt9b, but a more profound decrease is evident in DKOs in both sexes.

(B) IHC for cyclin D1 shows decreased staining at 40 h post PH in male EC-Wnt2-KO, EC-Wnt9b-KO, and EC-Wnt2-9b-DKO mice. Staining for markers of proliferation, such as Ki67 and BrdU, were concurrently decreased in EC-Wnt2-KO and EC-Wnt9b-KO, and almost absent in EC-Wnt2-9b-DKO. In females, cyclin D1 was not changed at 40 h in single KO but was notably decreased in DKOs. Despite no difference in cyclin D1, both Ki67 and BrdU were decreased in single and almost absent in DKOs showing impairment of proliferation. Scale bars, 100 μm.

(C) Quantification of BrdU-positive hepatocytes per field (200×) at 40 h. Both males and females were included. The bars represent means ± SEM, $^{****}p < 0.0001$. n = 6, 4, 6, and 7 mice.

ECs, resulted in decreased cyclin D1 expression in hepatocytes leading to notably lower numbers of hepatocytes in S-phase and decreased proliferation at 40 h, with eventual recovery at 72 h.[13,14,17] To investigate the role of *Wnt2* and *Wnt9b* from ECs in this process, we subjected male and female EC-Wnt2-KO, EC-Wnt9b-KO, and EC-Wnt2-9b-DKO mice to PH. Male EC-Wnt2-KO and EC-Wnt9b-KO mice exhibited a profound decrease in cyclin D1 (Figure 4A). This decrease was less conspicuous in females, likely due to basally higher cyclin D1 levels (Figures 2B and 4A). Importantly, like β-catenin-LKO, LRP5-6-LDKO, and EC-Wls-KO, cyclin D1 was barely detectable in the EC-Wnt2-9b-DKO mice in both genders, suggesting that the two Wnts are collectively required for normal cyclin D1 upregulation during LR (Figure 4A).

We next assessed the localization of cyclin D1-positive hepatocytes by IHC. In controls, periportal and midzonal hepatocytes were cyclin D1 positive, while one to two layers of hepatocytes around the central vein remained negative (Figure 4B). In single KOs, cyclin D1-positive hepatocytes were concentric around the central vein except for one to two layers of hepatocytes immediately around the vessel (Figure 4B). In EC-Wnt2-9b-DKO mice, cyclin D1 was weak but more diffusely expressed. These observations suggest that the concentric pattern of cyclin D1 in the EC-Wnt2-KO mice may be due to reciprocal increase in Wnt9b in pericentral neighborhood and vice versa in the EC-Wnt9b-KO mice (Figure 4B). But their combined loss prevented such localization of cyclin D1. Unlike males, female single KO lacked any peculiarities in cyclin D1 levels or localization, and only DKO mice showed a dramatic decrease in cyclin D1-expressing hepatocytes (Figure 4B). To study the consequence of cyclin D1 decrease we compared LR in controls, single, and double KO livers at 40 h by Ki67, a marker of S-phase, and BrdU, an indicator of cell proliferation, injected to mice 5 h before euthanasia. Like cyclin D1, proliferating hepatocytes were mainly localized around the periportal and midzonal region in the controls. Single KO mice had sparsely positive cells, and DKO mice were completely negative for Ki67 and BrdU labeling at 40 h (Figures 4B and 4C). These observations were consistent in both genders. Therefore, there is a notable deficit in LR in EC-Wnt2-KO and EC-Wnt9b-KO mice, which is even

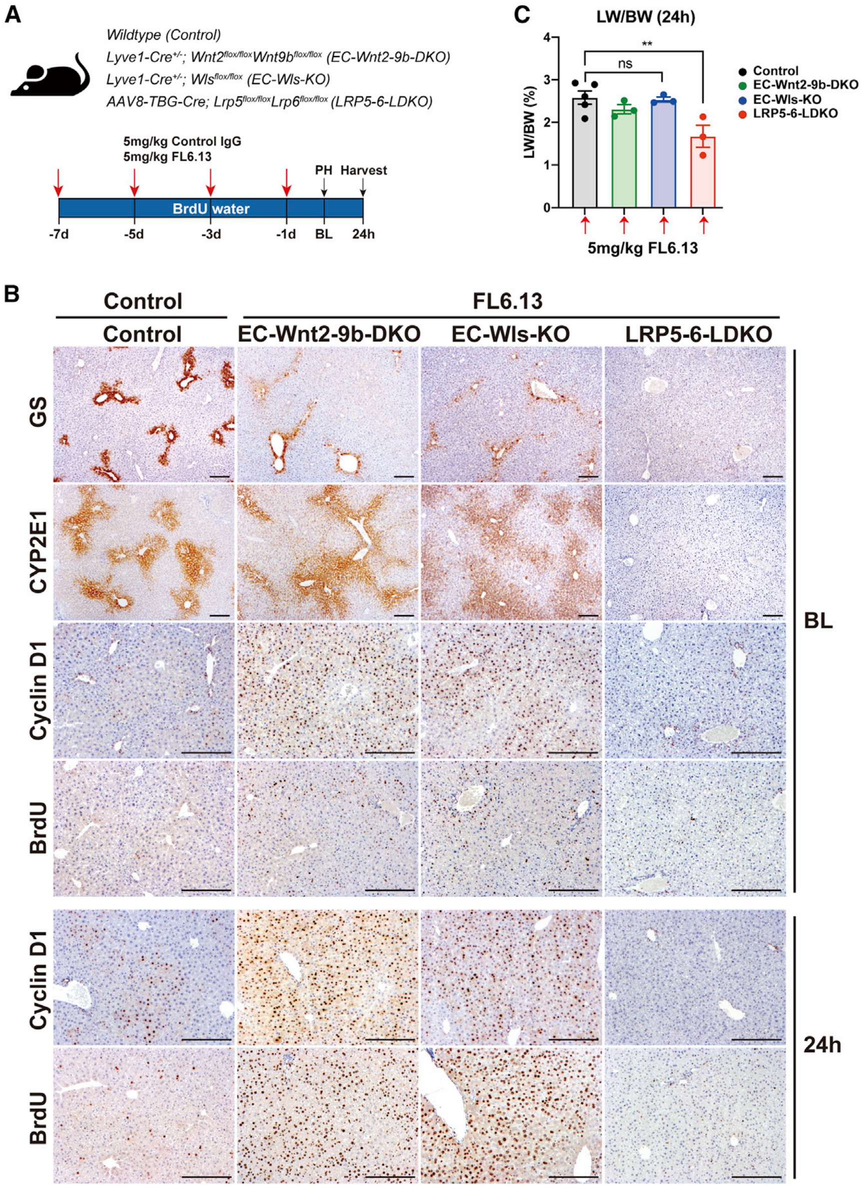

Figure 5. FL6.13 rescues liver zonation and liver regeneration in mice lacking Wnt secretion, specifically Wnt2 and Wnt9b from the endothelial cells, but not in mice lacking Wnt co-receptors LRP5-6 from hepatocytes

(A) Study design showing dosing schedule of pan-Frizzled agonist FL6.13 or isotype control IgG administration to various mouse groups.

(B) Representative IHC for β-catenin pericentral targets GS and CYP2E1, midzonal target cyclin D1, and indicator of cell proliferation BrdU, in baseline livers after four treatments of control mice with control IgG and four treatments of EC-Wnt2-9b-DKO, EC-Wls-KO, and LRP5-6-LDKO mice with FL6.13. Re-appearance of these markers to almost control levels is seen in EC-Wnt2-9b-DKO and EC-Wls-KO mice but not in LRP5-6-LDKO mice. Scale bars, 200 μm.

(C) Bar graph for LW/BW (± SEM) at 24 h post PH shows comparable liver restoration in FL6.13-treated controls, EC-Wnt2-9b-DKO, and EC-Wls-KO mice, but not LRP5-6-LDKO mice. n.s., not significant, $^{**}p < 0.01$. n = 5, 3, 3, 3 mice).

worse in EC-Wnt2-9b-DKO mice phenocopying β-catenin-LKO, LRP5-6-LDKO, and EC-Wls-KO mice.[13,14,17]

FL6.13 rescues metabolic LZ and delayed LR in Wnt-deficient mice

Since FL6.13, the tailored tetravalent antibody has recently been shown to engage the FZD-LRP6 receptor in the absence of natural Wnt ligands, we next investigated if it could activate β-catenin signaling and rescue both the pericentral gene expression or metabolic LZ as well as LR after PH in the EC-Wnt2-9b-DKO and in EC-Wls-KO mice.[17] Four doses of control IgG or FL6.13 were injected into 8-week-old male KO or control mice every other day for a week as described in STAR Methods (Figure 5A). PH was performed on day 8 and the resected livers were processed for analysis. Regenerating livers were also harvested at 24 h post PH. Mice were given 1 mg/mL BrdU in drinking water throughout the study to label all proliferating cells during the process (Figure 5A). Pretreatment with FL6.13 restored pericentral expression of GS and pericentral and midzonal expression of CYP2E1 in both models (Figure 5B). FL6.13 was also efficient in inducing pan-zonal expression of cyclin D1 except in the most immediate one to two layers of pericentral hepatocytes (Figure 5B). Concomitantly, hepatocyte proliferation as seen by enhanced BrdU incorporation was evident in both groups of KOs at baseline (Figure 5B). At 24 h after PH, a profound increase in hepatocyte proliferation was observed in EC-Wnt2-9b-DKO and EC-Wls-KO mice by BrdU incorporation and LW/BW recovery, which was even greater than the control animals dosed with control IgG, suggesting rescue and even shift to the left in LR kinetics with FL6.13 (Figures 5B and 5C). This enhanced proliferation in response to FL6.13 in both KOs was associated with continued increase in cyclin D1 in most hepatocytes pan-zonally.

To address the specificity of the response by FL6.13, we next treated LRP5-6-LDKO mice lacking Wnt-co-receptors in hepatocytes.[14] These mice have been shown previously to also phenocopy β-catenin-LKO in both lacking pericentral Wnt-β-catenin targets and delayed LR. When these mice were treated with FL6.13 (Figure 5A), there was no change in pericentral gene expression of GS and CYP2E1, which continued to be absent in these mice (Figure 5B). Similarly, FL6.13 was unable to restore either cyclin D1 expression or BrdU incorporation either at baseline or 24 h post PH (Figure 5B). This was also reflected by a

CellPress
OPEN ACCESS

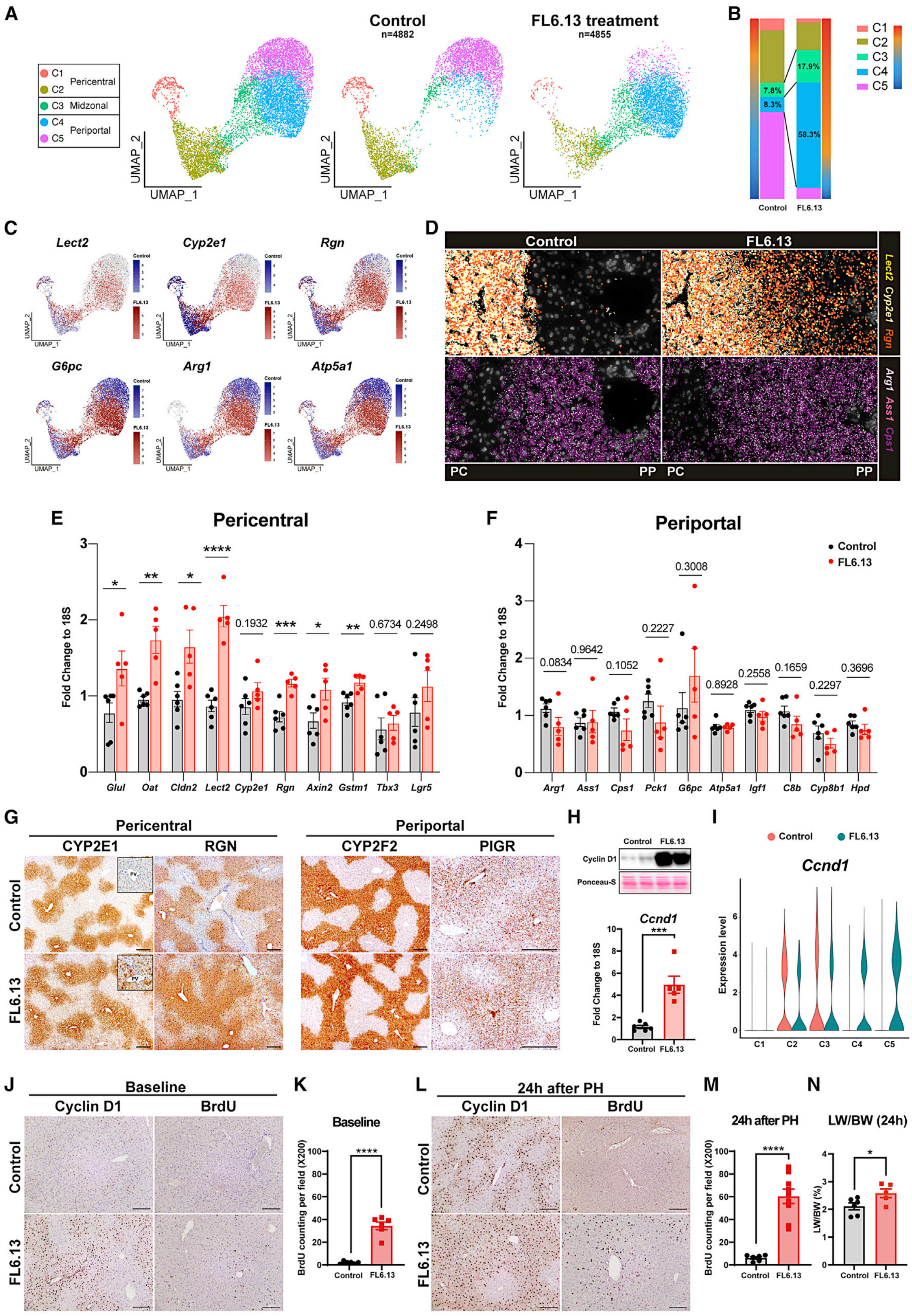

(legend on next page)

deficient LW/BW at 24 h post PH (Figure 5C). This study shows the requirement of intact Wnt co-receptors for FL6.13 to stimulate the Wnt-β-catenin signaling *in vivo*.

FL6.13 induces pericentral gene expression at baseline

To characterize Wnt-β-catenin activation by FL6.13 in greater depth, we tested the effect of four doses of FL6.13, given every 48 h, on the wild-type mice, as described in STAR Methods. Twenty-four hours after the last injection, the mice were euthanized, and livers were processed for Molecular Cartography using the same set of probes (Table S3). Two pipelines were applied for analyses (Figure S13). Zonal distribution of hepatocytes was identified based on differentially expressed genes (Figure S14) and confirmed by tracing back the localization and zonation of hepatocytes on the slides (Figure S13). The genetic signature and spatial signature overlapped quite well on the UMAP for the current analysis, unlike the EC-Wnt2-9b-DKO mice that lacked Wnt-β-catenin target genes in zone 3 hepatocytes precluding overlap (Figure S13). Five different clusters were identified, which enabled reconstruction of the metabolic zones (pericentral: clusters 1 and 2; midzonal: cluster 3; periportal: clusters 4 and 5) (Figure 6A). A notable increase of the proportion of C3 and C4 was noted after FL6.13 treatment (Figure 6B), which was marked by the ectopic expression of pericentral Wnt target genes, such as *Lect2*, *Cyp2e1*, *Rgn*, *Cyp1a2*, *Gstm1*, and *Cldn2* (Figures 6C and S15A). Interestingly, some pericentral genes were not induced by FL6.13 using spatial single-cell analysis, including transcription factor *Tbx3*,[32] heme synthesis enzymes *Alad* and *Cpox*,[33] bile acid synthesis enzyme *Cyp27a1*,[34] and complement pathway gene *C6*[34] (Figure S15B). This indicated that FL6.13 selectively activates some but not all pericentral genes. Unlike de-repression of periportal genes in the EC-Wnt2-9b-DKO mice, there was no concomitant decrease in the expression of periportal genes, including complement pathway genes (*C8b*, *Vtn*), Fc receptor (*Pigr*), lipid metabolism gene (*Hsd17b13*), oxidative phosphorylation genes (*Uqcrh*, *Atp5a1*), gluconeogenesis genes (*G6pc*, *Fbp1*, *Pck1*), glutamine catabolism gene (*GLs2*), urea cycle genes (*Arg*, *Cps1*), and hormone (*Igf1*) (Figures 6C and S16A). Representative images from Molecular Cartography showed that FL6.13 induced an expansion of pericentral markers (*Lect2*, *Cyp2e1*, *Rgn*) to both midzonal and periportal regions, while the expression of periportal markers (*Arg1*, *Ass1*, *Cps1*) remained unaltered (Figure 6D).

We also validated some of these findings by qPCR. Indeed, most pericentral Wnt-β-catenin target genes, such as *Glul* and *Axin2*, were significantly induced by FL6.13 treatment, whereas periportal genes were not affected (Figures 6E and 6F). Not all pericentral genes were increased by FL6.13. Levels of *Tbx3* and *Lgr5* were unchanged after FL6.13 treatment (Figure 6E).

To visualize zonation changes at the protein level, we stained pericentral markers (CYP2E1 and RGN) and periportal markers (CYP2F2 and PIGR) in control and FL6.13-treated livers. Expansion and ectopic expression of CYP2E1 and RGN was observed at the midzone and in the periportal hepatocytes after FL6.13 treatment, while areas of CYP2F2 and PIGR immunostaining were similar as controls and in periportal hepatocytes (Figure 6G).

Altogether, these data indicate a selective and potent effect of FL6.13 in inducing expansion of most pericentral Wnt-β-catenin target genes but not at the expense of periportal gene expression.

Figure 6. Single-cell spatial transcriptomics profiling of FL6.13-treated control mice reveals dynamic changes in zonation and promotes regeneration after partial hepatectomy

(A) UMAP plots of single-cell transcriptomic analysis of hepatocytes by Molecular Cartography showing gain of specific clusters in FL6.13-treated livers.

(B) Stacked bar chart depicting distribution of various hepatocyte clusters showing specific gain of C3 and C4 after FL6.13 treatment (right bar) compared with controls (left bar).

(C) Feature plots from Molecular Cartography analysis of specific representative genes showing ectopic expression of pericentral genes (*Lect2*, *Cyp2e1*, *Rgn*) in midzonal and periportal cluster after FL6.13 treatment (red) compared with controls (blue), while periportal genes (*G6pc*, *Arg1*, *Atp5a1*) continued to be expressed in these clusters and hence remained unchanged.

(D) Molecular Cartography visualization of tissue section of the same genes as indicated in (C) showing ectopic expression of pericentral genes in midzonal and periportal regions, while periportal genes were unchanged after FL6.13 treatment. PC, pericentral; PP, periportal.

(E) qPCR using bulk mRNA from livers of controls or FL6.13-treated mice shows significant increase in the expression of several pericentral Wnt target genes by FL6.13. *$p < 0.05$, **$p < 0.01$, ***$p < 0.001$, ****$p < 0.0001$. n = 6, 5 mice.

(F) qPCR using bulk mRNA from livers of controls or FL6.13-treated mice shows no statistically significant effect on expression of several known periportal genes by FL6.13. Corresponding p values are indicated. n = 6, 5 mice.

(G) Representative IHC verify ectopic localization of CYP2E1 and RGN proteins in midzonal and periportal regions after FL6.13 treatment, while CYP2F2 and PIGR protein locations remained unchanged. PV, portal vein. Scale bars, 200 μm.

(H) Representative qPCR using whole-liver RNA and WB using whole-liver lysate shows dramatic increase in *Ccnd1* expression and cyclin D1 protein after FL6.13 treatment compared with controls. qPCR: n = 6, 5 mice.

(I) Violin plot of Seurat clustering of the Molecular Cartography from (A) shows appearance of periportal *Ccnd1* expression (C4 and C5 clusters; green) while its expression in C2 and C3 clusters, where it is normally expressed in controls (red), remains unchanged after FL6.13 treatment.

(J) Representative IHC for cyclin D1 and BrdU showing FL6.13 induces periportal hepatocyte G1 to S-phase transition and hepatocyte proliferation, respectively, compared with controls. Scale bars, 100 μm.

(K) Quantification of BrdU-positive hepatocytes (± SEM) in (J) shows that FL6.13 induces significant increase in hepatocyte proliferation at baseline compared with control IgG. ****$p < 0.0001$. n = 6, 6 mice.

(L) Representative IHC showing that FL6.13 accelerates liver regeneration at 24 h post PH by promoting the number of cyclin D1-positive periportal and midzonal hepatocytes and concurrently increasing BrdU-positive proliferating periportal and midzonal hepatocytes compared with control IgG treatment. Scale bars, 100 μm.

(M) Quantification of BrdU-positive hepatocytes (± SEM) in (L) shows that FL6.13 induces significant increase in hepatocyte proliferation at 24 h post hepatectomy compared with control IgG treatment. ****$p < 0.0001$. n = 6, 9 mice.

(N) LW/BW (± SEM) at 24 h post PH depicts an advantage of liver mass restoration in the FL6.13 versus control IgG-treated group. *$p < 0.05$. n = 6, 5 mice.

FL6.13 induces *Ccnd1* gene expression, hepatocyte proliferation, and promotes LR after PH

Since FL6.13 effectively promoted LR after PH in the EC-Wnt2-9b-DKO and EC-Wls-KO mice through stimulation of *Ccnd1* expression, we next queried its impact on normal baseline liver after four doses of FL6.13 given every 48 h. A notable increase in *Ccnd1* mRNA and cyclin D1 protein was observed after FL6.13 treatment (Figure 6H). Interestingly, induction of cyclin D1 was not evenly distributed across the liver lobule, but only around the periportal region (Figures 6I and 6J), like observations with another Wnt agonist and RSPO1.[6,35] BrdU incorporation was significantly induced after FL6.13 treatment, indicating that periportal hepatocytes entered S-phase upon Wnt activation and cyclin D1 expression (Figures 6J and 6K). Collectively, FL6.13 could activate the hepatic Wnt-β-catenin pathway, induce expansion of pericentral metabolic LZ through midzone and even periportally, without impairing expression and localization of periportal genes, and while provoking proliferation of periportal and midzonal hepatocytes. These data also suggest that hepatocytes within different zones in the liver have distinct responses to exogenous Wnt stimulation.

Considering the strong effect of FL6.13 in inducing hepatocyte proliferation, as a proof-of-concept for therapeutic intervention, we next investigated the effect of FL6.13 on LR using the PH model. Four doses of control IgG or FL6.13 were i.p. injected into 8-week-old male C57BL/6 mice. PH was performed on day 8 and regenerating livers were harvested 24 h later. Mice were given 1 mg/mL BrdU in drinking water to label proliferating cells throughout the process (Figure S16B). In control IgG-treated mice, cyclin D1 was expressed mainly at the midzone at 24 h post PH, and very few hepatocytes were BrdU positive (Figure 6L). Pretreatment with FL6.13 induced dramatic expansion of cyclin D1 toward pericentral region and 6-fold increase in periportal BrdU incorporation (Figures 6L and 6M). Proliferative advantage led to accelerated LR and contributed to significantly greater recovery of LW/BW at 24 h (Figure 6N). These results demonstrate a potent effect of FL6.13 pretreatment in promoting LR leading to faster recovery of hepatic mass.

Late treatment with FL6.13 promotes liver repair after APAP injury by promoting Wnt-β-catenin activation and hepatocyte proliferation

To test the clinical applicability of FL6.13 to promote repair in a model of acute liver insult, we evaluated its efficacy in APAP overdose-induced hyperacute liver injury. APAP is a widely used analgesic and antipyretic, but it accounts for 46% of acute liver failure (ALF) in the United States.[36] Patients with APAP overdose either progress to liver failure requiring transplant or may recover spontaneously. N-Acetyl cysteine (NAC) is the only currently approved antidote but must be given early after APAP ingestion for its full efficacy, and the probability of developing ALF continues to increase over time with delay in NAC administration.[37]

Eight-week-old male C57BL/6 fasted for 12 h were injected with 600 mg/kg APAP followed by a single dose of control IgG or FL6.13 at 12 h and mice were sacrificed at 48 h post APAP injection (Figure S17A). Surprisingly, mice treated with FL6.13 had higher mortality (Figure S17B), accompanied with 2-fold higher ALT level (Figure S17C). Negative effect of FL6.13 was seen as patches of necrotic areas by hematoxylin and eosin (Figure S17D). Severe gross and sinusoidal congestion was seen in the dead mice after FL6.13 treatment (Figures S17B and S17D). We next evaluated hepatocyte proliferation, which counteracts the injury to drive liver repair. APAP (600 mg/kg) has been reported to induce severe liver injury with mild hepatocyte proliferation during the recovery process.[38] Indeed, in the control group cyclin D1 was weakly expressed only by the first layer of hepatocytes surrounding pericentral necrotic regions at 48 h post APAP. Hepatocytes were negative for Ki67 demonstrating lack of G1 to S-phase transition at this time (Figure S17D). Interestingly, FL6.13 treatment did not induce cyclin D1 and Ki67 at 48 h after APAP overdose (Figure S17D). Comparable immune cell infiltration, important for phagocytosis of necrotic cells and subsequent liver repair, was evident in both control IgG and FL6.13-treated mice, shown by IHC of CD45 (Figure S17D).[39] CYP2E1 plays an essential role in APAP metabolism to generate N-acetyl-p-benzoquinone imine (NAPQI), a highly reactive metabolite that leads to hepatotoxicity.[40] Since FL6.13 was shown to induce pericentral and periportal CYP2E1 expression in wild-type mice (Figure 6G), it is likely that 12 h post 600 mg/kg APAP injection, remnant APAP in mice was inadvertently metabolized to NAPQI by FL6.13-induced CYP2E1 expression, leading to a more severe liver injury. Our data suggest that early intervention with a Wnt agonist may have unintended consequences and may worsen APAP-induced liver injury due to induction of CYP2E1.

Next, we investigated if late treatment of FL6.13 could have any therapeutic benefit through promoting regeneration, especially when APAP has already been metabolized. Such opportunity represents an unmet clinical need since there are no therapies currently available if NAC fails to prevent ALF when patients arrive for clinical intervention late after APAP overdose. Single dose of control IgG or FL6.13 was administrated at 32 h post 600 mg/kg APAP injection, and mice were sacrificed at 60 h (Figure 7A). Grossly, livers displayed normal color and appeared healthier in the FL6.13 group (Figure 7B). FL6.13-treated mice showed significantly reduced serum ALT, while AST trended favorably as well (Figure 7C). The control group showed pericentral necrotic areas at 60 h post APAP injection with increased expression of cyclin D1 in peri-necrotic hepatocytes (Figure 7D). Ki67-positive hepatocytes were seen surrounding necrotic regions and in hepatocytes in the periportal region (Figure 7D). FL6.13-treated mice exhibited a significantly reduced necrotic area, which was associated with a profound increase in cyclin D1-expressing hepatocytes pan-zonally, indicating that these cells are all able to enter the cell cycle across the liver lobule (Figures 7D and 7E). Many periportal hepatocytes, as well as pericentral hepatocytes, were Ki67 positive in the FL6.13-treated group, which was significantly more than the isotype control-treated group (Figures 7D and 7F). FL6.13 treatment showed a more localized immune cell response around necrotic regions, as seen by staining for CD45 (Figure 7D). Altogether, delayed Wnt agonism promotes liver repair after acute APAP injury by inducing hepatocyte proliferation that occurs locally and periportally and may be "pushing" hepatocytes toward the central vein to fill the space created by the cleared necrotic debris by

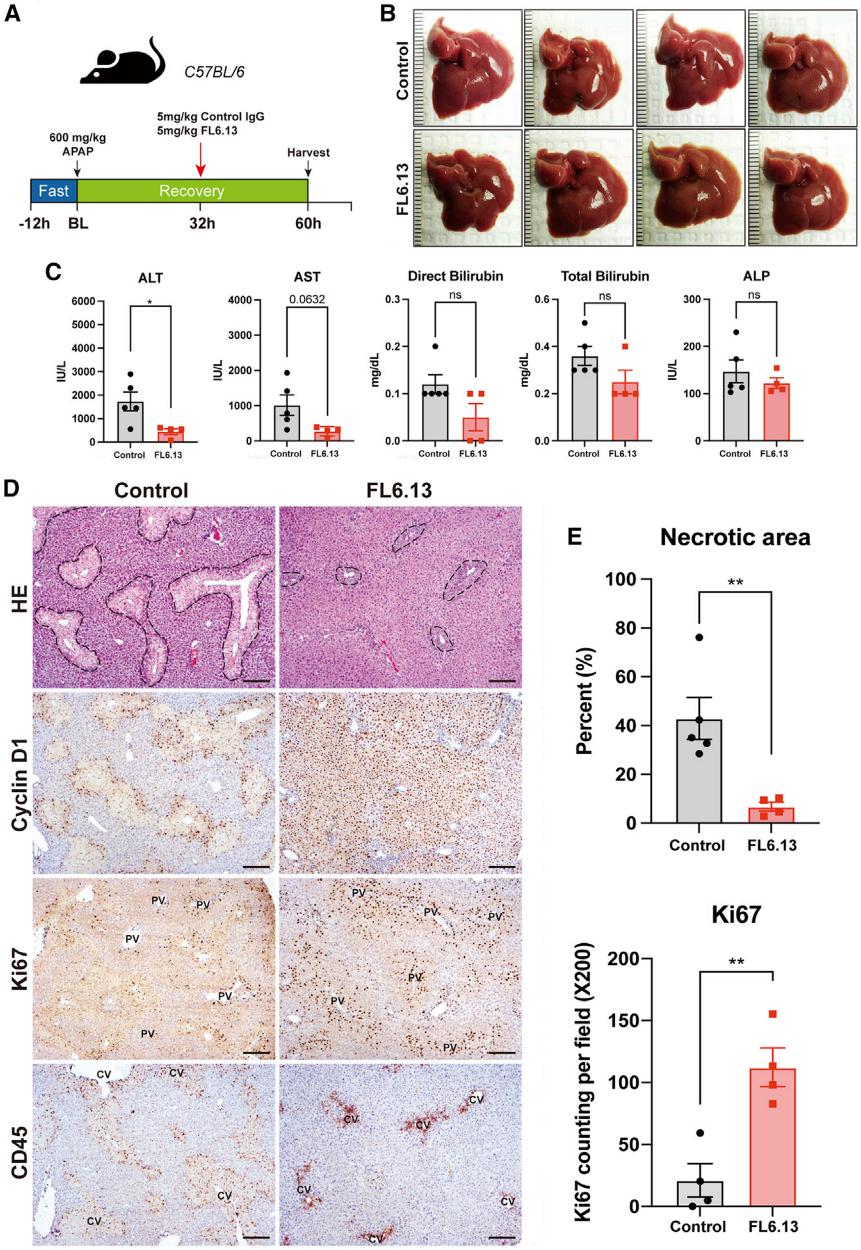

Figure 7. Late treatment with FL6.13 promotes liver repair after APAP injury through induction of hepatocyte proliferation

(A) Study design showing administration of a single dose of 5 mg/kg pan-FZD agonist FL6.13 or isotype control IgG at 32 h post 600 mg/kg i.p. APAP injection. Mice were sacrificed at 60 h for analysis.

(B) Gross images of livers from FL6.13- and control IgG-treated groups after APAP showing decreased necrosis and congestion at 60 h in the FL6.13 treatment group.

(C) Serum ALT, AST, direct and total bilirubin, and ALP levels (± SEM) at 60 h after APAP showing significantly reduced serum ALT and AST (trending favorably) in FL6.13 compared with control IgG. n.s., not significant, $^{*}p < 0.05$. n = 5, 4 mice.

(D) Representative IHC showing decreased necrotic areas by hematoxylin and eosin (H&E) staining, pan-lobular cyclin D1 staining, increased periportal cell proliferation by Ki67 staining, and a more localized immune cell response by CD45 staining after FL6.13 treatment compared with control IgG. CV, central vein; PV, portal vein. Scale bars, 200 μm.

(E) Quantification of H&E and Ki67 showing significant decrease in necrotic areas and increase in hepatocyte proliferation in the FL6.13 group. $^{**}p < 0.01$. Necrotic area: n = 5, 4 mice. Ki67: n = 4, 4 mice.

the immune cells. Thus, these data extend the application of FL6.13 as delayed regenerative treatment choice in APAP-associated ALF and other relevant indications.

DISCUSSION

Liver has a unique capacity to regenerate after PH.[9,10] This allows surgical resection of part of liver for oncological indications and permits living donor and split-liver transplantation. LZ uniquely allows the hepatocytes located in various zones to perform specialized functions and also limits some injuries and disease processes to specific zones.[7,8] Many studies show β-catenin as key regulator of LZ and LR.[5] In view of this, it is important to answer, "Who regulates the regulator?" While *Wnt2* and *Wnt9b* have been shown to be expressed in the central venous and pericentral sinusoidal ECs,[19] and their upregulation observed in various liver injuries,[17,20,21] their unequivocal roles in LZ and LR have not been established. Through genetic elimination of Wnt2 and Wnt9b from ECs, we provide conclusive evidence that these molecules play additive roles in instructing LZ and in driving hepatocyte proliferation after PH, despite a broader *Wnt2* expression. We observed gender differences such that females were able to compensate singular *Wnt2* or *Wnt9b* loss better than males, which requires further investigation. Also, Wnt2 and Wnt9b are conserved in their high expression in hepatic ECs in multiple species, suggesting that these two Wnts may be most physiological for use in hepatic regenerative medicine for regulating LZ and LR, just like Wnt7a, Wnt7b, and Wnt10a, have been shown to be relevant during cholestasis.[41] Finally, based on analysis of human scRNA sequencing data from hepatic ECs, and location of β-catenin targets *GLUL*, *AXIN2*, and *CYPs* in zone 3 hepatocytes, it is likely that the same *WNTs* are also playing a role in human LZ.[7,42,43]

Although previous studies have hinted at periportal genes being ectopically expressed in zone 3 hepatocytes upon β-catenin inhibition, the conclusions have been limited by lack of sensitive

technical tools.[4] Molecular Cartography enabled us to evaluate single-cell spatial expression of 100 genes on the same slide to analyze changes after genetic or pharmacological intervention. Such profiling of EC-Wnt2-9b-DKO livers revealed induction of many periportal genes *de novo* in pericentral hepatocytes. Intriguingly, for some genes, this change occurred simultaneously with increase, decrease, or no change in their normal baseline zonal expression. Loss of Wnt2 and Wnt9b or Wntless from hepatic ECs, or loss of β-catenin or LRP5-6 from hepatocytes, all led to similar absence of pericentral genes while simultaneously de-repressing and altering periportal gene expression. This suggests LZ to be a highly dynamic process constituted by simultaneous transcriptional activation and repression of genes in each zone. Periportalization of EC-Wnt2-9b-DKO mice led to resistance to APAP injury, as shown previously for other genetic mouse models of perturbed Wnt-β-catenin signaling.[14,17,44] Interestingly, administration of Wnt agonist FL6.13 to a control animal induced zone 3 gene expression in periportal hepatocytes, but it did not result in loss of zone 1 genes. One possible explanation for the differences could be permanent genetic elimination versus transient effect brought about by administration of an exogenous molecule. Another possibility is the differential zonal dynamism of gene expression in hepatocytes residing in zone 3 versus zone 1. It is conceivable that, by default, all hepatocytes expressed periportal genes; however, hepatocytes in zone 3 acquire their zone 3 identity by exposure to neighborhood signals, such as from ECs, which led to repression of periportal genes while activating pericentral genes. Indeed, hepatocytes derived from differentiation of liver stem cells *ex vivo*, show spontaneous zone 1 expression, while zone 3 gene expression needed to be forced by β-catenin activation.[45] While further studies are needed to address how zone 3 hepatocytes shift to periportal identity in the EC-Wnt2-9b-DKO mice, previous studies have also shown that HNF4α inhibits pericentral β-catenin targets.[46] In the absence of β-catenin, TCF4 associated with HNF4α could possibly bind to HNF4α-responsive elements, inducing the expression of periportal genes, and could be one explanation for our observations.[47,48] What regulates Wnt2 and Wnt9b expression and secretion at baseline in the ECs in zone 3, and what stimulates their immediate-early upregulation and release after PH, remains unknown.

Being a critical pathway in modulating both hepatocyte metabolism and proliferation, activating the Wnt-β-catenin to promote hepatic function and restore mass is an attractive therapeutic strategy. We utilized FL6.13, a tetravalent antibody described recently, which induces FZD-LRP6 engagement to induce β-catenin activation.[24] FL6.13 rescued pericentral LZ in EC-Wnt2-9b-DKO and EC-Wls-KO mice but not in LRP5-6-LDKO mice. FL6.13 expanded pericentral Wnt target genes in zone 1 and zone 2 but not at the expense of periportal genes. It simultaneously induced *Ccnd1* expression, allowing hepatocytes to enter the cell cycle and in this way stimulated liver repair without impacting metabolic function. Such duality of Wnt agonism to induce cell proliferation while maintaining metabolic function could benefit treatment of acute and chronic liver insufficiency. While other agonists of Wnt signaling have been generated, our molecule directly engages Wnt receptor and co-receptor and hence is likely more potent and specific.[6,49] A direct comparison with such agents is not currently feasible but may be necessary in the future. FL6.13 has been shown to activate Wnt signaling at other sites, including eye and intestines, based on the method of delivery.[24,50] It did not show any adverse effects for the duration in our study as well as in the aforementioned studies.

One specific and highly relevant unmet clinical need is delayed treatment of patients after APAP overdose. NAC is the only approved therapy for APAP overdose. If given within 4–10 h of APAP overdose, hepatoxicity could still develop in 6.1% cases. The chances of developing liver failure increases to 26.4% and 41% if NAC is administered 10–24 h or beyond 24 h of APAP overdose, respectively.[51] There is no effective therapy beyond 24 h and patients will either spontaneously recover or require transplant. Our data provide a new treatment option of administering regenerative therapy to cases who are either delayed seeking medical attention or progress to failure despite NAC administration. The advantage of use of FL6.13 is that it enhances pericentral LZ while simultaneously promoting zone 3 and 1 hepatocyte proliferation. The periportal hepatocyte proliferation may be creating a push for hepatocytes to move pericentrally to restore microarchitecture in this area as necrosis is cleared by the immune cells. However, premature Wnt stimulation could inadvertently induce more injury through induction of β-catenin targets, such as CYP2E1 and CYP1A2, which generate toxic APAP metabolites, and hence timing of intervention with FL6.13 will be critical, and likely be monitored by serum APAP levels. However, regenerative therapy fills an important niche in the treatment of ALF due to APAP and perhaps other causes.

Limitations of the study

Our study shows role of Wnt-β-catenin agonism in promoting zone 3 gene expression and in inducing LR in two clinically relevant models. There are, however, limitations of our study. First, animal models of liver injury, such as >85% or extended hepatectomy or lethal doses of APAP, lead to quick demise of mice leaving an abbreviated and sometimes variable therapeutic intervention opportunity for any agent to act and reproducibly demonstrate survival efficacy. This limited our ability to show the survival advantage of Wnt agonism. Secondly, other inducers of the Wnt-β-catenin signaling pathway have been reported. We were unable to directly compare the efficacy of FL6.13 with such agonists, which could have provided important information regarding superiority or advantage of one subclass over another. Future studies should directly address these two important shortcomings of our study.

STAR★METHODS

Detailed methods are provided in the online version of this paper and include the following:

- KEY RESOURCES TABLE
- RESOURCE AVAILABILITY
  - Lead contact
  - Materials availability
  - Data and code availability

- EXPERIMENTAL MODEL AND SUBJECT DETAILS
  - Animals
- METHOD DETAILS
  - Two-third or partial hepatectomy
  - FL6.13 treatment
  - Acetaminophen study
  - scRNA sequencing data analysis
  - Molecular cartography^{TM}
  - RNA isolation and qPCR
  - Protein isolation and western blot
  - Immunohistochemistry
  - Immunofluorescence
- QUANTIFICATION AND STATISTICAL ANALYSIS

SUPPLEMENTAL INFORMATION

Supplemental information can be found online at https://doi.org/10.1016/j.xcrm.2022.100754.

ACKNOWLEDGMENTS

This work was supported by NIH grants 1R01CA251155, 1R01CA204586, 1R01DK62277, and 1R01DK116993; Endowed Chair for Experimental Pathology to S.P.M.; and by NIH grant 1P30DK120531 to Pittsburgh Liver Research Center (PLRC) for services provided by the Genomics and Systems Biology Core. This research was also supported in part by the University of Pittsburgh Center for Research Computing through the resources provided. This work was also supported by the University of Toronto Medicine by Design program (grant no. 72058117), which receives funding from the Canada First Research Excellence Fund (to S.S. and S.A.).

AUTHOR CONTRIBUTIONS

Conceptualization, S.H. and S.P.M.; methodology, S.H., S.L., Y.B., M.P., S.S., C.C., J.M., A.B., and S.P.M.; investigation, S.H., S.L., Y.B., M.P., S.S., C.C., and J.M.; writing, S.H. and S.P.M.; funding acquisition, S.P.M.; resources, L.L.B., J.J.A., S.S.S., and S.A.

DECLARATION OF INTERESTS

S.P.M. is a consultant for Surrozen. L.L.B., J.J.A., S.S.S., and S.A. are shareholders of AntlerA Therapeutics.

Received: March 26, 2022
Revised: July 4, 2022
Accepted: September 8, 2022
Published: October 18, 2022

REFERENCES

1. Clevers, H., Loh, K.M., and Nusse, R. (2014). Stem cell signaling. An integral program for tissue renewal and regeneration: Wnt signaling and stem cell control. Science *346*, 1248012. https://doi.org/10.1126/science.1248012.
2. Russell, J.O., and Monga, S.P. (2018). Wnt/beta-Catenin signaling in liver development, homeostasis, and pathobiology. Annu. Rev. Pathol. *13*, 351–378. https://doi.org/10.1146/annurev-pathol-020117-044010.
3. Burke, Z.D., Reed, K.R., Phesse, T.J., Sansom, O.J., Clarke, A.R., and Tosh, D. (2009). Liver zonation occurs through a beta-catenin-dependent, c-Myc-independent mechanism. Gastroenterology *136*, 2316–2324.e1-3. https://doi.org/10.1053/j.gastro.2009.02.063.
4. Benhamouche, S., Decaens, T., Godard, C., Chambrey, R., Rickman, D.S., Moinard, C., Vasseur-Cognet, M., Kuo, C.J., Kahn, A., Perret, C., and Colnot, S. (2006). Apc tumor suppressor gene is the "zonation-keeper" of mouse liver. Dev. Cell *10*, 759–770. https://doi.org/10.1016/j.devcel.2006.03.015.
5. Hu, S., and Monga, S.P. (2021). Wnt/-Catenin signaling and liver regeneration: circuit, Biology, and opportunities. Gene Expr. *20*, 189–199. https://doi.org/10.3727/105221621X16111780348794.
6. Planas-Paz, L., Orsini, V., Boulter, L., Calabrese, D., Pikiolek, M., Nigsch, F., Xie, Y., Roma, G., Donovan, A., Marti, P., et al. (2016). The RSPO-LGR4/5-ZNRF3/RNF43 module controls liver zonation and size. Nat. Cell Biol. *18*, 467–479. https://doi.org/10.1038/ncb3337.
7. Soto-Gutierrez, A., Gough, A., Vernetti, L.A., Taylor, D.L., and Monga, S.P. (2017). Pre-clinical and clinical investigations of metabolic zonation in liver diseases: the potential of microphysiology systems. Exp. Biol. Med. *242*, 1605–1616. https://doi.org/10.1177/1535370217707731.
8. Ben-Moshe, S., Veg, T., Manco, R., Dan, S., Papinutti, D., Lifshitz, A., Kolodziejczyk, A.A., Bahar Halpern, K., Elinav, E., and Itzkovitz, S. (2022). The spatiotemporal program of zonal liver regeneration following acute injury. Cell Stem Cell *29*, 973–989.e10. https://doi.org/10.1016/j.stem.2022.04.008.
9. Michalopoulos, G.K. (2021). Novel insights into liver homeostasis and regeneration. Nat. Rev. Gastroenterol. Hepatol. *18*, 369–370. https://doi.org/10.1038/s41575-021-00454-0.
10. Michalopoulos, G.K., and Bhushan, B. (2021). Liver regeneration: biological and pathological mechanisms and implications. Nat. Rev. Gastroenterol. Hepatol. *18*, 40–55. https://doi.org/10.1038/s41575-020-0342-4.
11. Sekine, S., Gutiérrez, P.J.A., Lan, B.Y.A., Feng, S., and Hebrok, M. (2007). Liver-specific loss of beta-catenin results in delayed hepatocyte proliferation after partial hepatectomy. Hepatology *45*, 361–368. https://doi.org/10.1002/hep.21523.
12. Sekine, S., Lan, B.Y.A., Bedolli, M., Feng, S., and Hebrok, M. (2006). Liver-specific loss of beta-catenin blocks glutamine synthesis pathway activity and cytochrome p450 expression in mice. Hepatology *43*, 817–825. https://doi.org/10.1002/hep.21131.
13. Tan, X., Behari, J., Cieply, B., Michalopoulos, G.K., and Monga, S.P.S. (2006). Conditional deletion of beta-catenin reveals its role in liver growth and regeneration. Gastroenterology *131*, 1561–1572. https://doi.org/10.1053/j.gastro.2006.08.042.
14. Yang, J., Mowry, L.E., Nejak-Bowen, K.N., Okabe, H., Diegel, C.R., Lang, R.A., Williams, B.O., and Monga, S.P. (2014). beta-catenin signaling in murine liver zonation and regeneration: a Wnt-Wnt situation. Hepatology *60*, 964–976. https://doi.org/10.1002/hep.27082.
15. Leibing, T., Géraud, C., Augustin, I., Boutros, M., Augustin, H.G., Okun, J.G., Langhans, C.D., Zierow, J., Wohlfeil, S.A., Olsavszky, V., et al. (2018). Angiocrine Wnt signaling controls liver growth and metabolic maturation in mice. Hepatology *68*, 707–722. https://doi.org/10.1002/hep.29613.
16. Ma, R., Martínez-Ramírez, A.S., Borders, T.L., Gao, F., and Sosa-Pineda, B. (2020). Metabolic and non-metabolic liver zonation is established non-synchronously and requires sinusoidal Wnts. Elife *9*, e46206. https://doi.org/10.7554/eLife.46206.
17. Preziosi, M., Okabe, H., Poddar, M., Singh, S., and Monga, S.P. (2018). Endothelial Wnts regulate beta-catenin signaling in murine liver zonation and regeneration: a sequel to the Wnt-Wnt situation. Hepatol. Commun. *2*, 845–860. https://doi.org/10.1002/hep4.1196.
18. Zeng, G., Awan, F., Otruba, W., Muller, P., Apte, U., Tan, X., Gandhi, C., Demetris, A.J., and Monga, S.P.S. (2007). Wnt'er in liver: expression of Wnt and frizzled genes in mouse. Hepatology *45*, 195–204. https://doi.org/10.1002/hep.21473.
19. Wang, B., Zhao, L., Fish, M., Logan, C.Y., and Nusse, R. (2015). Self-renewing diploid Axin2(+) cells fuel homeostatic renewal of the liver. Nature *524*, 180–185. https://doi.org/10.1038/nature14863.
20. Ding, B.S., Nolan, D.J., Butler, J.M., James, D., Babazadeh, A.O., Rosenwaks, Z., Mittal, V., Kobayashi, H., Shido, K., Lyden, D., et al. (2010). Inductive angiocrine signals from sinusoidal endothelium are required for

liver regeneration. Nature *468*, 310–315. https://doi.org/10.1038/nature09493.

21. Zhao, L., Jin, Y., Donahue, K., Tsui, M., Fish, M., Logan, C.Y., Wang, B., and Nusse, R. (2019). Tissue repair in the mouse liver following acute carbon tetrachloride depends on injury-induced wnt/beta-catenin signaling. Hepatology *69*, 2623–2635. https://doi.org/10.1002/hep.30563.
22. Alvarado, T.F., Puliga, E., Preziosi, M., Poddar, M., Singh, S., Columbano, A., Nejak-Bowen, K., and Monga, S.P.S. (2016). Thyroid hormone receptor beta agonist induces beta-catenin-dependent hepatocyte proliferation in mice: implications in hepatic regeneration. Gene Expr. *17*, 19–34. https://doi.org/10.3727/105221616X691631.
23. Fanti, M., Singh, S., Ledda-Columbano, G.M., Columbano, A., and Monga, S.P. (2014). Tri-iodothyronine induces hepatocyte proliferation by protein kinase A-dependent beta-catenin activation in rodents. Hepatology *59*, 2309–2320. https://doi.org/10.1002/hep.26775.
24. Tao, Y., Mis, M., Blazer, L., Ustav, M., Jnr, Steinhart, Z., Chidiac, R., Kubarakos, E., O'Brien, S., Wang, X., Jarvik, N., et al. (2019). Tailored tetravalent antibodies potently and specifically activate Wnt/Frizzled pathways in cells, organoids and mice. Elife *8*, e46134. https://doi.org/10.7554/eLife.46134.
25. Guilliams, M., Bonnardel, J., Haest, B., Vanderborght, B., Wagner, C., Remmerie, A., Bujko, A., Martens, L., Thoné, T., Browaeys, R., et al. (2022). Spatial proteogenomics reveals distinct and evolutionarily conserved hepatic macrophage niches. Cell *185*, 379–396.e38. https://doi.org/10.1016/j.cell.2021.12.018.
26. Halpern, K.B., Shenhav, R., Massalha, H., Toth, B., Egozi, A., Massasa, E.E., Medgalia, C., David, E., Giladi, A., Moor, A.E., et al. (2018). Paired-cell sequencing enables spatial gene expression mapping of liver endothelial cells. Nat. Biotechnol. *36*, 962–970. https://doi.org/10.1038/nbt.4231.
27. Su, T., Yang, Y., Lai, S., Jeong, J., Jung, Y., McConnell, M., Utsumi, T., and Iwakiri, Y. (2021). Single-cell transcriptomics reveals zone-specific alterations of liver sinusoidal endothelial cells in cirrhosis. Cell. Mol. Gastroenterol. Hepatol. *11*, 1139–1161. https://doi.org/10.1016/j.jcmgh.2020.12.007.
28. Dobie, R., Wilson-Kanamori, J.R., Henderson, B.E.P., Smith, J.R., Matchett, K.P., Portman, J.R., Wallenborg, K., Picelli, S., Zagorska, A., Pendem, S.V., et al. (2019). Single-cell transcriptomics uncovers zonation of function in the mesenchyme during liver fibrosis. Cell Rep. *29*, 1832–1847.e8. https://doi.org/10.1016/j.celrep.2019.10.024.
29. Rocha, A.S., Vidal, V., Mertz, M., Kendall, T.J., Charlet, A., Okamoto, H., and Schedl, A. (2015). The angiocrine factor Rspondin3 is a key determinant of liver zonation. Cell Rep. *13*, 1757–1764. https://doi.org/10.1016/j.celrep.2015.10.049.
30. Carroll, T.J., Park, J.S., Hayashi, S., Majumdar, A., and McMahon, A.P. (2005). Wnt9b plays a central role in the regulation of mesenchymal to epithelial transitions underlying organogenesis of the mammalian urogenital system. Dev. Cell *9*, 283–292. https://doi.org/10.1016/j.devcel.2005.05.016.
31. Wei, Y., Wang, Y.G., Jia, Y., Li, L., Yoon, J., Zhang, S., Wang, Z., Zhang, Y., Zhu, M., Sharma, T., et al. (2021). Liver homeostasis is maintained by midlobular zone 2 hepatocytes. Science *371*, eabb1625. https://doi.org/10.1126/science.abb1625.
32. Renard, C.A., Labalette, C., Armengol, C., Cougot, D., Wei, Y., Cairo, S., Pineau, P., Neuveut, C., de Reyniès, A., Dejean, A., et al. (2007). Tbx3 is a downstream target of the Wnt/beta-catenin pathway and a critical mediator of beta-catenin survival functions in liver cancer. Cancer Res. *67*, 901–910. https://doi.org/10.1158/0008-5472.CAN-06-2344.
33. Braeuning, A., and Schwarz, M. (2010). Zonation of heme synthesis enzymes in mouse liver and their regulation by beta-catenin and Ha-ras. Biol. Chem. *391*, 1305–1313. https://doi.org/10.1515/BC.2010.115.
34. Halpern, K.B., Shenhav, R., Matcovitch-Natan, O., Tóth, B., Lemze, D., Golan, M., Massasa, E.E., Baydatch, S., Landen, S., Moor, A.E., et al. (2017). Erratum: single-cell spatial reconstruction reveals global division of labour in the mammalian liver. Nature *543*, 742. https://doi.org/10.1038/nature21729.
35. Janda, C.Y., Dang, L.T., You, C., Chang, J., de Lau, W., Zhong, Z.A., Yan, K.S., Marecic, O., Siepe, D., Li, X., et al. (2017). Surrogate Wnt agonists that phenocopy canonical Wnt and beta-catenin signalling. Nature *545*, 234–237. https://doi.org/10.1038/nature22306.
36. Lee, W.M. (2017). Acetaminophen (APAP) hepatotoxicity-Isn't it time for APAP to go away? J. Hepatol. *67*, 1324–1331. https://doi.org/10.1016/j.jhep.2017.07.005.
37. Ershad, M., Naji, A., and Vearrier, D. (2022). N Acetylcysteine (StatPearls).
38. Bhushan, B., Walesky, C., Manley, M., Gallagher, T., Borude, P., Edwards, G., Monga, S.P.S., and Apte, U. (2014). Pro-regenerative signaling after acetaminophen-induced acute liver injury in mice identified using a novel incremental dose model. Am. J. Pathol. *184*, 3013–3025. https://doi.org/10.1016/j.ajpath.2014.07.019.
39. Jaeschke, H., and Ramachandran, A. (2020). Mechanisms and pathophysiological significance of sterile inflammation during acetaminophen hepatotoxicity. Food Chem. Toxicol. *138*, 111240. https://doi.org/10.1016/j.fct.2020.111240.
40. Yoon, E., Babar, A., Choudhary, M., Kutner, M., and Pyrsopoulos, N. (2016). Acetaminophen-induced hepatotoxicity: a comprehensive update. J. Clin. Transl. Hepatol. *4*, 131–142. https://doi.org/10.14218/JCTH.2015.00052.
41. Okabe, H., Yang, J., Sylakowski, K., Yovchev, M., Miyagawa, Y., Nagarajan, S., Chikina, M., Thompson, M., Oertel, M., Baba, H., et al. (2016). Wnt signaling regulates hepatobiliary repair following cholestatic liver injury in mice. Hepatology *64*, 1652–1666. https://doi.org/10.1002/hep.28774.
42. Adebayo Michael, A.O., Ko, S., Tao, J., Moghe, A., Yang, H., Xu, M., Russell, J.O., Pradhan-Sundd, T., Liu, S., Singh, S., et al. (2019). Inhibiting glutamine-dependent mTORC1 activation ameliorates liver cancers driven by beta-catenin mutations. Cell Metab. *29*, 1135–1150.e6. https://doi.org/10.1016/j.cmet.2019.01.002.
43. Guilliams, M., Bonnardel, J., Haest, B., Vanderborght, B., Wagner, C., Remmerie, A., Bujko, A., Martens, L., Thoné, T., Browaeys, R., et al. (2022). Spatial proteogenomics reveals distinct and evolutionarily conserved hepatic macrophage niches. Cell *185*, 379–396.e38. https://doi.org/10.1016/j.cell.2021.12.018.
44. Apte, U., Singh, S., Zeng, G., Cieply, B., Virji, M.A., Wu, T., and Monga, S.P.S. (2009). Beta-catenin activation promotes liver regeneration after acetaminophen-induced injury. Am. J. Pathol. *175*, 1056–1065. https://doi.org/10.2353/ajpath.2009.080976.
45. Colletti, M., Cicchini, C., Conigliaro, A., Santangelo, L., Alonzi, T., Pasquini, E., Tripodi, M., and Amicone, L. (2009). Convergence of Wnt signaling on the HNF4alpha-driven transcription in controlling liver zonation. Gastroenterology *137*, 660–672. https://doi.org/10.1053/j.gastro.2009.05.038.
46. Stanulović, V.S., Kyrmizi, I., Kruithof-de Julio, M., Hoogenkamp, M., Vermeulen, J.L.M., Ruijter, J.M., Talianidis, I., Hakvoort, T.B.M., and Lamers, W.H. (2007). Hepatic HNF4alpha deficiency induces periportal expression of glutamine synthetase and other pericentral enzymes. Hepatology *45*, 433–444. https://doi.org/10.1002/hep.21456.
47. Berasain, C., and Avila, M.A. (2014). Deciphering liver zonation: new insights into the beta-catenin, Tcf4, and HNF4alpha triad. Hepatology *59*, 2080–2082. https://doi.org/10.1002/hep.27000.
48. Gougelet, A., Torre, C., Veber, P., Sartor, C., Bachelot, L., Denechaud, P.D., Godard, C., Moldes, M., Burnol, A.F., Dubuquoy, C., et al. (2014). T-cell factor 4 and beta-catenin chromatin occupancies pattern zonal liver metabolism in mice. Hepatology *59*, 2344–2357. https://doi.org/10.1002/hep.26924.
49. Zhang, Z., Broderick, C., Nishimoto, M., Yamaguchi, T., Lee, S.J., Zhang, H., Chen, H., Patel, M., Ye, J., Ponce, A., et al. (2020). Tissue-targeted

R-spondin mimetics for liver regeneration. Sci. Rep. *10*, 13951. https://doi.org/10.1038/s41598-020-70912-3.

50. Chidiac, R., Abedin, M., Macleod, G., Yang, A., Thibeault, P.E., Blazer, L.L., Adams, J.J., Zhang, L., Roehrich, H., Jo, H.N., et al. (2021). A Norrin/Wnt surrogate antibody stimulates endothelial cell barrier function and rescues retinopathy. EMBO Mol. Med. *13*, e13977. https://doi.org/10.15252/emmm.202113977.

51. Smilkstein, M.J., Knapp, G.L., Kulig, K.W., and Rumack, B.H. (1988). Efficacy of oral N-acetylcysteine in the treatment of acetaminophen overdose. Analysis of the national multicenter study (1976 to 1985). N. Engl. J. Med. *319*, 1557–1562. https://doi.org/10.1056/NEJM198812153192401.

52. Pelletier, S., Gingras, S., and Green, D.R. (2015). Mouse genome engineering via CRISPR-Cas9 for study of immune function. Immunity *42*, 18–27. https://doi.org/10.1016/j.immuni.2015.01.004.

53. Schneider, C.A., Rasband, W.S., and Eliceiri, K.W. (2012). NIH Image to ImageJ: 25 years of image analysis. Nat. Methods *9*, 671–675. https://doi.org/10.1038/nmeth.2089.

54. Bankhead, P., Loughrey, M.B., Fernández, J.A., Dombrowski, Y., McArt, D.G., Dunne, P.D., McQuaid, S., Gray, R.T., Murray, L.J., Coleman, H.G., et al. (2017). QuPath: open source software for digital pathology image analysis. Sci. Rep. *7*, 16878. https://doi.org/10.1038/s41598-017-17204-5.

55. Hao, Y., Hao, S., Andersen-Nissen, E., Mauck, W.M., 3rd, Zheng, S., Butler, A., Lee, M.J., Wilk, A.J., Darby, C., Zager, M., et al. (2021). Integrated analysis of multimodal single-cell data. Cell *184*, 3573–3587.e29. https://doi.org/10.1016/j.cell.2021.04.048.

56. Xiang, R., Wang, W., Yang, L., Wang, S., Xu, C., and Chen, X. (2021). A comparison for dimensionality reduction methods of single-cell RNA-seq data. Front. Genet. *12*, 646936. https://doi.org/10.3389/fgene.2021.646936.

57. Frankish, A., Diekhans, M., Ferreira, A.-M., Johnson, R., Jungreis, I., Loveland, J., et al. (2018). GENCODE reference annotation for the human and mouse genomes. Nucleic Acids Res *47*, D766–D773.

58. Yates, A.D., Achuthan, P., Akanni, W., Allen, J., Allen, J., Alvarez-Jarreta, J., et al. (2019). Ensembl 2020. Nucleic Acids Res *48*, D682–D688.

59. Marçais, G., and Kingsford, C. (2011). A fast, lock-free approach for efficient parallel counting of occurrences of k-mers. Bioinformatics *27*, 764–770.

60. Gans, J.D., and Wolinsky, M. (2008). Improved assay-dependent searching of nucleic acid sequence databases. Nucleic Acids Res *36*. e74–e74.

61. Rodriguez, J.M., Rodriguez-Rivas, J., Di Domenico, T., Vázquez, J., Valencia, A., and Tress, M.L. (2018). APPRIS 2017: principal isoforms for multiple gene sets. Nucleic Acids Res *46*, D213–D217.

CellPress
OPEN ACCESS

STAR★METHODS

KEY RESOURCES TABLE

REAGENT or RESOURCE	SOURCE	IDENTIFIER
Antibodies		
Rabbit polyclonal anti-GS	Sigma-Aldrich	Cat#G2781; RRID: AB_259853
Rabbit polyclonal anti-CYP2E1	Sigma-Aldrich	Cat#HPA009128; RRID: AB_1078613
Mouse monoclonal anti-CYP1A2	Santa Cruz Biotechnology	Cat#sc-53241; RRID: AB_629359
Rabbit monoclonal anti-Cyclin D1	Abcam	Cat#ab134175; RRID: AB_2750906
Rat monoclonal anti-CD45	Santa Cruz Biotechnology	Cat#sc-53665; RRID: AB_629093
Rabbit monoclonal anti-Ki67	Cell Signaling Technology	Cat#12202S
Rat monoclonal anti-BrdU	Accurate Chemicals	Cat#OBT0030A
Mouse monoclonal anti-CYP2F2	Santa Cruz Biotechnology	Cat#sc-374540; RRID: AB_10987684
Rabbit monoclonal anti-GFP	Cell Signaling Technology	Cat#2956S
Mouse monoclonal anti-RGN	Santa Cruz Biotechnology	Cat#sc-390098
Goat polyclonal anti-PIGR	R and D Systems	Cat#AF2800; RRID: AB_2283871
Rat monoclonal anti-CK-19	DSHB	Cat#TROMA-III; RRID: AB_2133570
Goat polyclonal anti-CD31	R and D Systems	Cat#AF3628; RRID: AB_2161028
Bacterial and virus strains		
AAV.TBG.PI.Cre.rBG	addgene	Cat#107787-AAV8
Chemicals, peptides, and recombinant proteins		
Control IgG	Clevers et al.[1]	N/A
FL6.13	Clevers et al.[1]	N/A
Acetaminophen	Sigma-Aldrich	Cat#A7085
5-Bromo-2′-deoxyuridine (BrdU)	Sigma-Aldrich	Cat#B5002
Critical commercial assays		
TRIzol^{TM}	Thermo Scientific	Cat#15596026
RNeasy Mini Kit	Qiagen	Cat#74104
Power SYBR® Green PCR Master Mix	Applied Biosystems	Cat#4367660
Nonfat dry milk	Cell Signaling Technology	Cat#9999
SuperSignal® West Pico Chemiluminescent Substrate	Thermo Scientific	Cat#34080
VECTASTAIN® Elite® ABC-HRP Kit, Peroxidase	Vector Laboratories	Cat#PK-6101
DAB Substrate Kit, Peroxidase (HRP)	Vector Laboratories	Cat#SK-4100
Deposited data		
Raw and processed spatial single-cell data	This paper	GSE199463
Experimental models: Organisms/strains		
Mouse: C57BL/6J	The Jackson Laboratories	JAX: 000,664; RRID: IMSR_JAX:000,664
Mouse: B6; 129P2-Lyve1$^{tm1.1(EGFP/cre)Cys}$/J	The Jackson Laboratories	JAX: 012,601; RRID: IMSR_JAX:012,601
Mouse: B6.129X1-Gt(ROSA)26Sor$^{tm1(EYFP)Cos}$/J	The Jackson Laboratories	JAX: 006,148; RRID: IMSR_JAX:006,148
Mouse: Wnt9b$^{tm1.2Amc}$/J	The Jackson Laboratories	JAX: 008,469; RRID: IMSR_JAX:008,469
Mouse: Wnt2$^{flox/flox}$	This paper	N/A
Mouse: EC-Wnt9b-KO on C57BL/6J	This paper	N/A
Mouse: EC-Wnt2-KO on C57BL/6J	This paper	N/A
Mouse: EC-Wnt2-9b-DKO on C57BL/6J	This paper	N/A
Mouse: EC-Wls-KO on C57BL/6J	Russell and Monga[2]	N/A
Mouse: Lrp5$^{flox/flox}$-Lrp6$^{flox/flox}$	Burke et al.[3]	N/A

(*Continued on next page*)

Continued		
REAGENT or RESOURCE	SOURCE	IDENTIFIER
Oligonucleotides		
Primers for genotyping	This paper	Table S1
Sizes for genotyping PCR product	This paper	Table S2
Probes for Molecular Cartography	This paper	Table S3
Primers for qPCR	This paper	Table S4
Software and algorithms		
ImageJ	Benhamouche et al.[4]	https://imagej.nih.gov/ij/
QuPath	Hu and Monga[5]	https://qupath.readthedocs.io/en/stable/
Seurat	Planas-Paz et al.[6]	https://doi.org/10.1016/j.cell.2021.04.048
Principal component analysis (PCA) and uniform manifold approximation and projection (UMAP)	Soto-Gutierrez et al.[7]	https://doi.org/10.3389/fgene.2021.646936

RESOURCE AVAILABILITY

Lead contact

Further information and requests for resources and reagents should be directed to and will be fulfilled by the lead contact, Satdarshan P. Monga (smonga@pitt.edu).

Materials availability

Mouse lines and all unique reagents used in this study are available from the lead contact with a completed Material Transfer Agreement.

Data and code availability

Spatial single-cell data has been deposited into NCBI GEO database with accession ID: GSE199463. Raw and processed data can be downloaded by https://www.ncbi.nlm.nih.gov/geo/query/acc.cgi?acc=GSE199463. The software and algorithms for data analyses used in this study are published and referenced throughout the STAR Methods section. Any additional information required to reanalyze the data reported in this paper is available from the lead contact upon request.

EXPERIMENTAL MODEL AND SUBJECT DETAILS

Animals

All animal husbandry and experimental procedures, including animal housing and diet, were performed under the guidelines and approval of the National Institutes of Health and the Institutional Animal Care and Use Committee at the University of Pittsburgh. Mice were fed regular chow in standard caging and kept under a 12-h light–dark cycle with no enrichment. Lyve1-cre mice were purchased from Jackson Laboratories. *Wnt9b*$^{flox/flox}$ mice were reported before.[30] *Wnt2* exon-2-floxed mice were generated by insertion of *LoxP* sites flanking exon 2, through use of CRISPR/Cas9 technology as described elsewhere.[52] Briefly, using single-guide RNAs (sgRNAs), double-strand breaks flanking exon 2 were targeted to optimal CAS9 target sites within introns 1 and 2. A synthetic floxed allele was generated as a single-stranded-oligodeoxynucleotide (ssODN) template for homology-directed repair (HDR). The HDR template was a megamer, 1166bp ssDNA oligo (Integrated DNA Technologies), encoding exon 2 flanked by *LoxP* sites with adjacent EcoR1 sites for genotyping and 5′ AND-3′ homology arms homologous to the genomic sequences up-and downstream of the 5′ AND-3′ CAS9 cut sites in Introns 1 and 2. Homologous recombination was achieved by microinjection of a mixture of Cas9 protein (0.3 μM), I1 & I2 sgRNAs (21.23 ng/μL each) and the ssODN (10 ng/μL) into the pronuclei of fertilized embryos (C57BL/6J, The Jackson Laboratory). The injected zygotes were cultured overnight, the next day the embryos that developed to the 2-cell stage were transferred to the oviducts of pseudopregnant CD1 female recipients. Offspring were genotyped by PCR and RFLP analysis to confirm insertion of 5′and 3′ *loxP* sites. Founder mice, M14 and M15, were back-crossed with C57BL/6J wildtype (WT) mice for two generations and selected for the presence of *Wnt2*$^{flox/flox}$ allele to generate N2 mice, which were then bred with *Wnt9b*$^{flox/flox}$, Rosa-stop$^{flox/flox}$-EYFP, and Lyve1-cre$^{+/-}$ mice. Genotyping primers and PCR products are listed in Tables S1 and S2.

Lyve1-cre$^{+/-}$; *Wnt2*$^{flox/-}$ *Wnt9b*$^{flox/flox}$ Rosa-stop$^{flox/flox}$-EYFP mice are hereafter referred to as EC-Wnt9b-KO. Lyve1-cre$^{+/-}$; *Wnt2*$^{flox/flox}$ *Wnt9b*$^{flox/-}$ Rosa-stop$^{flox/flox}$-EYFP mice are hereafter referred to as EC-Wnt2-KO. Lyve1-cre$^{+/-}$; *Wnt2*$^{flox/flox}$ *Wnt9b*$^{flox/flox}$ Rosa-stop$^{flox/flox}$-EYFP mice are hereafter referred to as EC-Wnt2-9b-DKO. The rest of mice were used as littermate controls (Control). EC-Wls-KO mice were described before.[17] LRP5-6-LDKO mice were generated by i.p. injection of 1 x 10^{12} genome copies of AAV8-TBG-Cre (Addgene, 107,787-AAV8) per mice to LRP5-6 double floxed mice described before.[14]

METHOD DETAILS

Two-third or partial hepatectomy

Two to three-month-old male and female Control, EC-Wnt2-KO, EC-Wnt9b-KO, EC-Wnt2-9b-DKO mice were subjected to PH as describe before.[13,14,17] Animals were sacrificed at 40 h post-PH. Serum and liver tissues were harvested for further analysis.

FL6.13 treatment

Two to three-month-old male wildtype, EC-Wnt2-9b-DKO, EC-Wls-KO, LRP5-6-LDKO mice were treated with 5 mg/kg control IgG or FL6.13 every other day as described elsewhere,[24,50] for one week followed by PH. Animals were sacrificed at 24 h post-PH. 1 mg/mL BrdU was given in drinking water. Serum and liver tissues were harvested for further analysis.

Acetaminophen study

8-week-old male C57BL/6 mice were ordered from Jackson lab. Mice were given food and water from 6 p.m. to 9 p.m. in dark room and then fasted from 9 p.m. to 9 a.m. next day. 600 mg/kg APAP was intraperitoneal (i.p.) injected, and food were given back. 12 h or 32 h post APAP injection, mice were randomly grouped and were i.p. injected with 5 mg/kg control IgG or FL6.13 in 0.9% saline. Mice were sacrificed at 48 h or 60 h. Serum and liver tissues were collected for further analysis.

scRNA sequencing data analysis

scRNA sequencing data were published before and were analyzed at: https://www.livercellatlas.org/index.php.[25]

Molecular cartography™

Detailed protocol of tissue processing, probe design, imaging, signal segmentation and barcoding was discussed before.[25] Probes used in this study are listed in Table S3. One animal per group was used. Molecular Cartography images were performed in ImageJ using genexyz Polylux tool plugin from Resolve BioSciences to examine specific Molecular Cartography signals.[53]

Single-cell spatial transcriptomic analysis was performed according to the workflow in the Figures S10 and S13. Two bioinformatic pipelines were applied to study the data. For the first pipeline, gene counts were quantified per cell based on the cell identification from QuPath software.[54] Slide control, slide EC-Wnt2-9b-DKO, and slide FL6.13 treatment were integrated by R package *Seurat*.[55] For quality control, cells with less than 10 gene-count were filtered out. Non-hepatocyte cells were defined by the cells with any expression of the five non-hepatocyte makers (*Lrat*, *Pecam1*, *Ptprc*, *Lyz2*, and *Adgre1*) and were removed from further analysis when comparing control and FL6.13 treatment (Figure S13). After pre-processing, dimension reduction method principal component analysis (PCA) and uniform manifold approximation and projection (UMAP)[56] were performed on hepatocytes based on 16 zonated markers (pericentral markers: *Cldn2*, *Lect2*, *Glul*, *Oat*, *Cyp1a2*, *Cyp2e1*, *Gstm1*, *Rgn*; midzonal markers: *Pon1*; periportal markers: *Gls2*, *G6pc*, *Fbp1*, *Hsd17b13*, *Vtn*, *Cyp2f2*, *Pigr*). Eventually, cells were grouped into distinct clusters and annotated by the zonated genes. Feature plots and violin plots were generated by *Seurat* to visualize gene expression at cell-level and cluster-level.

For the second pipeline, a line was drawn from center of central vein (marking pericentral region) to portal vein (marking periportal region) based on respective landmark genes (*Cyp2e1*, *Glul*, and *Sox9*). Then an upper-bound and a lower-bound line were drawn parallel to the central line with 500-pixel extension. Bounded by these two lines, perpendicular lines were drawn to separate the region between pericentral and periportal into 9 equal segments. In total, 7, 9, and 9 pericentral-to-periportal regions were identified in slide control, slide EC-Wnt2-9b-DKO, and slide FL6.13 treatment, respectively. Gene counts and expression density (gene counts per area) were quantified within each segment and averaged across the defined pericentral-to-periportal regions. Cells located in these 9-segments were also identified based on their X, Y positions in the slides and traced back to the UMAPs to show the histological location of cells.

Tissue sections

Mouse liver samples were frozen in liquid nitrogen as recommended by the Molecular Cartography protocol. Frozen samples were sectioned with a cryostat (Leica, CM 1850-3-1) and 10μm thick sections were placed within the capture areas of cold Resolve Biosciences slides. Samples were then sent to Resolve BioSciences on dry ice for analysis. Upon arrival, tissue sections were thawed and fixed according to Molecular Cartography protocol with MF1 for 30 min at 4°C. After fixation, sections were washed twice in 1x PBS for two min, followed by one min washes in 70% Ethanol at room temperature. Fixed samples were undergoing an alcoholic series starting with an incubation in isopropanol for 1 min, followed by 95% and 75% ethanol. The samples were used for Molecular Cartography™ (100-plex combinatorial single molecule fluorescence in-situ hybridization) according to the manufacturer's instructions *Day 1: Molecular Preparation Protocol* for mouse or human liver, starting with the aspiration of ethanol and the addition of buffer DST3 followed by tissue priming and hybridization. Briefly, tissues were primed for 30 minutes at 37°C followed by overnight hybridization of all probes specific for the target genes (see below for probe design details and target list). Samples were washed the next day to remove excess probes and fluorescently tagged in a two-step color development process. Regions of interest were imaged as described below and fluorescent signals removed during decolorization. Color development, imaging and decolorization were repeated for multiple cycles to build a unique combinatorial code for every target gene that was derived from raw images as described below.

Probe design

The probes for 100 genes were designed using Resolve's proprietary design algorithm. Briefly, the probe-design was performed at the gene-level. For every targeted gene all full-length protein coding transcript sequences from the ENSEMBL database were used as design targets if the isoform had the GENCODE annotation tag 'basic'.[57,58] To speed up the process, the calculation of computationally expensive parts, especially the off-target searches, the selection of probe sequences was not performed randomly, but limited to sequences with high success rates. To filter highly repetitive regions, the abundance of k-mers was obtained from the background transcriptome using Jellyfish.[59] Every target sequence was scanned once for all k-mers, and those regions with rare k-mers were preferred as seeds for full probe design. A probe candidate was generated by extending a seed sequence until a certain target stability was reached. A set of simple rules was applied to discard sequences that were found experimentally to cause problems. After these fast screens, every kept probe candidate was mapped to the background transcriptome using ThermonucleotideBLAST[60] and probes with stable off-target hits were discarded. Specific probes were then scored based on the number of on-target matches (isoforms), which were weighted by their associated APPRIS level,[61] favoring principal isoforms over others. A bonus was added if the binding-site was inside the protein-coding region. From the pool of accepted probes, the final set was composed by greedily picking the highest scoring probes. The gene names and specific probes designed by Resolve BioSciences are included in Table S3.

Imaging

Regions of interests were identified by us. Based on these, samples were imaged on a Zeiss Celldiscoverer 7, using the 50x Plan Apochromat water immersion objective with an NA of 1.2 and the 0.5x magnification changer, resulting in a 25x final magnification. Standard CD7 LED excitation light source, filters, and dichroic mirrors were used together with customized emission filters optimized for detecting specific signals. Excitation time per image was 1000 ms for each channel (DAPI was 20 ms). A z-stack was taken at each region with a distance per z-slice according to the Nyquist-Shannon sampling theorem. The custom CD7 CMOS camera (Zeiss Axiocam Mono 712, 3.45 μm pixel size) was used. For each region, a z-stack per fluorescent color (two colors) was imaged per imaging round. A total of 8 imaging rounds were done for each position, resulting in 16 z-stacks per region. The completely automated imaging process per round (including water immersion generation and precise relocation of regions to image in all three dimensions) was realized by a custom python script using the scripting API of the Zeiss ZEN software (Open application development).

Spot segmentation

The algorithms for spot segmentation were written in Java and are based on the ImageJ library functionalities. Only the iterative closest point algorithm is written in C++ based on the libpointmatcher library (https://github.com/ethz-asl/libpointmatcher).

Preprocessing

As a first step all images were corrected for background fluorescence. A target value for the allowed number of maxima was determined based upon the area of the slice in μm^2 multiplied by the factor 0.5. This factor was empirically optimized. The brightest maxima per plane were determined, based upon an empirically optimized threshold. The number and location of the respective maxima was stored. This procedure was done for every image slice independently. Maxima that did not have a neighboring maximum in an adjacent slice (called z-group) were excluded. The resulting maxima list was further filtered in an iterative loop by adjusting the allowed thresholds for (Babs-Bback) and (Bperi-Bback) to reach a feature target value (Babs: absolute brightness, Bback: local background, Bperi: background of periphery within 1 pixel). This feature target values were based upon the volume of the 3D-image. Only maxima still in a zgroup of at least 2 after filtering were passing the filter step. Each z-group was counted as one hit. The members of the z-groups with the highest absolute brightness were used as features and written to a file. They resemble a 3D-point cloud. Final signal segmentation and decoding: To align the raw data images from different imaging rounds, images had to be corrected. To do so the extracted feature point clouds were used to find the transformation matrices. For this purpose, an iterative closest point cloud algorithm was used to minimize the error between two point-clouds. The point clouds of each round were aligned to the point cloud of round one (reference point cloud). The corresponding point clouds were stored for downstream processes. Based upon the transformation matrices the corresponding images were processed by a rigid transformation using trilinear interpolation. The aligned images were used to create a profile for each pixel consisting of 16 values (16 images from two color channels in 8 imaging rounds). The pixel profiles were filtered for variance from zero normalized by total brightness of all pixels in the profile. Matched pixel profiles with the highest score were assigned as an ID to the pixel. Pixels with neighbors having the same ID were grouped. The pixel groups were filtered by group size, number of direct adjacent pixels in group, number of dimensions with size of two pixels. The local 3D-maxima of the groups were determined as potential final transcript locations. Maxima were filtered by number of maxima in the raw data images where a maximum was expected. Remaining maxima were further evaluated by the fit to the corresponding code. The remaining maxima were written to the results file and considered to resemble transcripts of the corresponding gene. The ratio of signals matching to codes used in the experiment and signals matching to codes not used in the experiment were used as estimation for specificity (false positives).

Downstream analysis

Final image analysis was performed in ImageJ using the Polylux tool plugin from Resolve BioSciences to examine specific Molecular Cartography™ signals.

RNA isolation and qPCR

Whole liver was homogenized in TRIzol (Thermo Scientific, 15,596,026) and nucleic acid was isolated through phenol-chloroform extraction. RNA was reverse transcribed into cDNA using SuperScript III (Invitrogen, 18,080-044). Real-time PCR was performed

in technical duplicate on a StepOnePlus Real-Time PCR System (Applied Biosystems, 4,376,600) using the Power SYBR Green PCR Master Mix (Applied Biosystems, 4,367,660). Target gene expression was normalized to housekeeping genes *Rn18s*, and fold change was calculated utilizing the ΔΔ-Ct method. Primers are listed in Table S4.

Protein isolation and western blot

Snap frozen liver samples were homogenized in RIPA buffer with fresh proteinase and phosphatase inhibitor. The concentration of the protein was determined by the bicinchoninic acid assay. Protein sample was prepared with loading buffer (Bio-Rad, 1,610,737) with 5% 2-Mercaptoethanol (Bio-Rad, 161-0710) and subjected to electrophoresis. Protein sample was separated on pre-cast 7.5% or 4-20% polyacrylamide gels (Bio-Rad) and transferred to the PVDF membrane using the Trans-Blot Turbo Transfer System (Bio-Rad). Membranes were stained with Ponceau-S and blocked for 30 min with 5% nonfat dry milk (Cell signaling, 9999) or 5% BSA in Blotto buffer (0.15M NaCl, 0.02M Tris pH 7.5, 0.1% Tween in dH2O), and incubated with primary antibodies at 4°C overnight at the following concentrations: GS (Sigma, G2781, 1:2000), CYP2E1 (Sigma, HPA009128, 1:1000), CYP1A2 (Santa Cruz Biotechnology, sc-53241, 1:1000), Cyclin D1 (Abcam, ab134175, 1:1000). Membranes were washed in Blotto buffer and incubated with the appropriate HRP-conjugated secondary antibody for 60 min at room temperature. Membranes were washed with Blotto buffer, and bands were developed utilizing SuperSignal West Pico Chemiluminescent Substrate (Thermo Scientific, 34,080) and visualized by time-gradient autoradiography.

Immunohistochemistry

Livers were fixed in 10% buffered formalin for 48-72 h prior to paraffin embedding. Blocks were cut into 4μm sections, deparaffinized, and washed with PBS. For antigen retrieval, samples were microwaved for 12 min in pH = 6 sodium citrate buffer (CD45), or in pH = 9 Tris-EDTA buffer (BrdU), or were pressure cooked for 20 min in pH = 6 sodium citrate buffer (CYP2E1, CYP1A2, Ki67, CYP2F2), or in pH = 9 Tris-EDTA buffer (GFP, Cyclin D1, RGN, PIGR). For BrdU, slides were then incubated with 2M HCl for 1 h at room temperature and washed with 0.5M Borax for 5 min. GS staining doesn't need antigen retrieval. Samples were then placed in 3% H_2O_2 for 10 min to quench endogenous peroxide activity. After washing with PBS, slides were blocked for 10 min. The primary antibodies were incubated at the following concentrations in PBS: CD45 (Santa Cruz Biotechnology, sc-53665, 1:100), BrdU (Accurate Chemicals, OBT0030A, 1:75), GS (Sigma, G2781, 1:3000), CYP2E1 (Sigma, HPA009128, 1:100), CYP1A2 (Santa Cruz Biotechnology, sc-53241, 1:100), Ki67 (Cell signaling, 12,202, 1:500), CYP2F2 (Santa Cruz Biotechnology, sc-374540, 1:100), GFP (Cell signaling, 2956, 1:100), Cyclin D1 (Abcam, ab134175, 1:200), RGN (Santa Cruz Biotechnology, sc-390098, 1:100), PIGR (R&D Systems, AF2800, 1:100) for 1 h at room temperature. Samples were washed with PBS three times and incubated with the appropriate biotinylated secondary antibody (Vector Laboratories) diluted 1:250 in antibody diluent for 15 min at room temperature. Samples were washed with PBS three times and sensitized with the Vectastain ABC kit (Vector Laboratories, PK-6101). Following three washes with PBS color was developed with DAB Peroxidase Substrate Kit (Vector Laboratories, SK-4100), followed by quenching in distilled water. Slides were counterstained with hematoxylin (Thermo Scientific, 7211), dehydrated to xylene and coverslips applied with Cytoseal XYL (Thermo Scientific, 8312-4). Images were taken on a Zeiss Axioskop 40 inverted brightfield microscope.

Immunofluorescence

For triple staining of CK19, CD31, and GFP, paraffin sections were deparaffinized and pressure cooked in pH = 9 Tris-EDTA buffer for 20min. Slides were permeabilized with 0.3% Triton X-100 in PBS for 20 min at room temperature and then blocked with 5% normal donkey serum in 0.3% Triton X-100 in PBS (antibody diluent) for 30 min at room temperature. Antibodies were diluted as follows: CK19 (DSHB, TROMA-III, 1:10), CD31 (R&D Systems, AF3628, 1:100), GFP (Cell signaling, 2956, 1:100), in antibody diluent and incubated at 4°C overnight. Samples were washed three times in 0.1% Triton X-100 in PBS (wash solution) and incubated with the proper fluorescent secondary antibody (Alexa Fluor 488/555/647, Invitrogen) diluted 1:300 in antibody diluent for 2 h at room temperature. Samples were washed three times and incubated with DAPI (Sigma, B2883) for 1 min. Samples were washed three times and mounted with gelvator. Images were taken on a Nikon Eclipse Ti epifluorescence microscope or a Zeiss LSM700 confocal microscope and were analyzed with ImageJ.

QUANTIFICATION AND STATISTICAL ANALYSIS

Statistical comparison between two groups was done with the unpaired Student's *t* test. Multiple-group comparison was done with one-way ANOVA. The statistical details for each experiment can be found in the figure legends. GraphPad Prism version 9.0 was used for graph generation, and a p value of less than 0.05 was considered significant. The bars represent means ± SEM ns = not significant, $^{*}p < 0.05$, $^{**}p < 0.01$, $^{***}p < 0.001$, and $^{****}p < 0.0001$.

Cell Reports Medicine, Volume *3*

Supplemental information

Single-cell spatial transcriptomics reveals a dynamic control of metabolic zonation and liver regeneration by endothelial cell Wnt2 and Wnt9b

Shikai Hu, Silvia Liu, Yu Bian, Minakshi Poddar, Sucha Singh, Catherine Cao, Jackson McGaughey, Aaron Bell, Levi L. Blazer, Jarret J. Adams, Sachdev S. Sidhu, Stephane Angers, and Satdarshan P. Monga

Supplementary Figures and Figure Legends

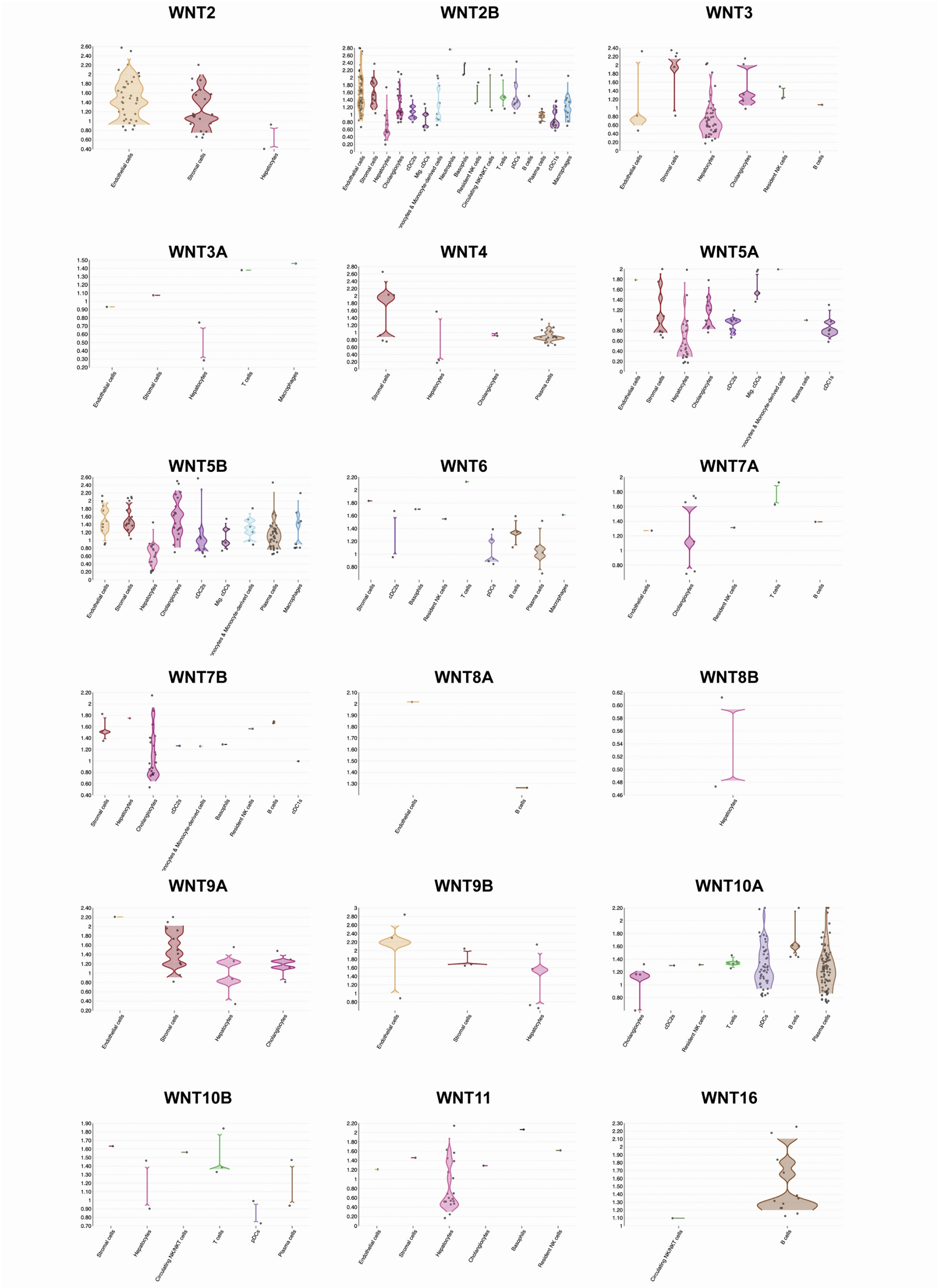

Fig. S1: Violin plots showing expression level of WNTs in normal human liver. (Related to Figure 1)
Eighteen WNTs were detected by scRNA seq. ECs were the predominant source of WNT2, WNT2B, and WNT9B.

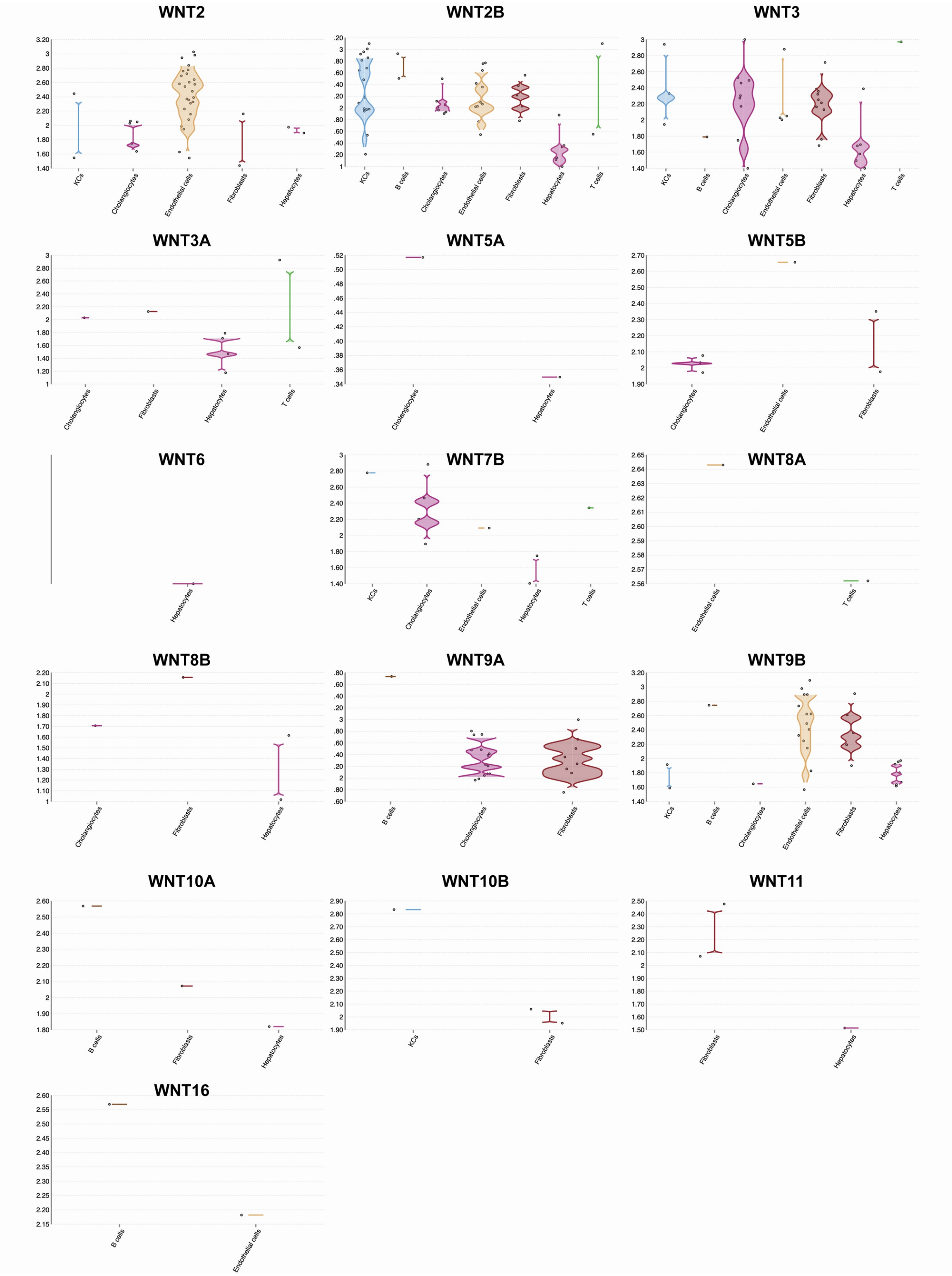

Fig. S2: Violin plots showing expression level of WNTs in monkey liver. (Related to Figure 1)

Sixteen WNTs were detected. ECs expressed high levels of WNT2 and WNT9B.

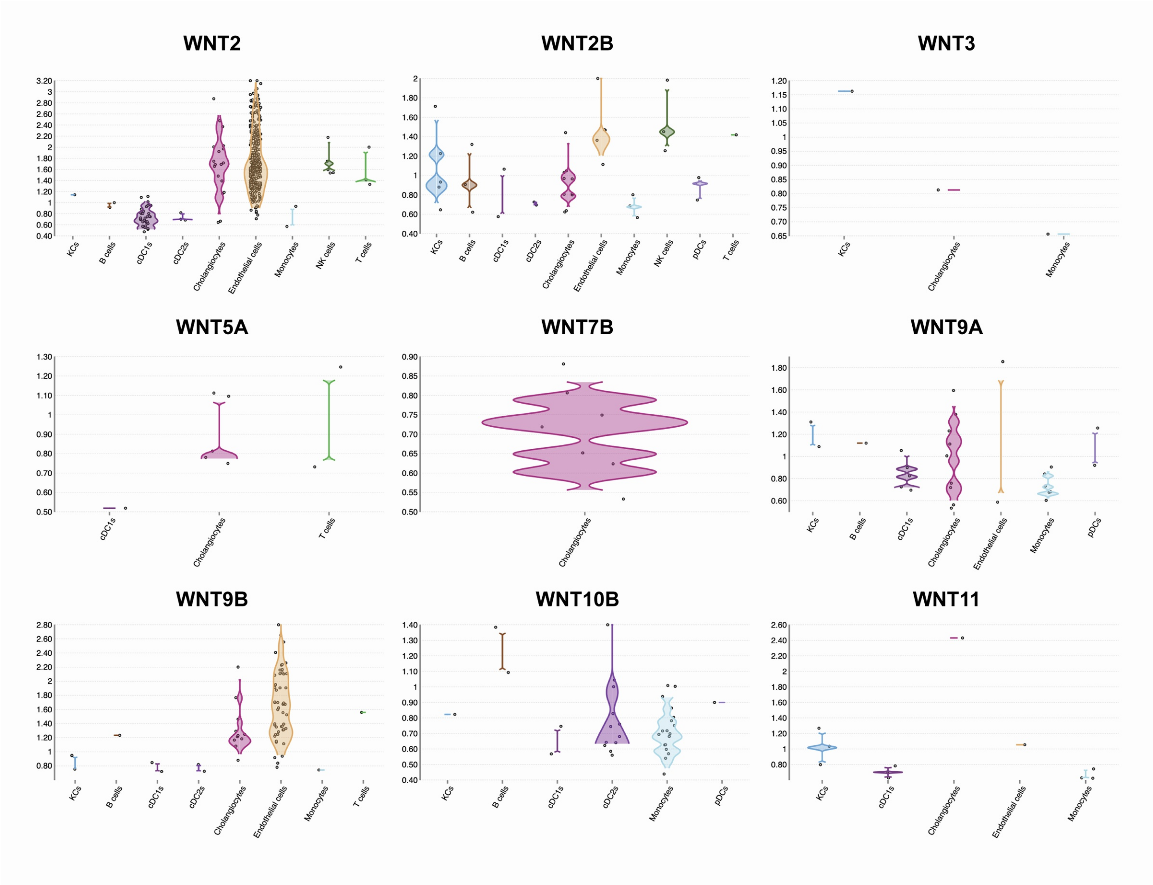

Fig. S3: Violin plots showing expression level of WNTs in pig liver. (Related to Figure 1)

Nine WNTs were detected. ECs expressed high levels of WNT2 and WNT9B.

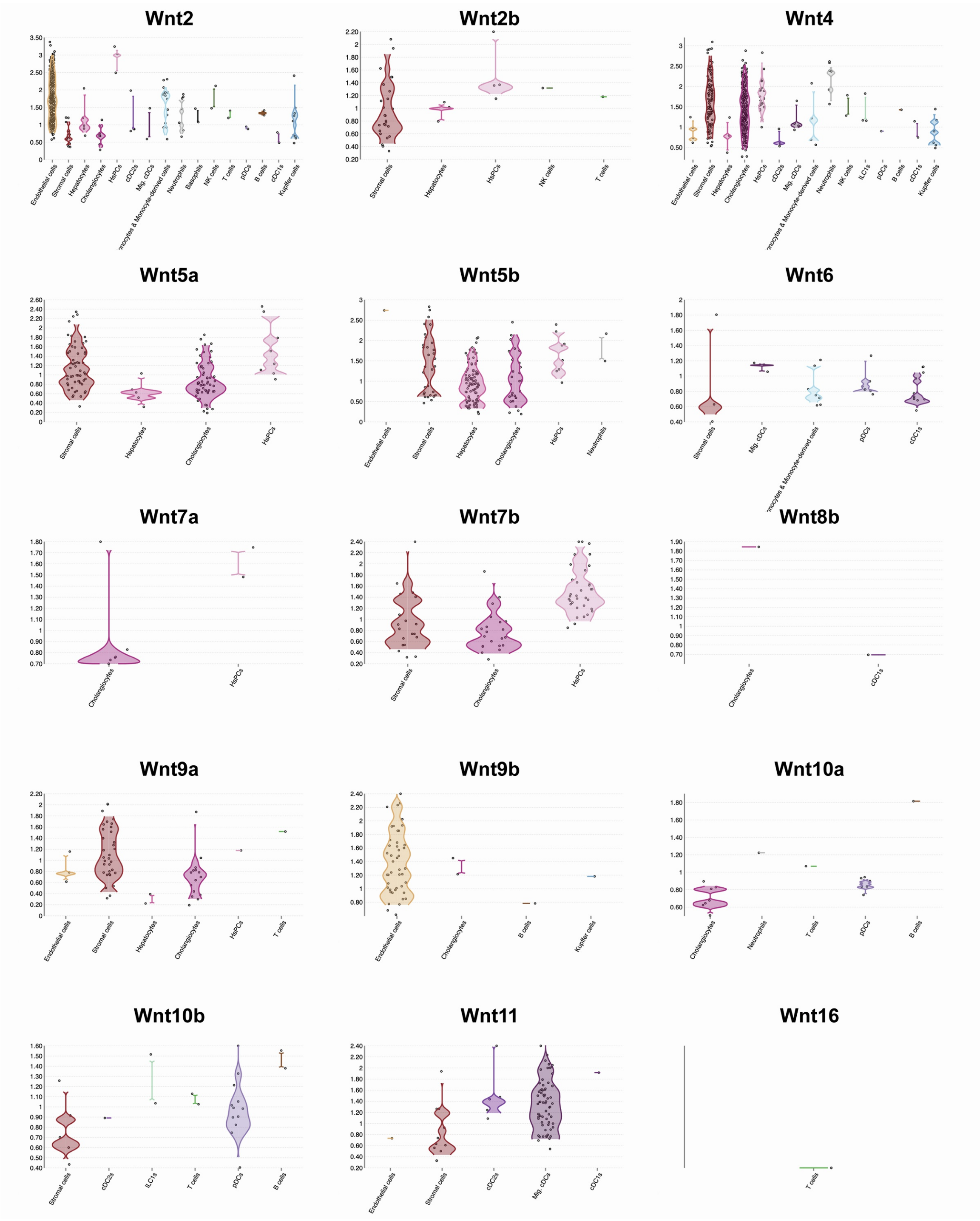

Fig. S4: Violin plots showing expression level of Wnts in C57BL/6 mice liver.
(Related to Figure 1)
Fifteen Wnts were detected. ECs expressed high levels of Wnt2 and Wnt9b.

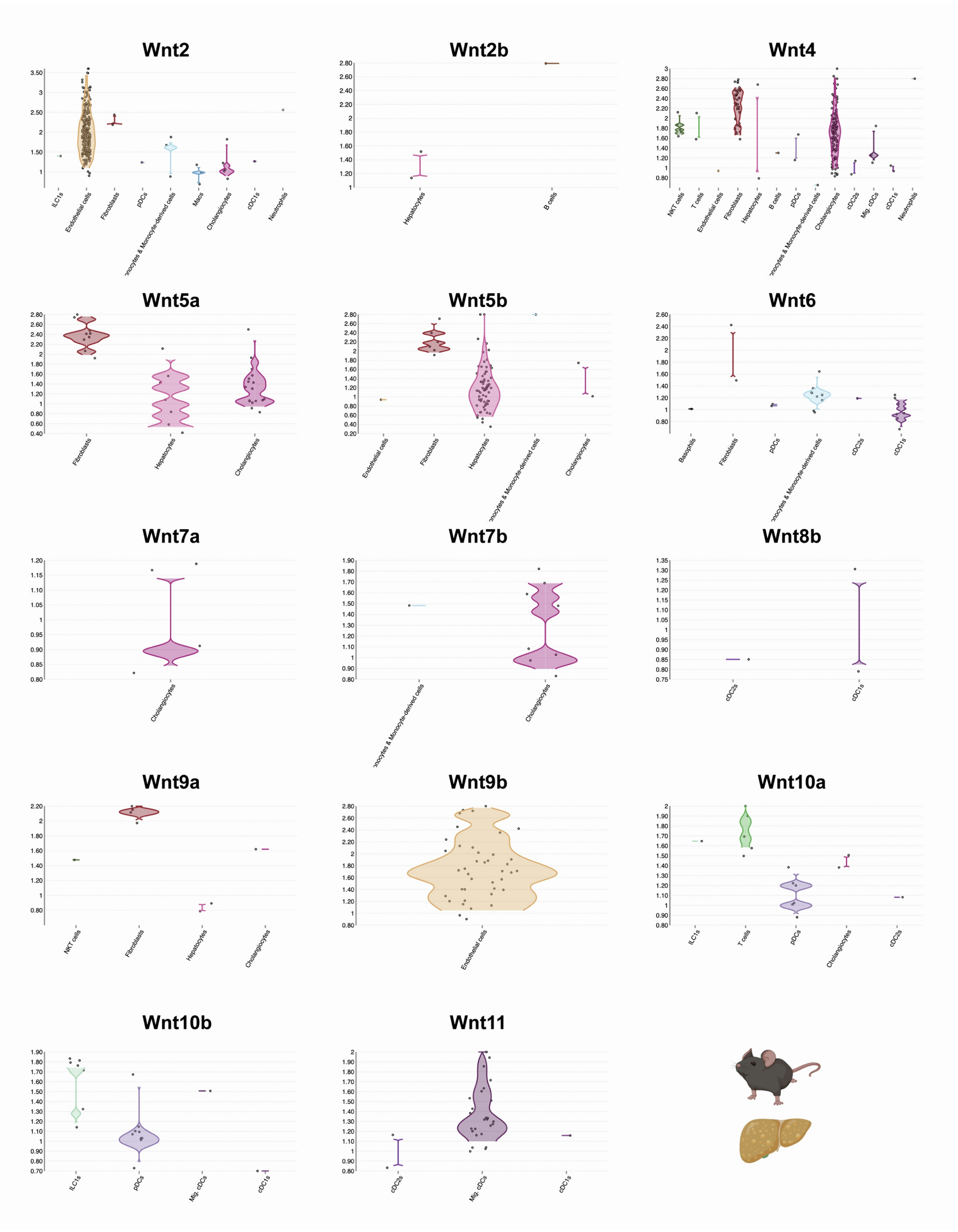

Fig. S5: Violin plots showing expression level of Wnts in NAFLD mice liver.
(Related to Figure 1)

Fourteen Wnts were detected. Endothelial expression of Wnt2 and Wnt9b was maintained in NAFLD mice. (Cartoons were created with BioRender.com)

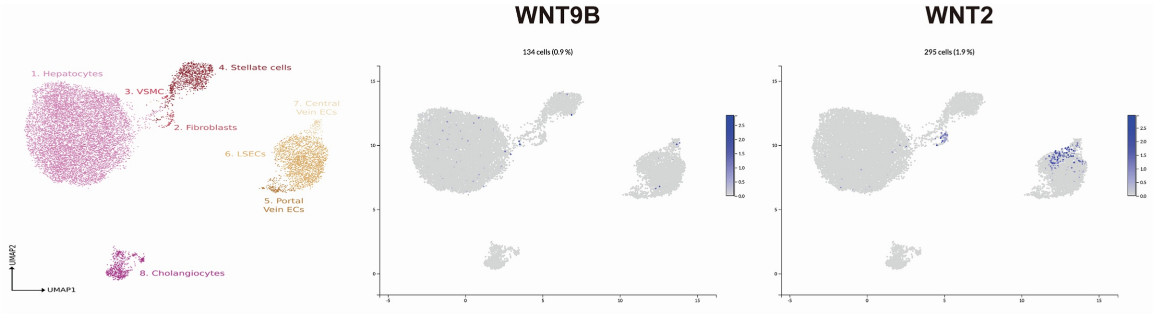

Fig. S6: Feature plots showing expression and distribution of WNTs in human liver. (Related to Figure 1)

WNT2 was pericentrally zonated in human ECs.

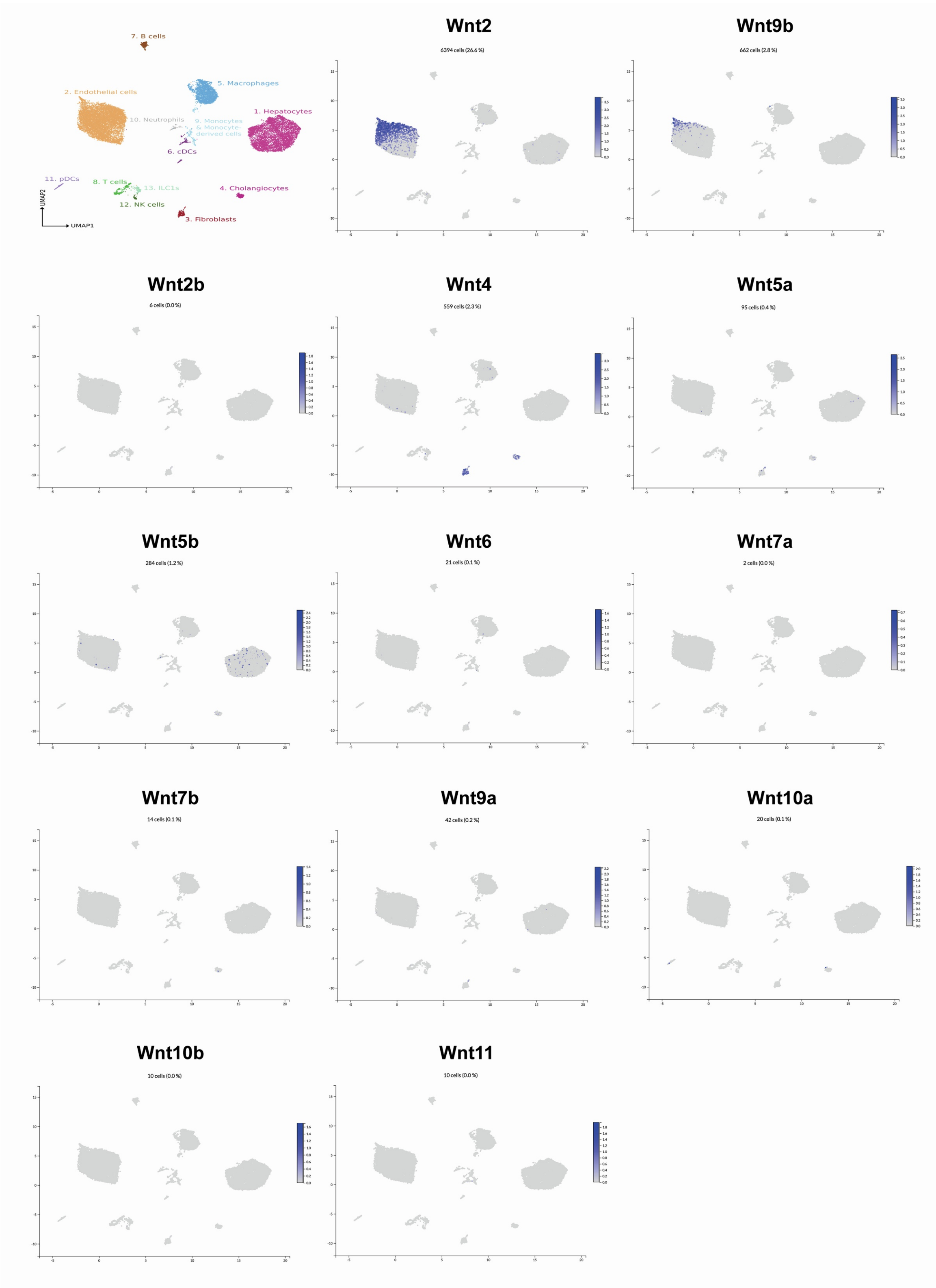

Fig. S7: Feature plots showing expression and distribution of Wnts in C57BL/6 mice liver using single nuclei RNA sequencing. (Related to Figure 1)
Among thirteen detected Wnts, only Wnt2 and Wnt9b were pericentrally zonated by snRNA seq.

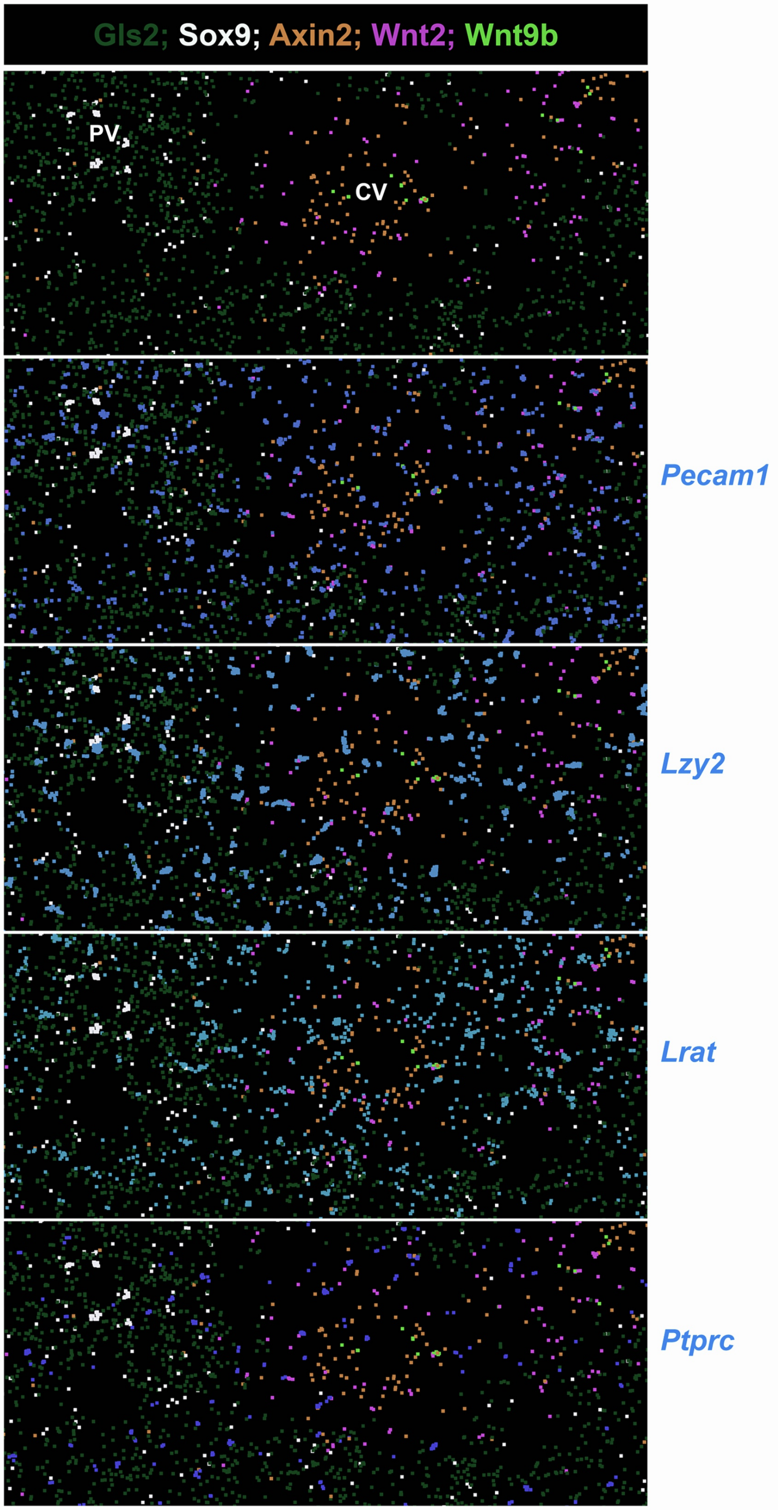

Fig. S8: Molecular Cartography of indicated genes. (Related to Figure 1)
Colocalization of *Wnt2* and *Wnt9b* with cell-specific markers including *Pecam1* for ECs, *Lyz2* for macrophages, *Lrat* for hepatic stellate cells, and *Ptprc* for immune cells. *Wnt2* and *Wnt9b* predominantly colocalized with *Pecam1*, while some overlap was also evident with *Lyz2*, *Lrat*, and *Ptprc*. (CV: Central vein; PV: portal vein)

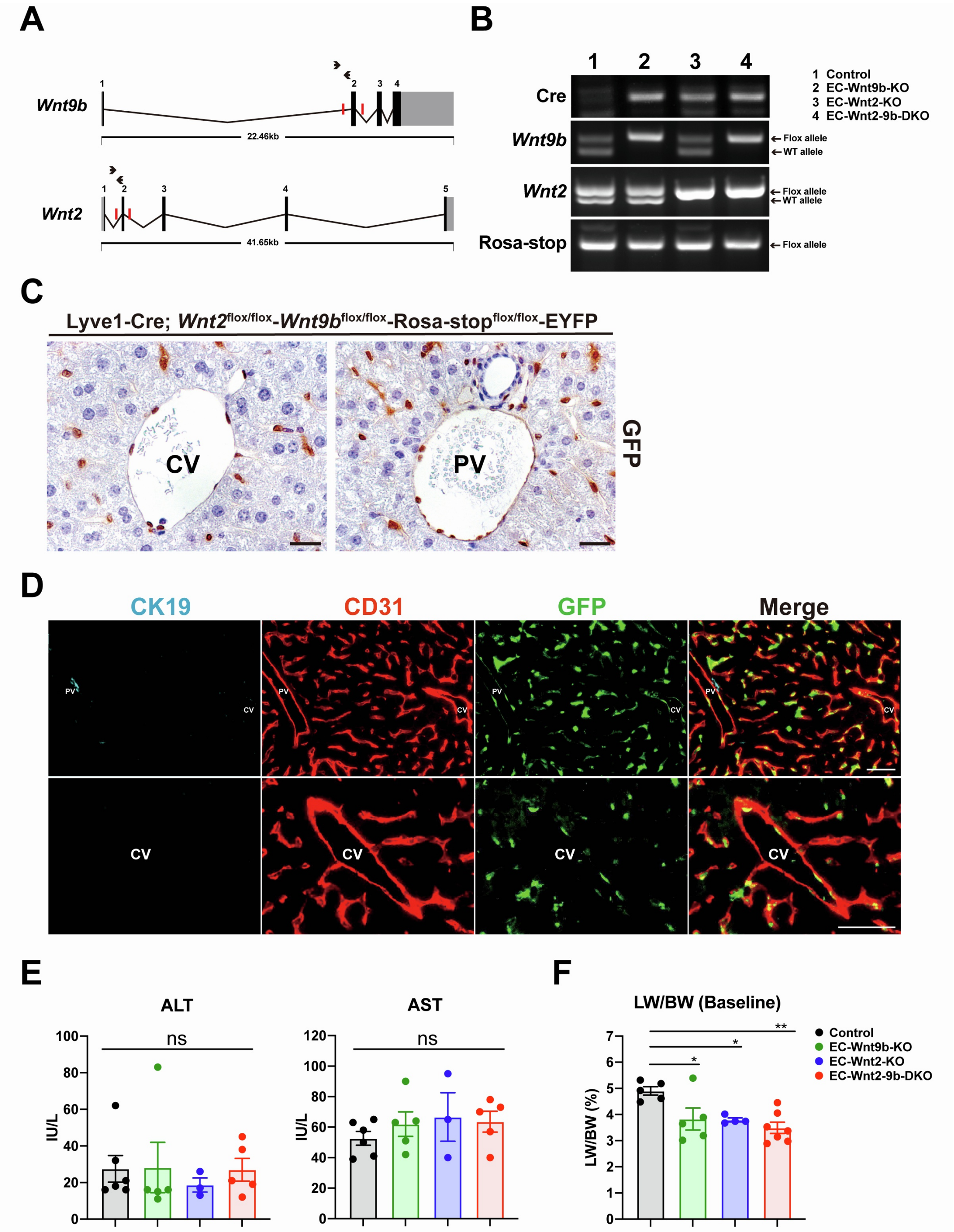

Fig. S9: Characterization of mice models. (Related to Figure 2)

(A) Scheme depicting loxP site (Red lines) in mice *Wnt9b* and *Wnt2* gene. (Black boxes: exons; grey boxes: 5'-UTR and 3'-UTR non-coding regions; arrow heads: PCR primers for genotyping)

(B) Gel electrophoresis showing generation of mice as indicated in the figure.

(C, D) IHC and immunofluorescence of EC-Wnt2-9b-DKO mice showing hepatic sinusoidal ECs and central venous ECs were positive for GFP. (CV: central vein; PV: portal vein. Scale bars for IHC: 20 µm; Scale bars for IF: 100 µm)

(E) No baseline liver injury was observed by serum levels of ALT and AST. (n = 6, 5, 3, 5 mice)

(F) LW/BW (+/-SEM) was lower in EC-Wnt2-KO and EC-Wnt9b-KO mice, and even lower in EC-Wnt2-9b-DKO mice. (ns = not significant, *$P < 0.05$, **$P < 0.01$. n = 5, 5, 4, 7 mice)

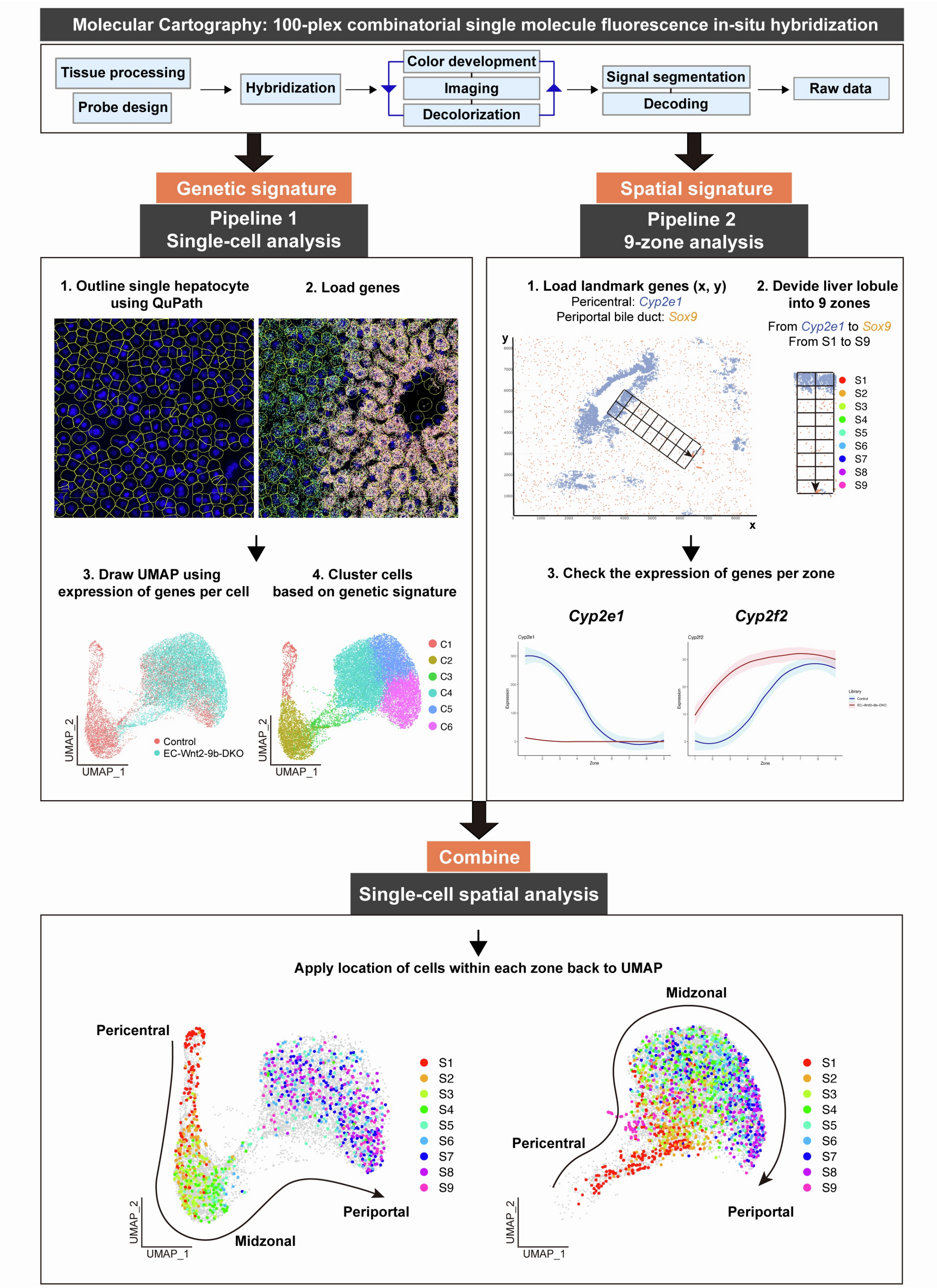

Fig. S10: Workflow of single-cell transcriptomics analysis of Molecular Cartography data of EC-Wnt2-9b-DKO mice. (Related to Figure 3 and STAR Methods)

See STAR Methods for details.

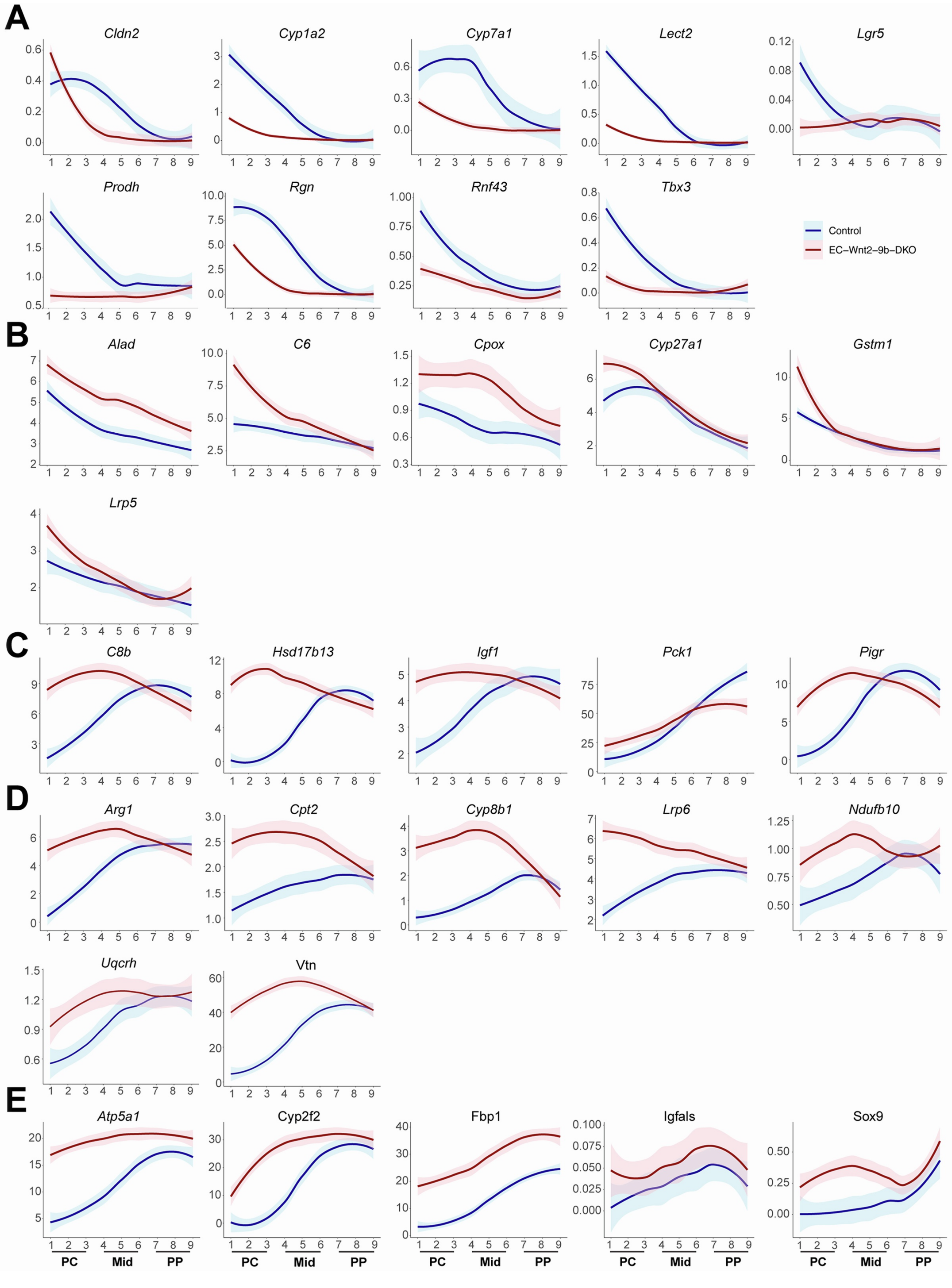

Fig. S11: Line plots showing changes of zonated genes between control and EC-Wnt2-9b-DKO mice. (Related to Figure 3)

(A) Pericentral genes that decreased in EC-Wnt2-9b-DKO mice.
(B) Pericentral genes that increased in EC-Wnt2-9b-DKO mice.
(C) Periportal genes that had decreased zone 1 gene expression in EC-Wnt2-9b-DKO mice.
(D) Periportal genes that had similar or unchanged zone 1 gene expression in EC-Wnt2-9b-DKO mice.
(E) Periportal genes that had increased zone 1 gene expression in EC-Wnt2-9b-DKO mice. The hue around line plots represents SEM.

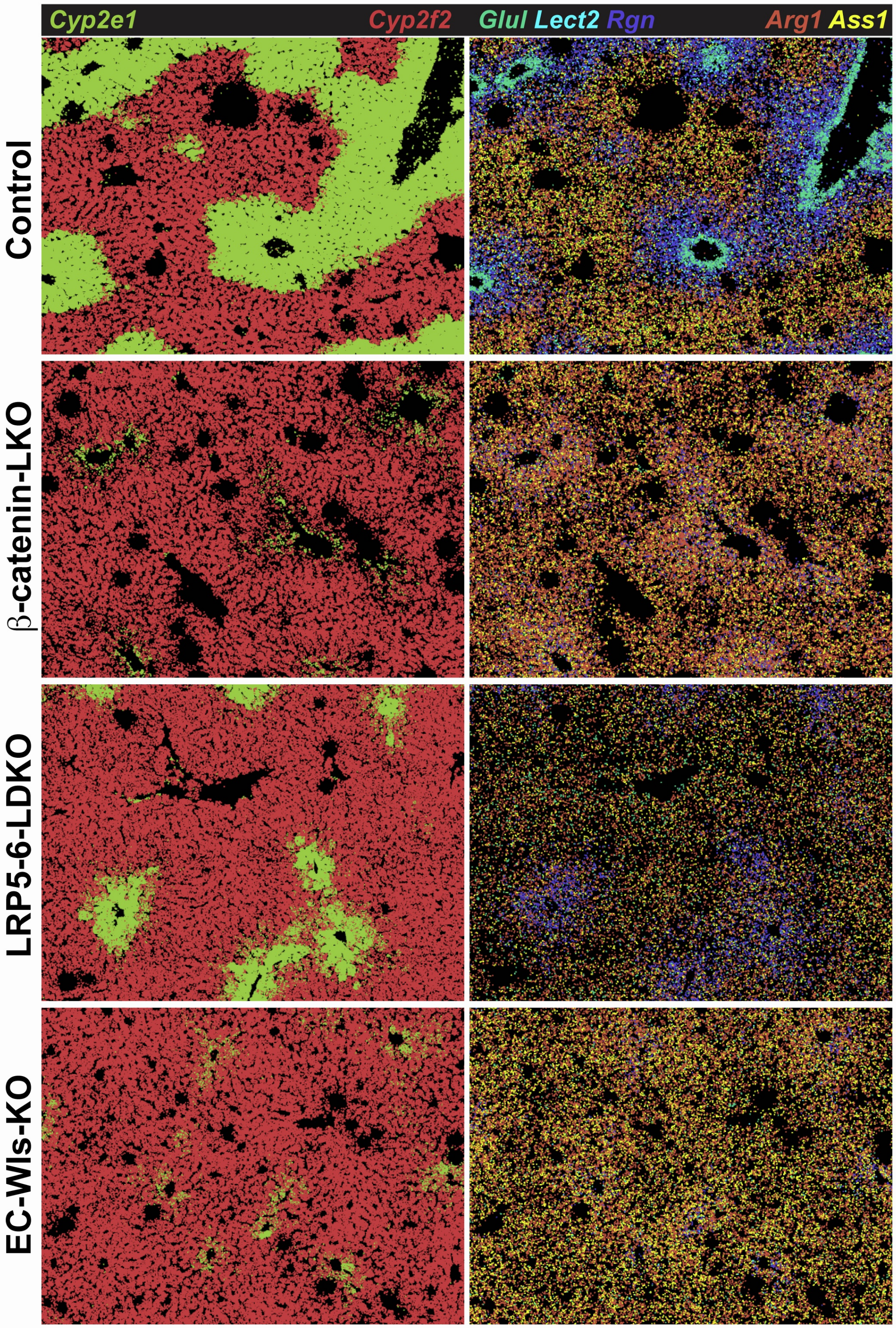

Fig. S12: Molecular Cartography images showing periportalization of liver lobule in various animal models of disrupted Wnt-β-catenin signaling (Related to Figure 3).

Representative images from Molecular Cartography analysis of selected genes in the livers from control, hepatocyte-specific β-catenin KO (β-catenin-LKO), hepatocyte-specific LRP5-6 DKO (LRP5-6-LDKO) and endothelial cell Wntless KO (EC-Wls-KO) showing gain of periportal genes in pericentral hepatocytes at the expense of pericentral β-catenin target genes.

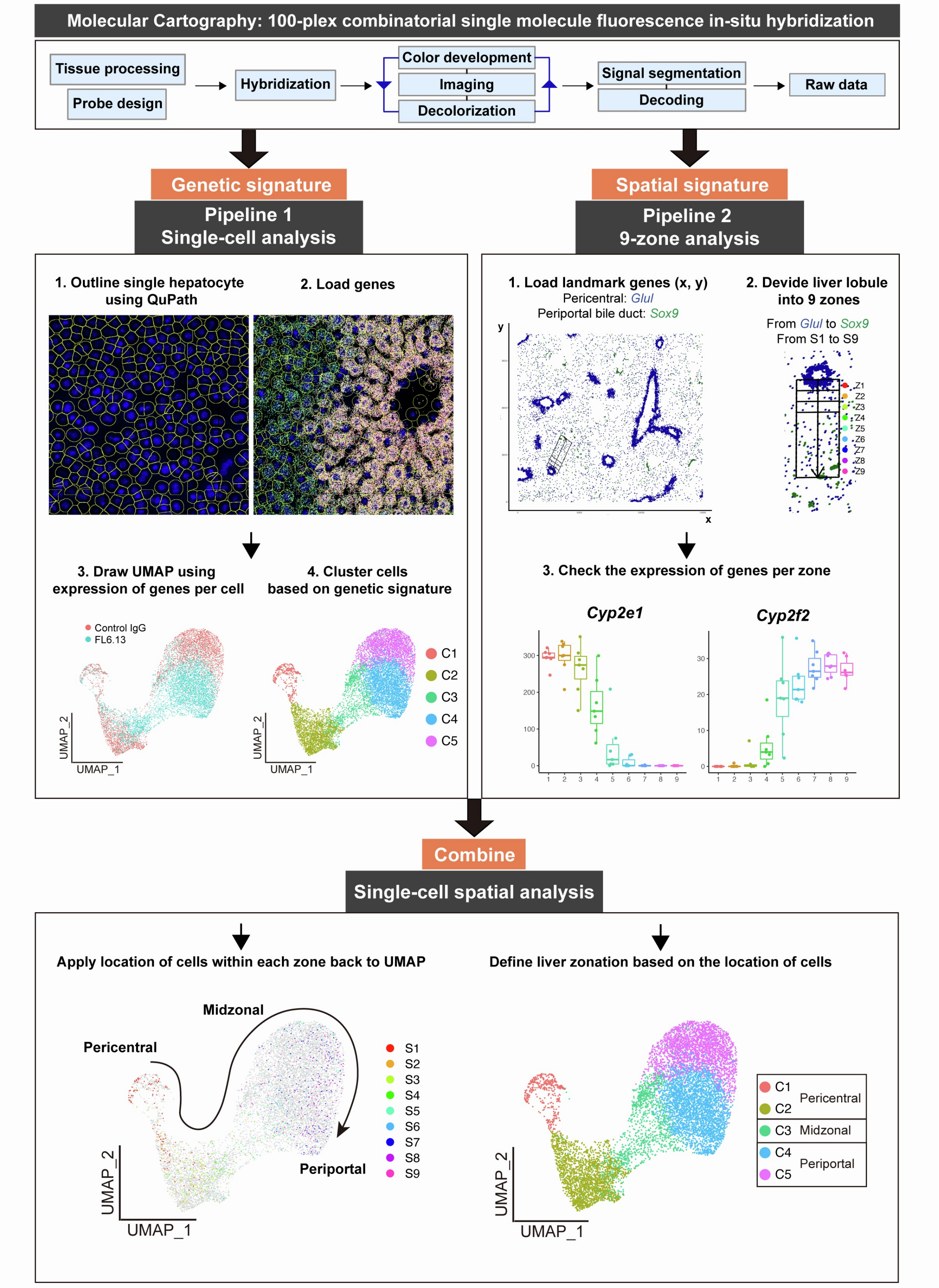

Fig. S13: Workflow of single-cell transcriptomics analysis of Molecular Cartography data of FL6.13-treated mice. (Related to Figure 6 and STAR Methods)

See STAR Methods for details.

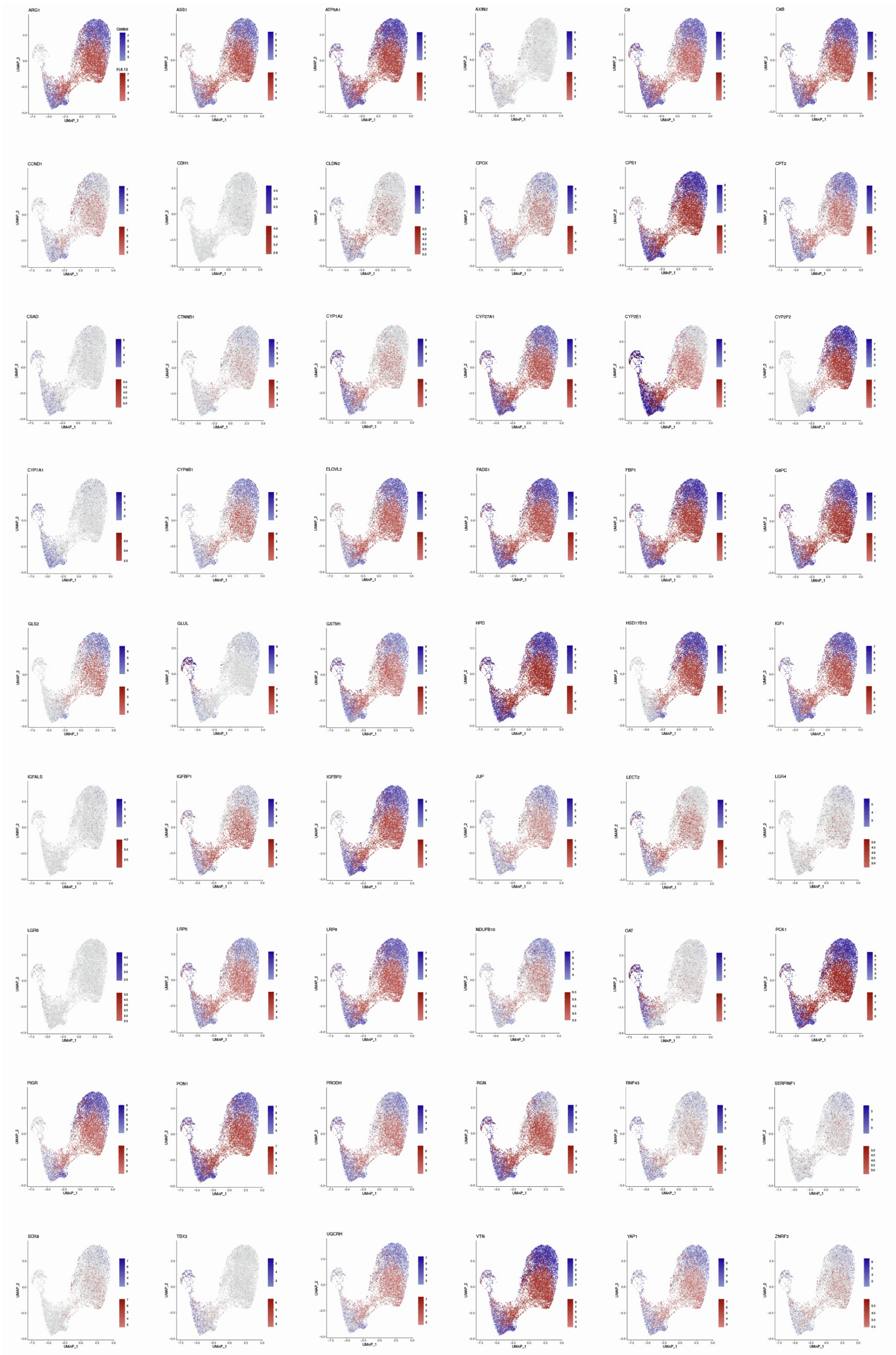

Fig. S14: Feature plots showing expression of genes in control and FL6.13-treated animal. (Related to Figure 6)
Blue: Control IgG; Red: FL6.13 treatment

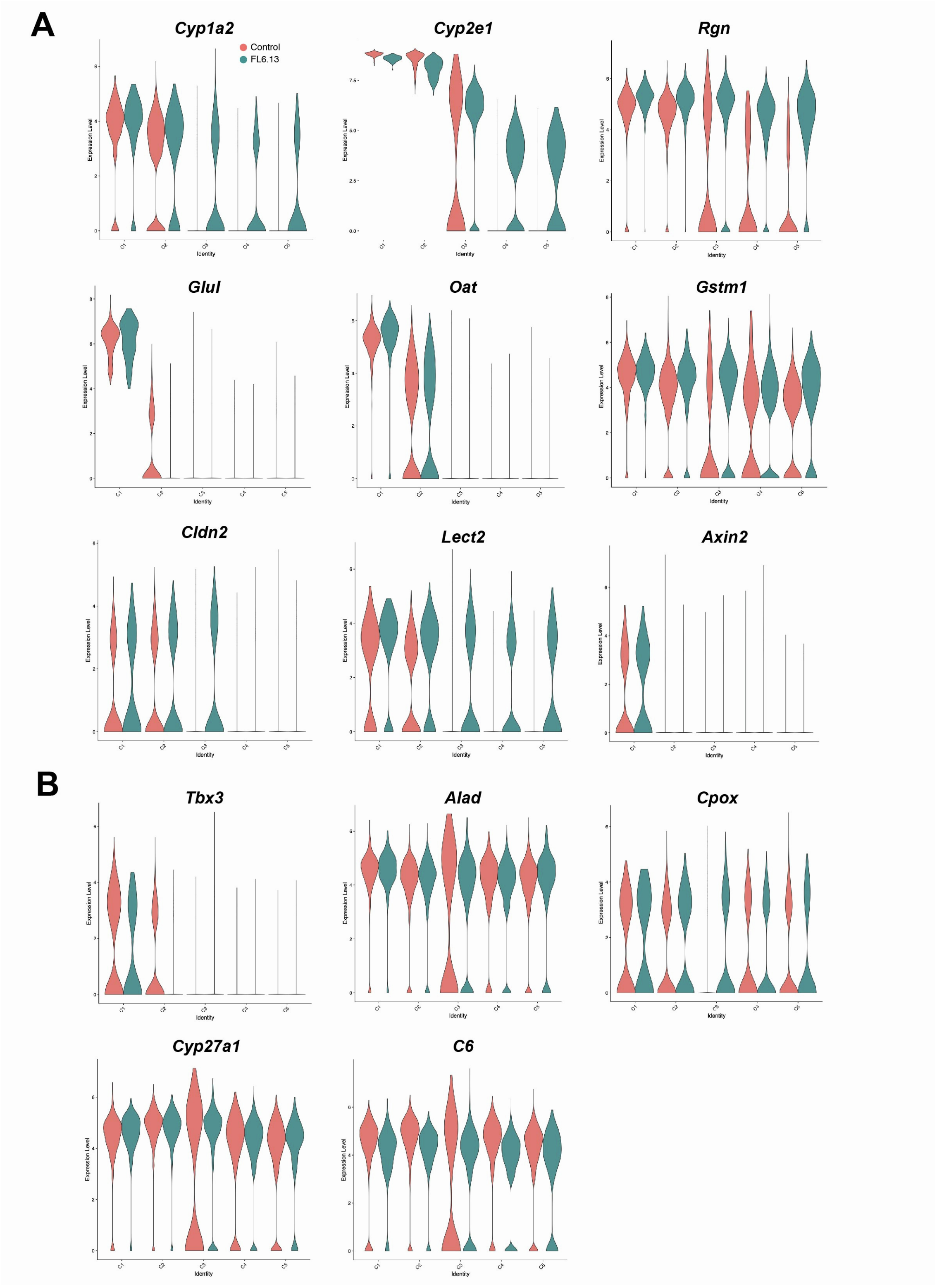

Fig. S15: Expression level of pericentral zonated genes in control and FL6.13-treated liver. (Related to Figure 6)

(A) Violin plots showing expanded expression of Wnt target genes after FL6.13 treatment.

(B) Violin plots showing genes that were not increased after FL6.13 treatment.

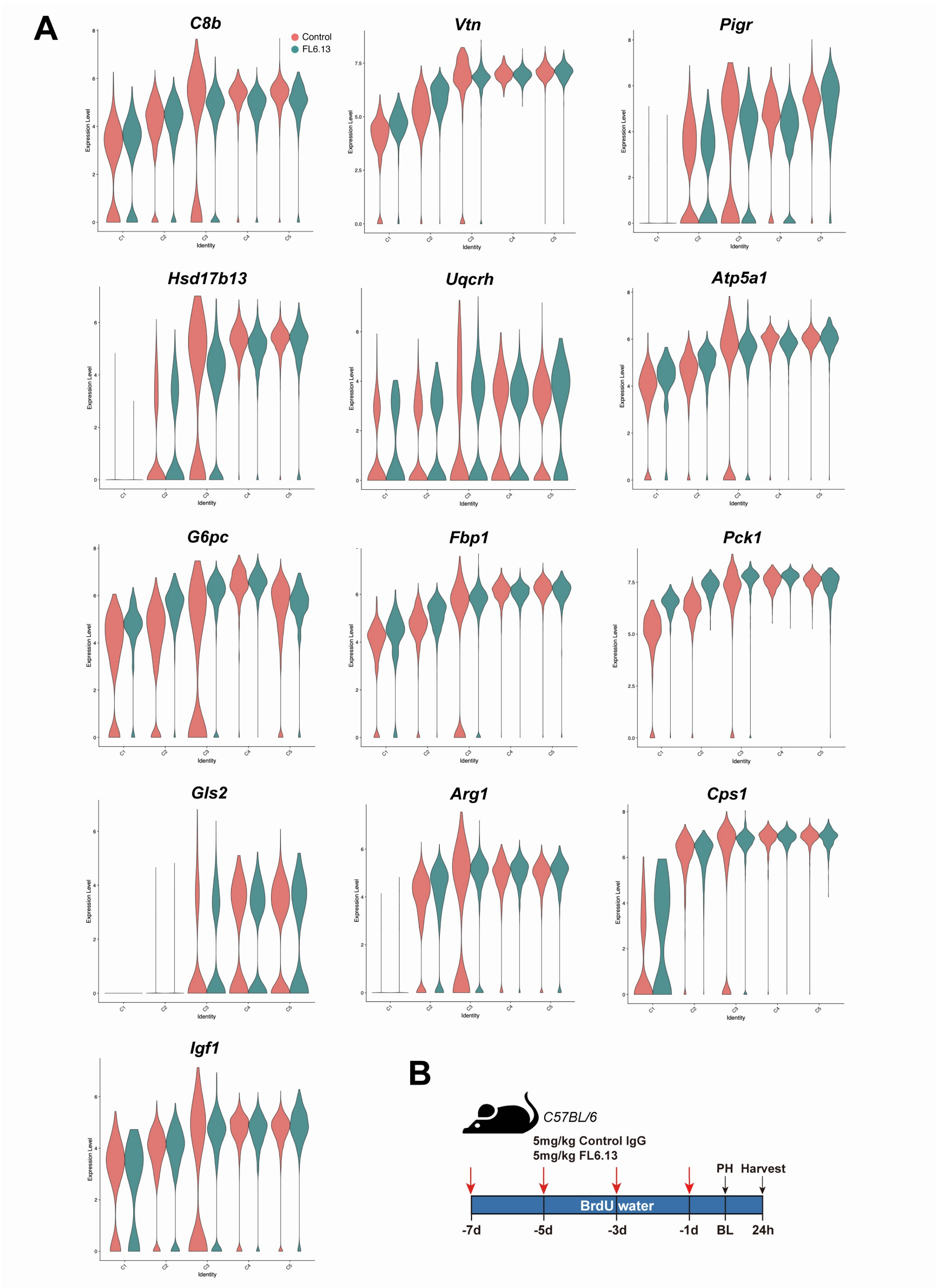

Fig. S16: Expression level of periportal zonated genes in control and FL6.13-treated liver. (Related to Figure 6)

(A) Violin plots showing that expression of periportal genes was not decreased after FL6.13 treatment.

(B) Study design of control IgG and FL6.13 treatment for regeneration study.

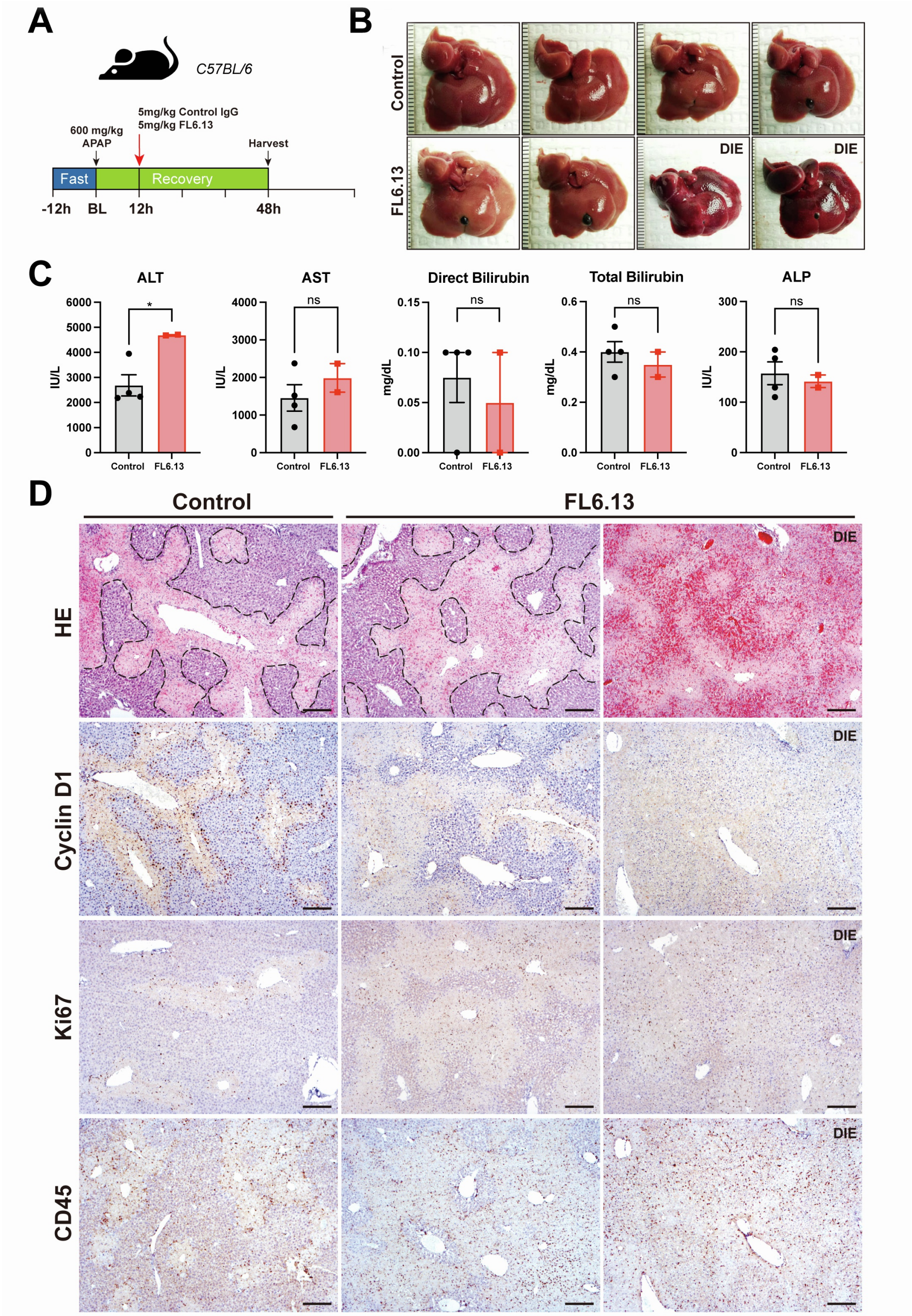

Fig. S17: Early treatment with FL6.13 worsens liver injury after acetaminophen overdose. (Related to Figure 7)

(A) Study design showing dosing schedule of pan-FZD agonist FL6.13 or isotype control IgG administration. Single dose of 5 mg/kg control IgG or FL6.13 was i.p. administrated to mice at 12 hours post 600 mg/kg i.p. APAP injection. Mice were sacrificed at 48 hours for analysis.

(B) Gross images of livers showing necrosis and congestion in control IgG and FL6.13-treated animals at 48 hours.

(C) Serum levels of ALT, AST, direct bilirubin, total bilirubin, and ALP at 48 hours showing increased liver injury when FL6.13 was given at 12 hours. (The bars represent means ± SEM. ns = not significant, *$P < 0.05$. n = 4, 2 mice)

(D) Representative IHC showing necrotic areas by HE, cell proliferation by Cyclin D1 and Ki67, and immune cell infiltration by CD45.

Supplementary Tables

Table S1: Sequence of genotyping primers (related to STAR Methods)

**Primer name**	**Sequence (5’-3’)**
Lyve1-Cre-F_wt	TGCCACCTGAAGTCTCTCCT
Lyve1-Cre-F_mutant	GAGGATGGGGACTGAAACTG
Lyve1-Cre-R	TGAGCCACAGAAGGGTTAGG
ROSA-EYFP-F_wt	GGAGCGGGAGAAATGGATATG
ROSA-EYFP-F_mutant	AAGACCGCGAAGAGTTTGTC
ROSA-EYFP-R	AAAGTCGCTCTGAGTTGTTAT
Wnt9b-F	GCAGAATCTGGAGAACTTGGC
Wnt9b-R	GTGAGAAGGAAGATGGTGAGC
Wnt2-F	CCCAGCAGGTGCTAAGAGG
Wnt2-R	CAATGGCACGCATCACATCT

Table S2: Size of PCR products (related to STAR Methods)

Genotype	WT (bp)	Mutant (bp)
Lyve1-Cre	425	210
Rosa-EYFP	600	324
Wnt9b	219	300
Wnt2	540	592

Table S3: Probe list for Molecular Cartography™ (related to STAR Methods)

Item	Cat. No	Species	Design Target ID	Gene Name
1	P0D1P	Mus musculus	ENSMUSG00000006932	Ctnnb1
2	P0DCL	Mus musculus	ENSMUSG00000015957	Wnt11
3	P0ECZ	Mus musculus	ENSMUSG00000002588	Pon1
4	P1DC0	Mus musculus	ENSMUSG00000029671	Wnt16
5	P2D1R	Mus musculus	ENSMUSG00000024913	Lrp5
6	P2DC1	Mus musculus	ENSMUST00000054294	Fzd1
7	P2Y49	Mus musculus	ENSMUSG00000000303	Cdh1
8	P3DC2	Mus musculus	ENSMUSG00000022297	Fzd6
9	P3EC1	Mus musculus	ENSMUST00000102840	Ass1
10	P3F4T	Mus musculus	ENSMUSG00000004730	Adgre1
11	P4DC3	Mus musculus	ENSMUST00000114246	Fzd7
12	P4EC2	Mus musculus	ENSMUST00000020161	Arg1
13	P5DC4	Mus musculus	ENSMUST00000041080	Fzd8
14	P5EC3	Mus musculus	ENSMUST00000045602	Ndufb10
15	P638C	Mus musculus	ENSMUST00000092163	Lyz2
16	P6787	Mus musculus	ENSMUST00000019469	G6pc
17	P6C1X	Mus musculus	ENSMUSG00000020053	Igf1
18	P6DC5	Mus musculus	ENSMUST00000032322	Lrp6
19	P7DC6	Mus musculus	ENSMUSG00000050199	Lgr4
20	P7EC5	Mus musculus	ENSMUST00000092888	Fbp1
21	P8DC7	Mus musculus	ENSMUSG00000034177	Rnf43
22	P8EC6	Mus musculus	ENSMUST00000050714	Igfals
23	P948E	Mus musculus	ENSMUST00000003100	Cyp2f2
24	P9DC8	Mus musculus	ENSMUSG00000041961	Znrf3
25	P9M7Y	Mus musculus	ENSMUST00000092623	Rspo3
26	PAN7Y	Mus musculus	ENSMUST00000044776	Gls2
27	PCN7Z	Mus musculus	ENSMUST00000029017	Pck1
28	PDECA	Mus musculus	ENSMUSG00000021364	Elovl2
29	PEECC	Mus musculus	ENSMUSG00000010663	Fads1
30	PEV7V	Mus musculus	ENSMUST00000029905	Cyp7a1
31	PFE89	Mus musculus	ENSMUST00000117102	Fzd10
32	PFG43	Mus musculus	ENSMUSG00000026395	Ptprc
33	PFVCL	Mus musculus	ENSMUST00000057893	Fzd2
34	PGC15	Mus musculus	ENSMUSG00000022382	Wnt7b
35	PGECE	Mus musculus	ENSMUSG00000029656	C8b
36	PGVC0	Mus musculus	ENSMUSG00000001552	Jup
37	PHE8C	Mus musculus	ENSMUST00000131309	Fzd3
38	PHVC1	Mus musculus	ENSMUSG00000024182	Axin1
39	PJDCH	Mus musculus	ENSMUST00000024954	Epas1
40	PJE8D	Mus musculus	ENSMUST00000058755	Fzd4
41	PJM75	Mus musculus	ENSMUST00000010941	Wnt2
42	PJR71	Mus musculus	ENSMUST00000093962	Ccnd1

43	PJVC2	Mus musculus	ENSMUSG00000005871	Apc
44	PKCCK	Mus musculus	ENSMUST00000023734	Wnt1
45	PKDCJ	Mus musculus	ENSMUSG00000015522	Arnt
46	PKE8E	Mus musculus	ENSMUSG00000045005	Fzd5
47	PKM76	Mus musculus	ENSMUST00000018630	Wnt9b
48	PKT6L	Mus musculus	ENSMUST00000000579	Sox9
49	PKVC3	Mus musculus	ENSMUSG00000031169	Porcn
50	PMA5F	Mus musculus	ENSMUSG00000028393	Alad
51	PMCCM	Mus musculus	ENSMUST00000029429	Wnt2b
52	PMDCK	Mus musculus	ENSMUST00000060077	Cpox
53	PMECJ	Mus musculus	ENSMUST00000017488	Vtn
54	PMP1L	Mus musculus	ENSMUSG00000039323	Igfbp2
55	PMVC4	Mus musculus	ENSMUSG00000028173	Wls
56	PNCCN	Mus musculus	ENSMUST00000000127	Wnt3
57	PNDCM	Mus musculus	ENSMUSG00000022181	C6
58	PNVC5	Mus musculus	ENSMUSG00000018569	Cldn7
59	PPCCP	Mus musculus	ENSMUST00000010044	Wnt3a
60	PPDCN	Mus musculus	ENSMUST00000054889	Cldn2
61	PPHCH	Mus musculus	ENSMUSG00000034528	Hsd17b13
62	PPVC6	Mus musculus	ENSMUSG00000021539	Lect2
63	PQCCQ	Mus musculus	ENSMUST00000045747	Wnt4
64	PQE8J	Mus musculus	ENSMUST00000062572	Fzd9
65	PQVC7	Mus musculus	ENSMUSG00000003526	Prodh
66	PR47V	Mus musculus	ENSMUSG00000053110	Yap1
67	PR56S	Mus musculus	ENSMUSG00000018604	Tbx3
68	PR81H	Mus musculus	ENSMUSG00000020717	Pecam1
69	PRCCR	Mus musculus	ENSMUSG00000021994	Wnt5a
70	PRVC8	Mus musculus	ENSMUST00000034860	Cyp1a2
71	PS68V	Mus musculus	ENSMUST00000027144	Cps1
72	PSCCS	Mus musculus	ENSMUST00000006716	Wnt6
73	PSDCR	Mus musculus	ENSMUST00000031398	Hpd
74	PSVC9	Mus musculus	ENSMUST00000023832	Rgn
75	PT86R	Mus musculus	ENSMUSG00000000753	Serpinf1
76	PTCCT	Mus musculus	ENSMUST00000032180	Wnt7a
77	PTDCS	Mus musculus	ENSMUST00000084500	Oat
78	PTF9N	Mus musculus	ENSMUST00000030687	Rspo1
79	PTVCA	Mus musculus	ENSMUST00000078676	Uqcrh
80	PV85R	Mus musculus	ENSMUSG00000025428	Atp5a1
81	PVCCV	Mus musculus	ENSMUST00000012426	Wnt8a
82	PVF4H	Mus musculus	ENSMUSG00000025479	Cyp2e1
83	PWCCW	Mus musculus	ENSMUST00000041163	Wnt8b
84	PWDCV	Mus musculus	ENSMUSG00000019838	Slc16a10
85	PWF4J	Mus musculus	ENSMUSG00000020140	Lgr5
86	PWVCD	Mus musculus	ENSMUSG00000028607	Cpt2

87	PXCCX	Mus musculus	ENSMUSG00000000126	Wnt9a
88	PXDCW	Mus musculus	ENSMUST00000062474	Cyp8b1
89	PXF4K	Mus musculus	ENSMUSG00000000142	Axin2
90	PXV8A	Mus musculus	ENSMUSG00000023044	Csad
91	PXVCE	Mus musculus	ENSMUST00000027675	Pigr
92	PXWCD	Mus musculus	ENSMUSG00000058135	Gstm1
93	PYCCY	Mus musculus	ENSMUST00000006718	Wnt10a
94	PYDCX	Mus musculus	ENSMUST00000027356	Cyp27a1
95	PYWCE	Mus musculus	ENSMUST00000020704	Igfbp1
96	PZ78L	Mus musculus	ENSMUST00000029632	Lrat
97	PZ81R	Mus musculus	ENSMUSG00000026473	Glul
98	PZ85W	Mus musculus	ENSMUSG00000021109	Hif1a
99	PZCAY	Mus musculus	ENSMUSG00000030170	Wnt5b
100	PZCCZ	Mus musculus	ENSMUSG00000022996	Wnt10b

Table S4: Sequence of qPCR primers (related to STAR Methods)

Gene	Forward	Reverse
Rn18s	GTAACCCGTTGAACCCCATT	CCATCCAATCGGTAGTAGCG
Glul	CTCGCTCTCCTGACCTGTTC	TTCAAGTGGGAACTTGCTGA
Cyp2e1	AATGGACCTACCTGGAAGGAC	CCTCTGGATCCGGCTCTCATT
Axin2	TGACTCTCCTTCCAGATCCCA	TGCCCACACTAGGCTGACA
Lect2	CCCACAACAATCCTCATTTCA	GTTAGCCCATGGTCCTGCTA
Oat	CCGACCAGTTATGATGGCTTTGG	CTCCACCATGAAGGCAGCAACA
Rgn	GTATGGGAGGAAGCGTCACAGT	CAATGGTGGCAACATAGCCTCC
Cldn2	GCAAACAGGCTCCGAAGATACT	GAGATGATGCCCAAGTACAGAG
Gstm1	ATACTGGGATACTGGAACGTCC	AGTCAGGGTTGTAACAGAGCAT
Lgr5	CCTACTCGAAGACTTACCCAGT	GCATTGGGGTGAATGATAGCA
Tbx3	ACTCGGGGTCGGAACTGAA	GGAGGGGGCGATTTTGTTTTT
G6pc	CAGTGGTCGGAGACTGGTTC	TATAGGCACGGAGCTGTTGC
Pck1	TGTCTTCACTGAGGTGCCAG	CTGGATGAAGTTTGATGCCC
Arg1	ACAAGACAGGGCTCCTTTCAG	TGAGTTCCGAAGCAAGCCAA
Cps	AGGATGTCAAGGTGTTTGGC	GCTTAACTAGCAGGCGGATG
Ass1	ACACCTCCTGCATCCTCGT	GCTCACATCCTCAATGAACACCT
Atp5a1	TGGTGAAGAGACTGACGGATGC	TCAAAGCGTGCTTGCCGTTGTC
Igf1	TCATGTCGTCTTCACACCTCTTCT	CCACACACGAACTGAAGAGCAT
C8b	ACTGTCAACGGGAGATGGAGCA	GTTGGTGTCCAGGATGTAGTGG
Cyp8b1	AGTACACATGGACCCCGACATC	GGGTGCCATCCGGGTTGAG
Hpd	CGCTCCATTGTGGTGACCAACT	TCCGTCTTGAGAGCGATGTGCT
Ccnd1	TTTCTTTCCAGAGTCATCAAGTGT	TGACTCCAGAAGGGCTTCAA
